# Supplementary material for: 3D-Printing of Capsule Devices as Compartmentalization Tools for Supported Reagents in the Search of Antiproliferative Isatins
Source: Pharmaceuticals (Basel). 2023 Feb 16;16(2):310. doi: 10.3390/ph16020310 (PMC9965165; doi:10.3390/ph16020310)
Supplement: Supplementary file 1 [file pharmaceuticals-16-00310-s001.zip › pharmaceuticals-2168054-supplementary.pdf]

3D-Printing of Capsule Devices as Compartmentalization Tools for Supported Reagents in the Search of Antiproliferative Isatins

Supplementary Material

Table of contents:

1. Figure S1. Detailed 3D-printing process of a bicompartmental capsule…………………S1
2. Figure S2: EDX and FESEM data for IRA-120-Cu(0) and IRA-Pd(0)………………………S2
3. RMN spectra of the related compounds…………………………………………………….S2
4. ESI-masas spectra of the related compounds……………………………………………...S14
5. X-ray crystalographic data of compound **17**………………………………………………S26

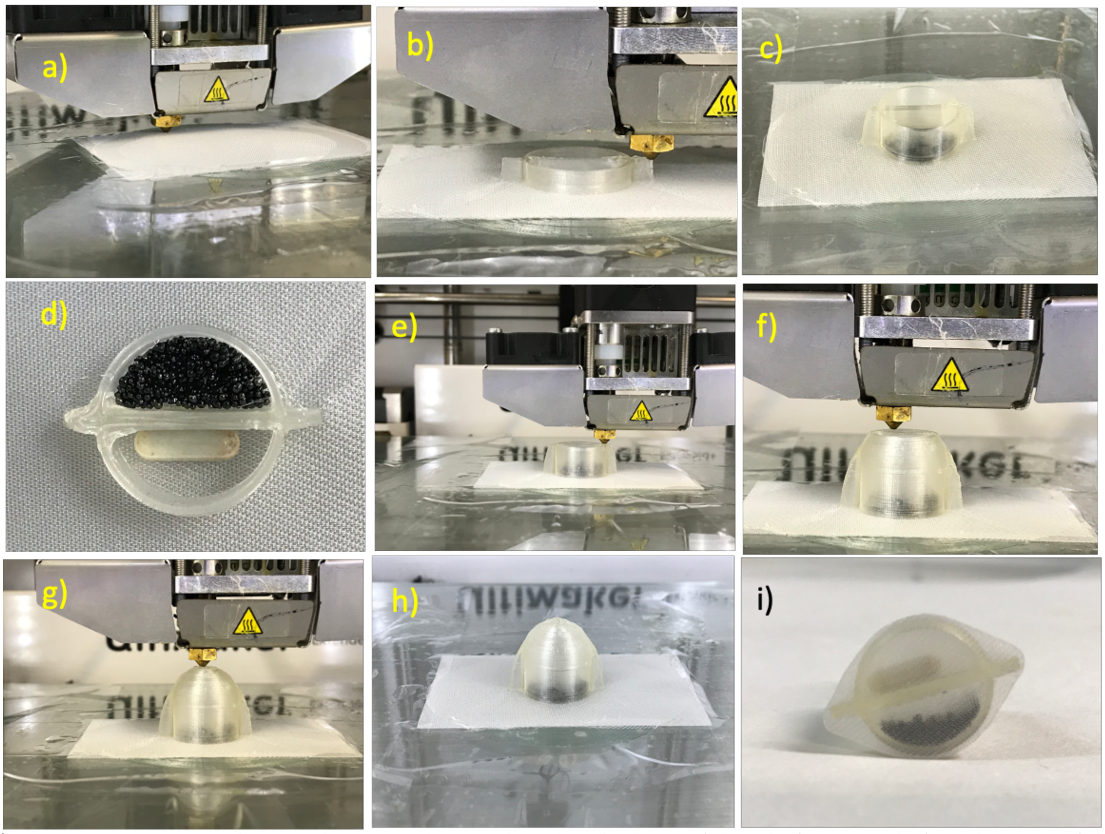

Figure S1. a) Porous polypropylene membrane placed at the base of the platform just before the start of the 3D-printing process. b) image at 5 min. c) moment of pause in the 3D-printing and loading of the catalytic material and the magnetic stirrer. d) Parietal view of the capsule at the time of paused loading. e, f) resumption of 3D-printing. g) final moment of 3D-printing (23 min). h) finished capsule. i) View of the porous area after cutting the excess membrane part with scissors.

Figure S2 a) EDX and FESEM data of IRA-120-Cu(0)

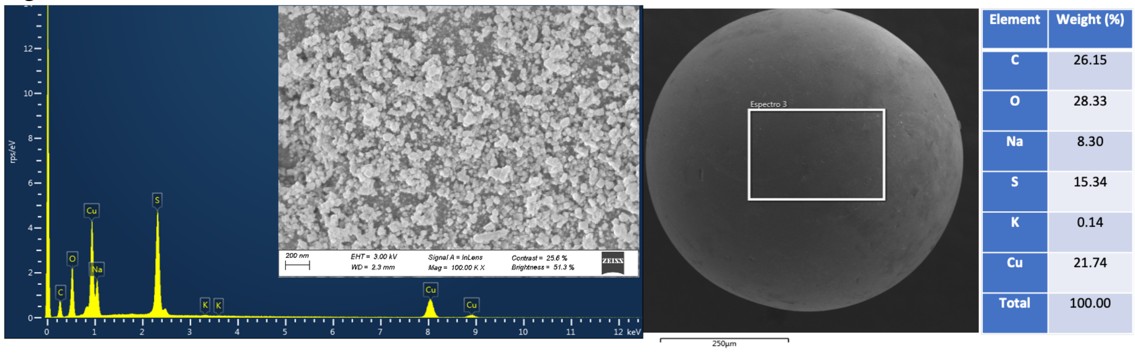

Element	Weight (%)
C	26.15
O	28.33
Na	8.30
S	15.34
K	0.14
Cu	21.74
Total	100.00

Figure S2 b) EDX and SEM data of IRA-120-Pd(0)

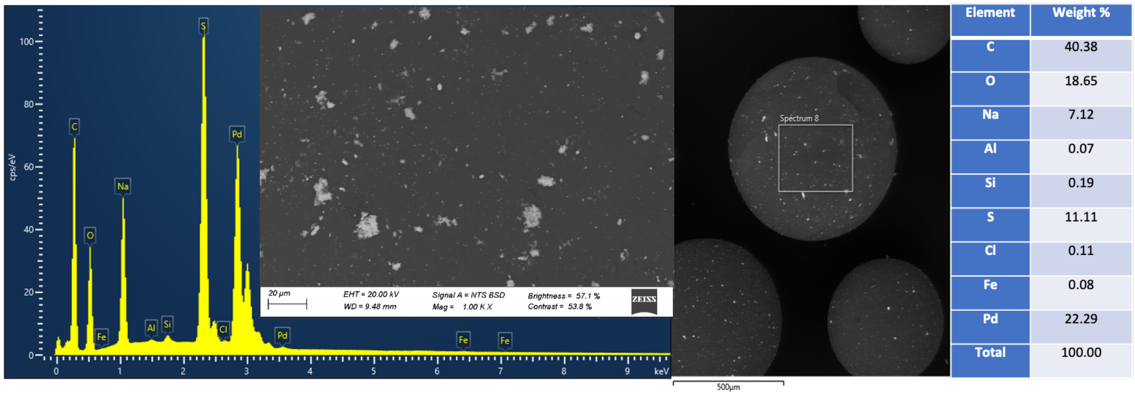

Element	Weight %
C	40.38
O	18.65
Na	7.12
Al	0.07
Si	0.19
S	11.11
Cl	0.11
Fe	0.08
Pd	22.29
Total	100.00

3. RMN spectra of the related compounds

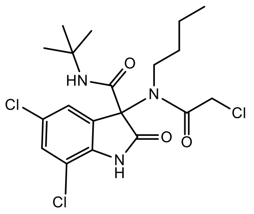

14

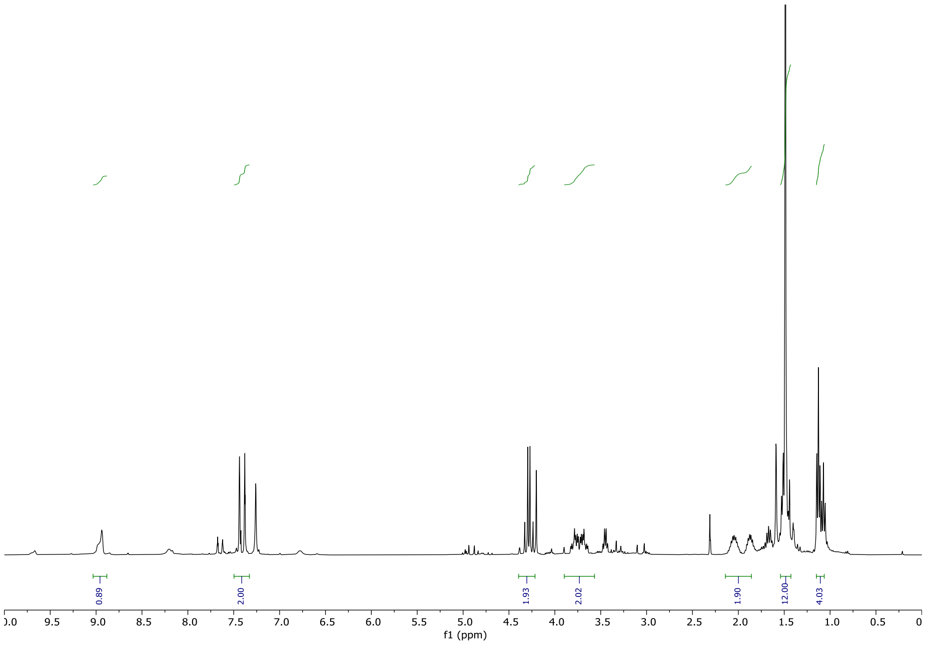

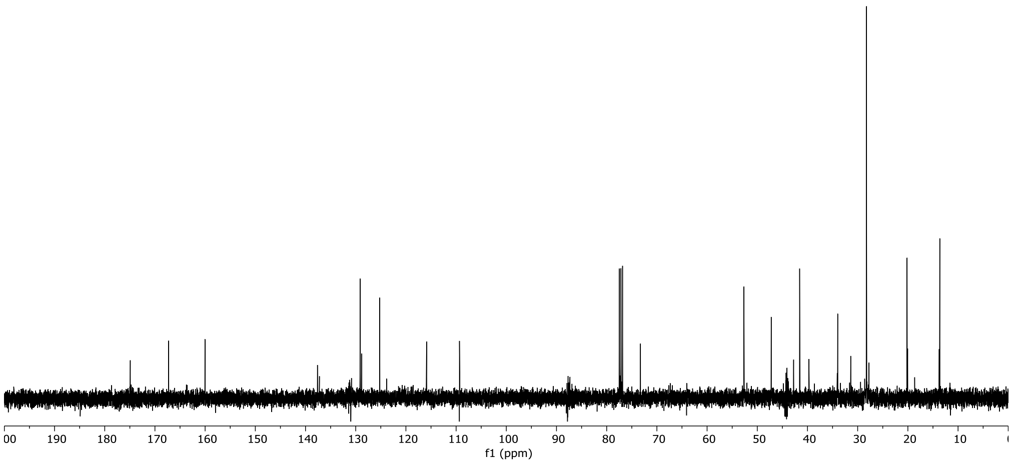

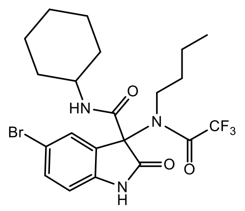

15

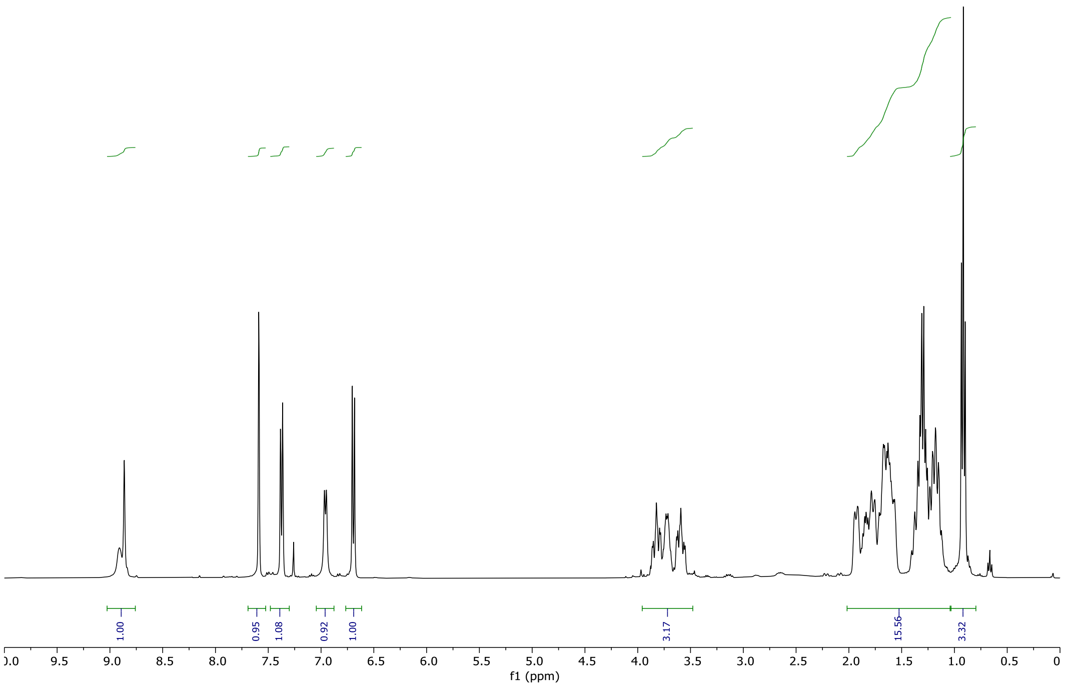

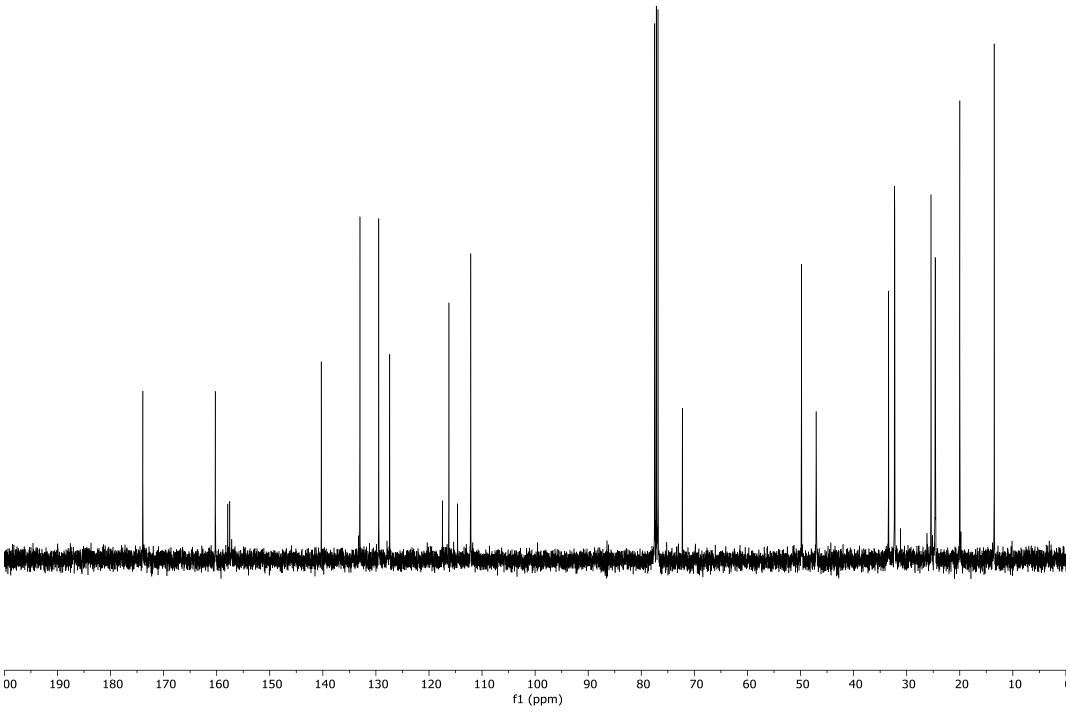

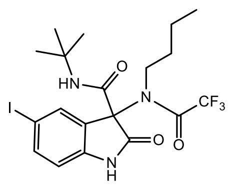
HN
O
N
CF3
I
O
O
NH

17

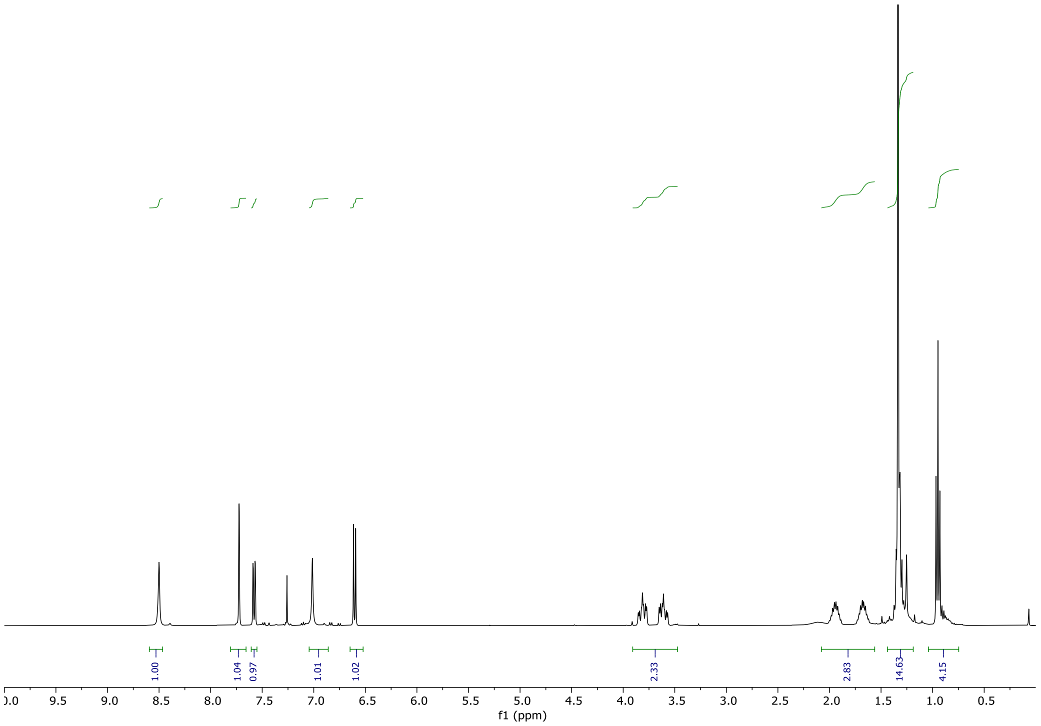
1.00
1.04
0.97
1.01
1.02
2.33
2.83
14.63
4.15
f1 (ppm)

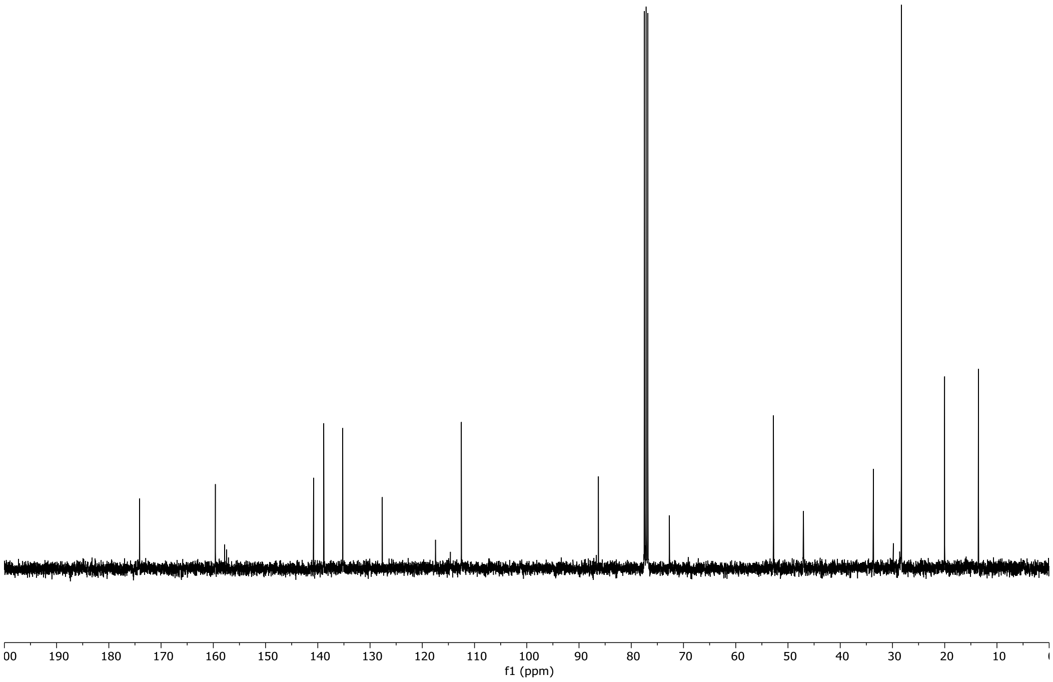
f1 (ppm)

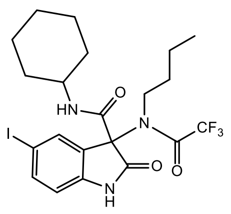
HN
O
N
CF3
I
O
NH
H
O

18

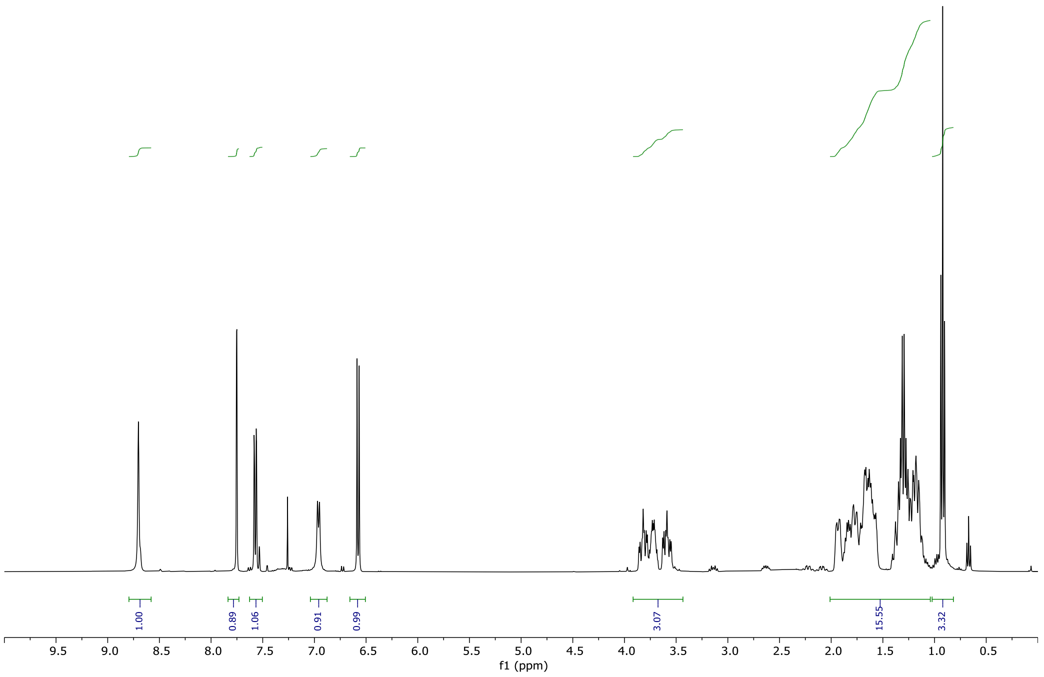
1.00
0.89
1.06
0.91
0.99
3.07
15.55
3.32
9.5 9.0 8.5 8.0 7.5 7.0 6.5 6.0 5.5 5.0 4.5 4.0 3.5 3.0 2.5 2.0 1.5 1.0 0.5
f1 (ppm)

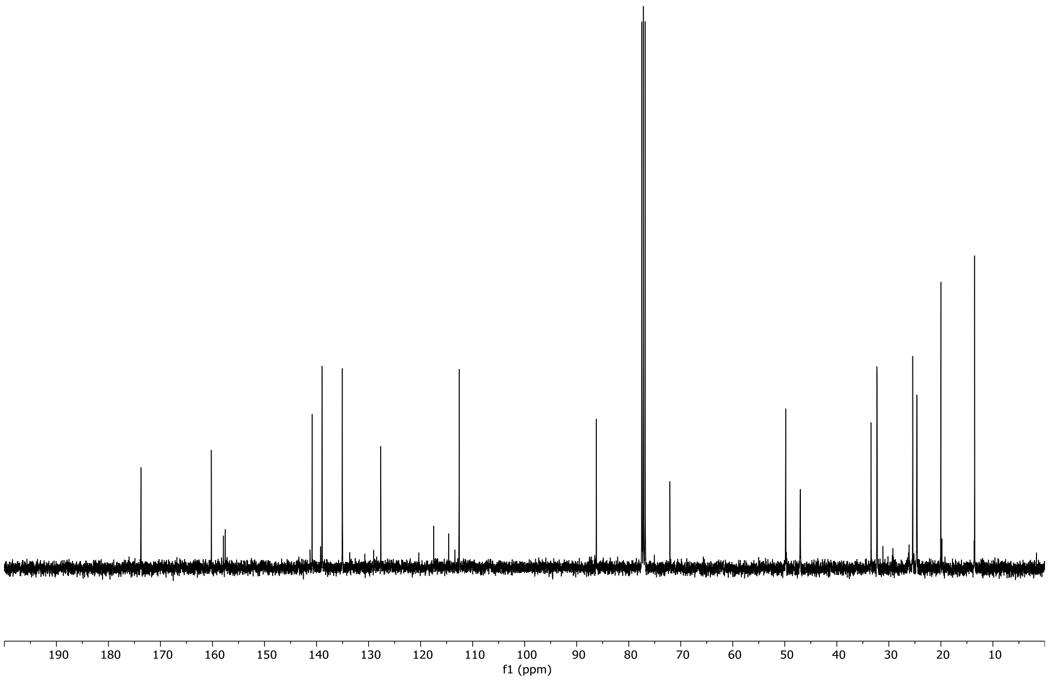
190 180 170 160 150 140 130 120 110 100 90 80 70 60 50 40 30 20 10
f1 (ppm)

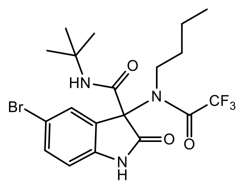
HN
O
N
CF3
Br
O
O
N
H

19

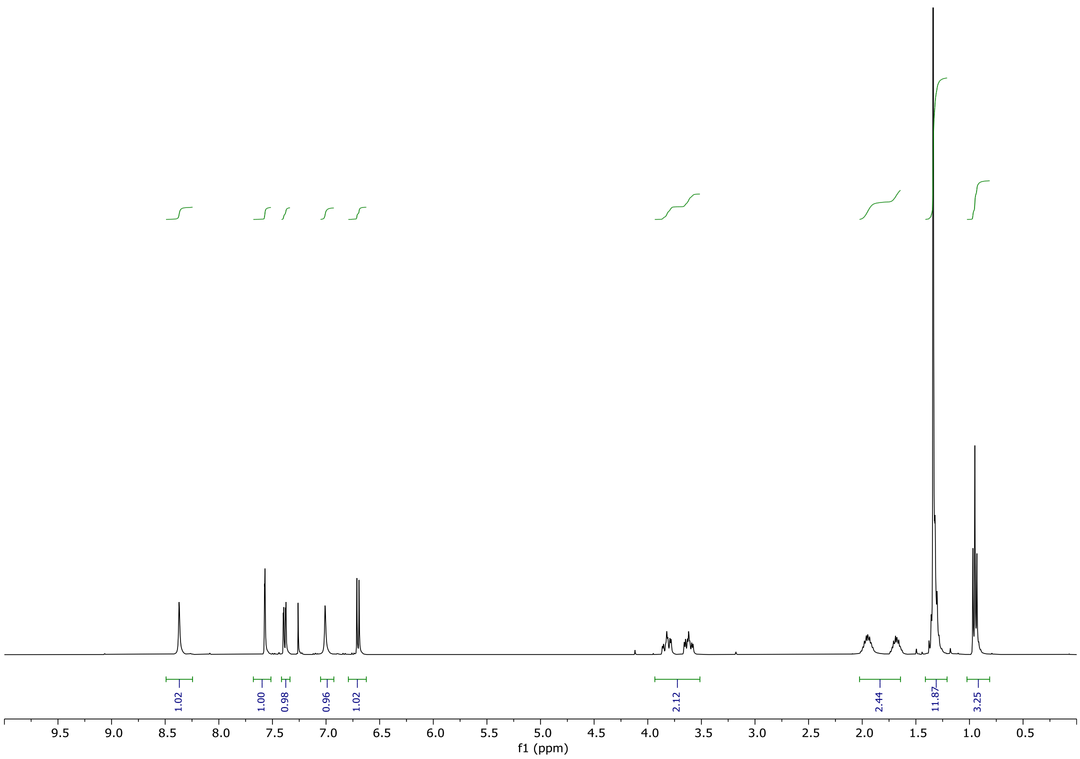
1.02
1.00
0.98
0.96
1.02
2.12
2.44
11.87
3.25
9.5 9.0 8.5 8.0 7.5 7.0 6.5 6.0 5.5 5.0 4.5 4.0 3.5 3.0 2.5 2.0 1.5 1.0 0.5
f1 (ppm)

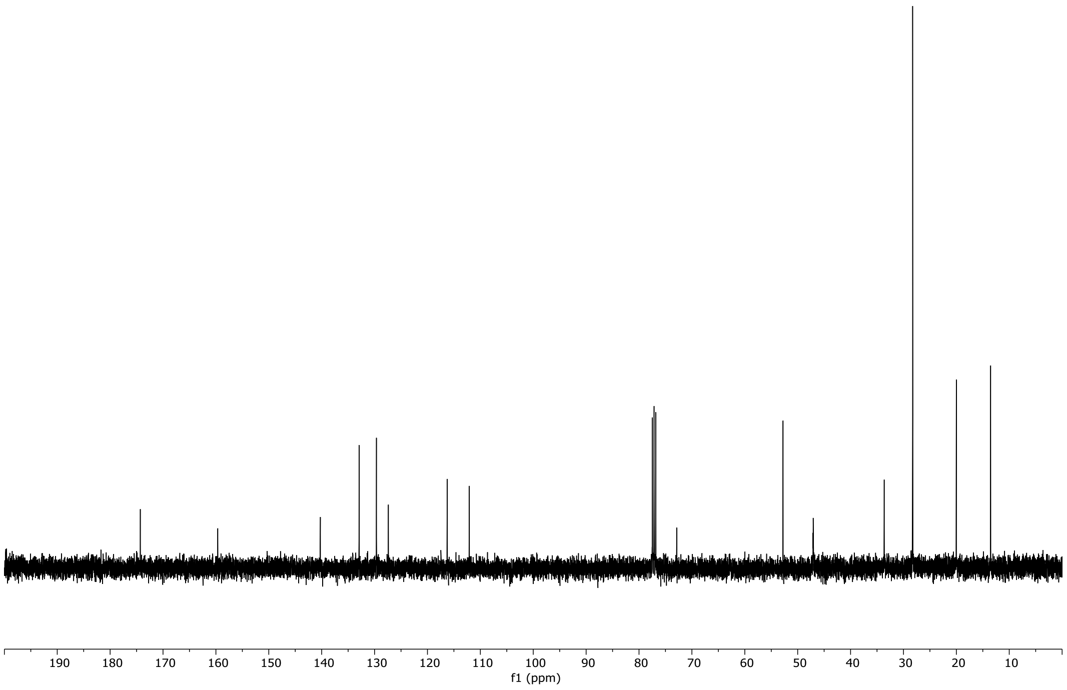
190 180 170 160 150 140 130 120 110 100 90 80 70 60 50 40 30 20 10
f1 (ppm)

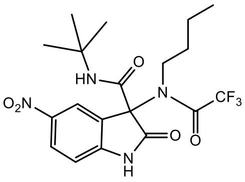
HN
O
N
CF_3
O_2N
O
O
N
H

20

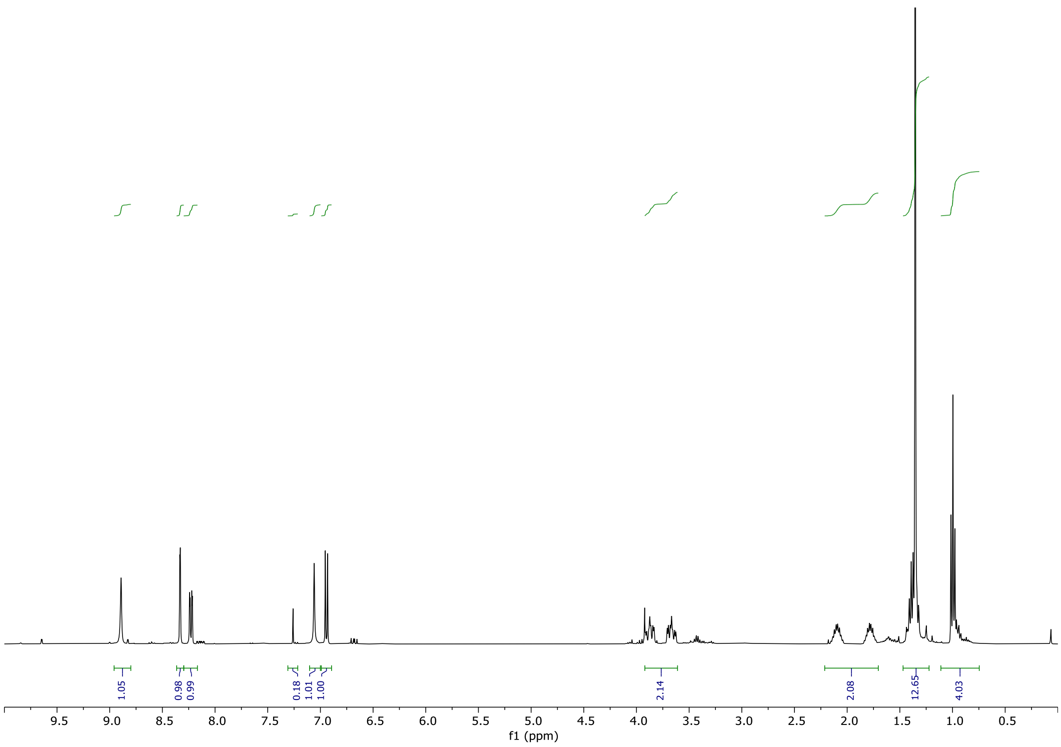
1.05
0.98
0.99
0.18
1.01
1.00
2.14
2.08
12.65
4.03
9.5 9.0 8.5 8.0 7.5 7.0 6.5 6.0 5.5 5.0 4.5 4.0 3.5 3.0 2.5 2.0 1.5 1.0 0.5
f1 (ppm)

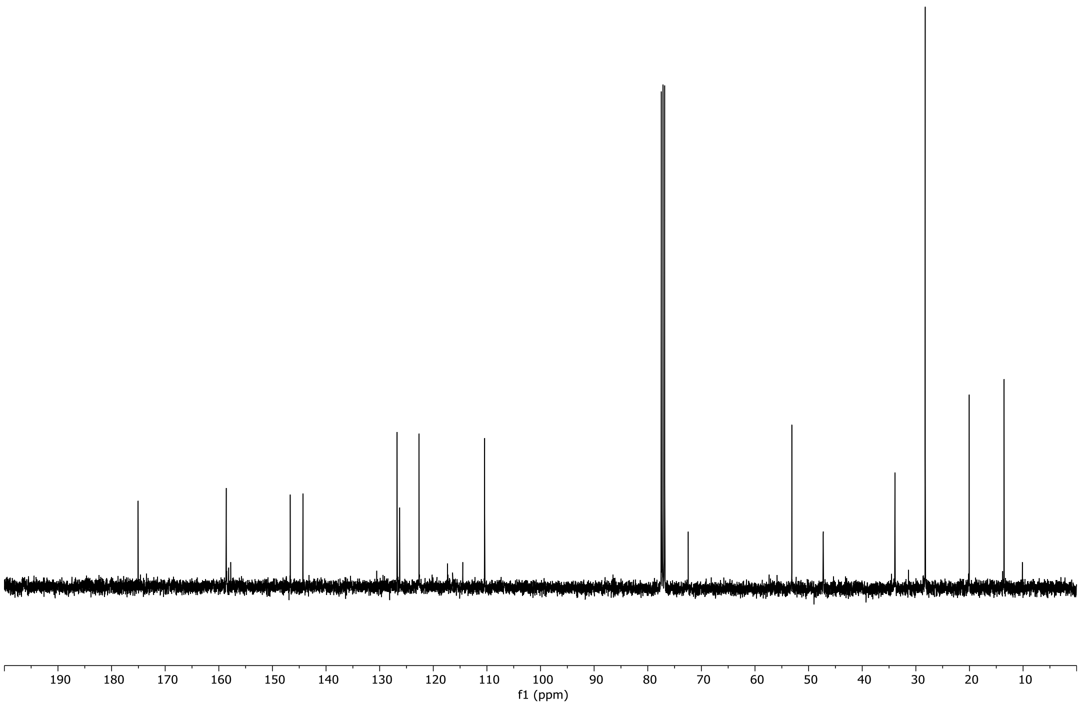
190 180 170 160 150 140 130 120 110 100 90 80 70 60 50 40 30 20 10
f1 (ppm)

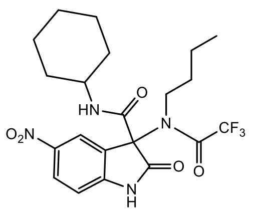
HN
O
N
CF3
O2N
O
O
N
H

21

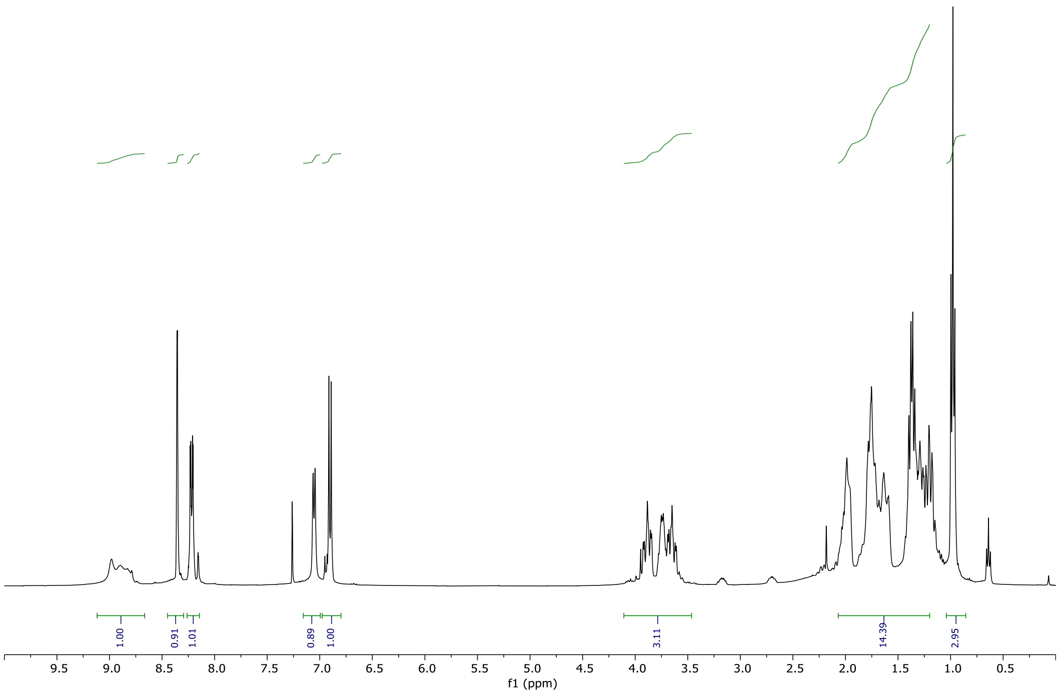
1.00
0.91
1.01
0.89
1.00
3.11
14.39
2.95
9.5 9.0 8.5 8.0 7.5 7.0 6.5 6.0 5.5 5.0 4.5 4.0 3.5 3.0 2.5 2.0 1.5 1.0 0.5
f1 (ppm)

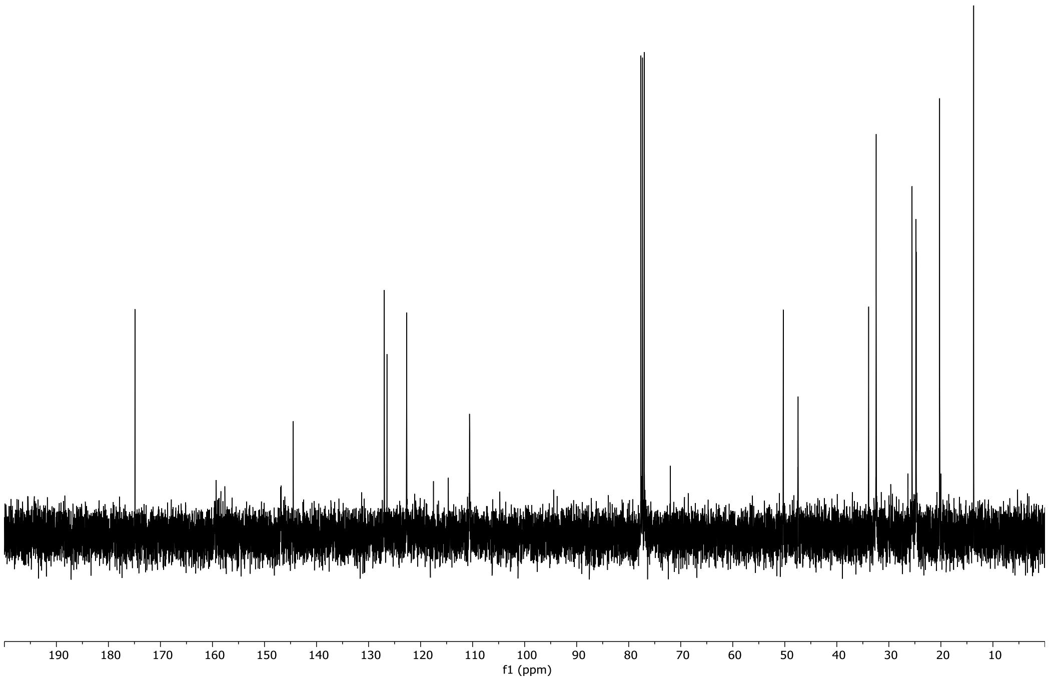
190 180 170 160 150 140 130 120 110 100 90 80 70 60 50 40 30 20 10
f1 (ppm)

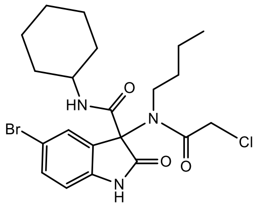
HN
O
N
Br
O
Cl
O
N
H

22

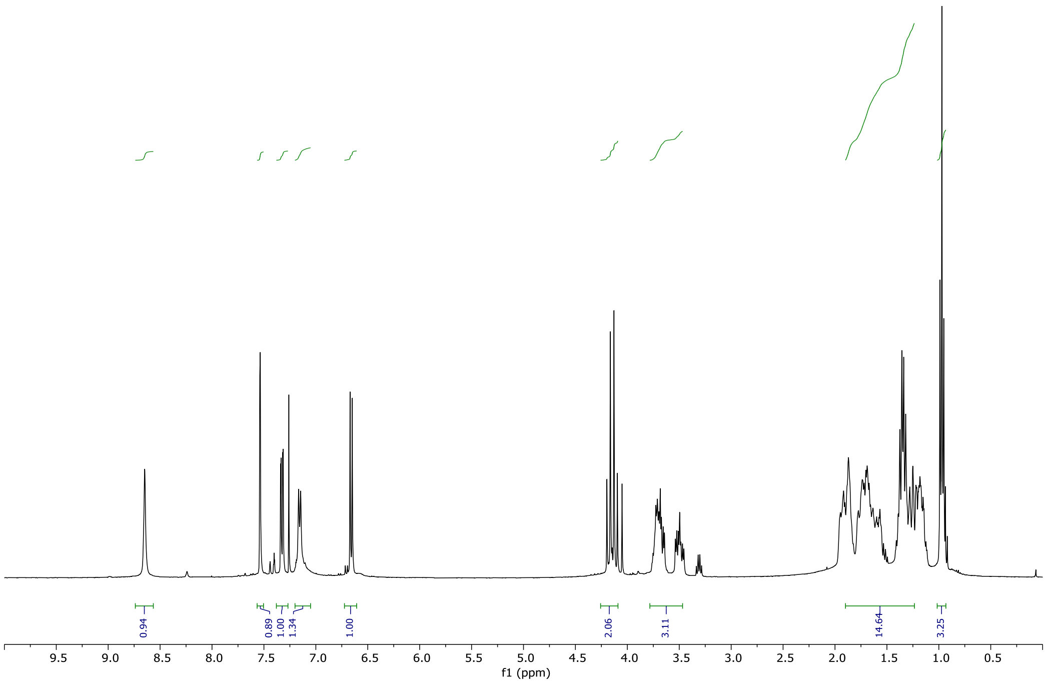
0.94
0.89
1.00
1.34
1.00
2.06
3.11
14.64
3.25
9.5 9.0 8.5 8.0 7.5 7.0 6.5 6.0 5.5 5.0 4.5 4.0 3.5 3.0 2.5 2.0 1.5 1.0 0.5
f1 (ppm)

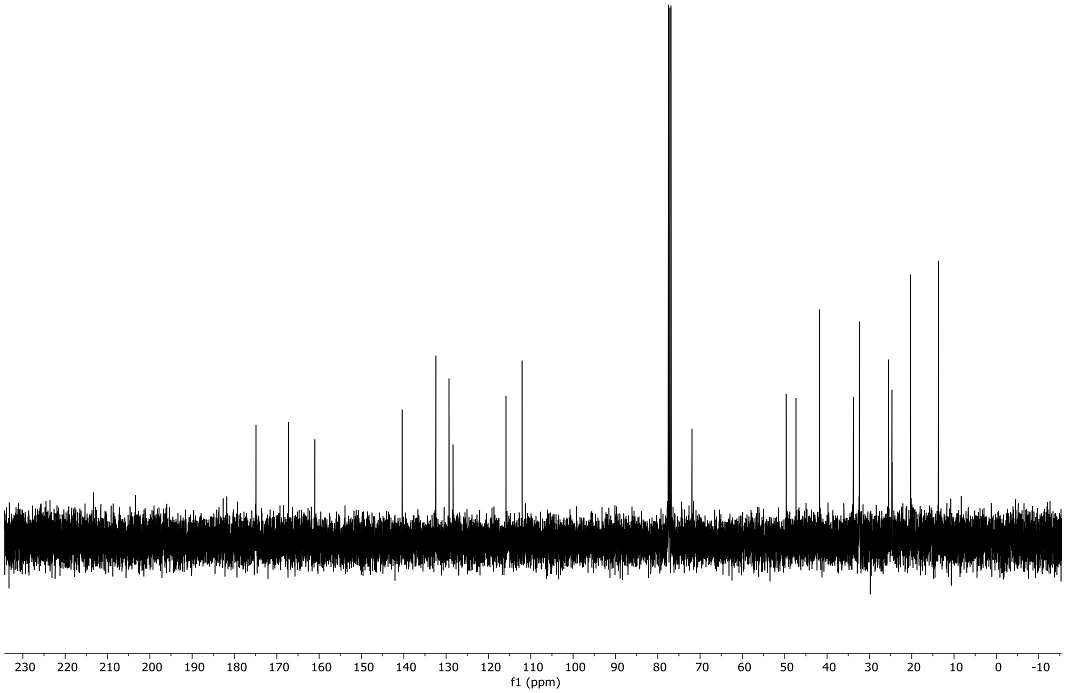
230 220 210 200 190 180 170 160 150 140 130 120 110 100 90 80 70 60 50 40 30 20 10 0 -10
f1 (ppm)

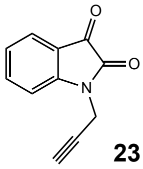

23

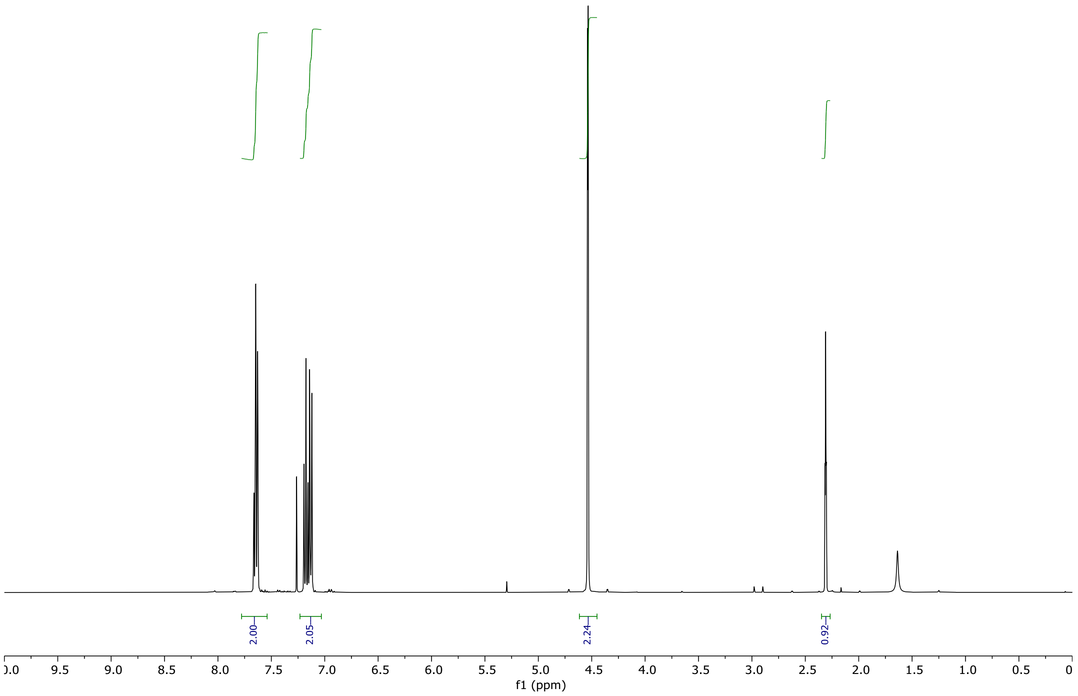

2.00
2.05
2.24
0.92
9.5
9.0
8.5
8.0
7.5
7.0
6.5
6.0
5.5
5.0
4.5
4.0
3.5
3.0
2.5
2.0
1.5
1.0
0.5
0
f1 (ppm)

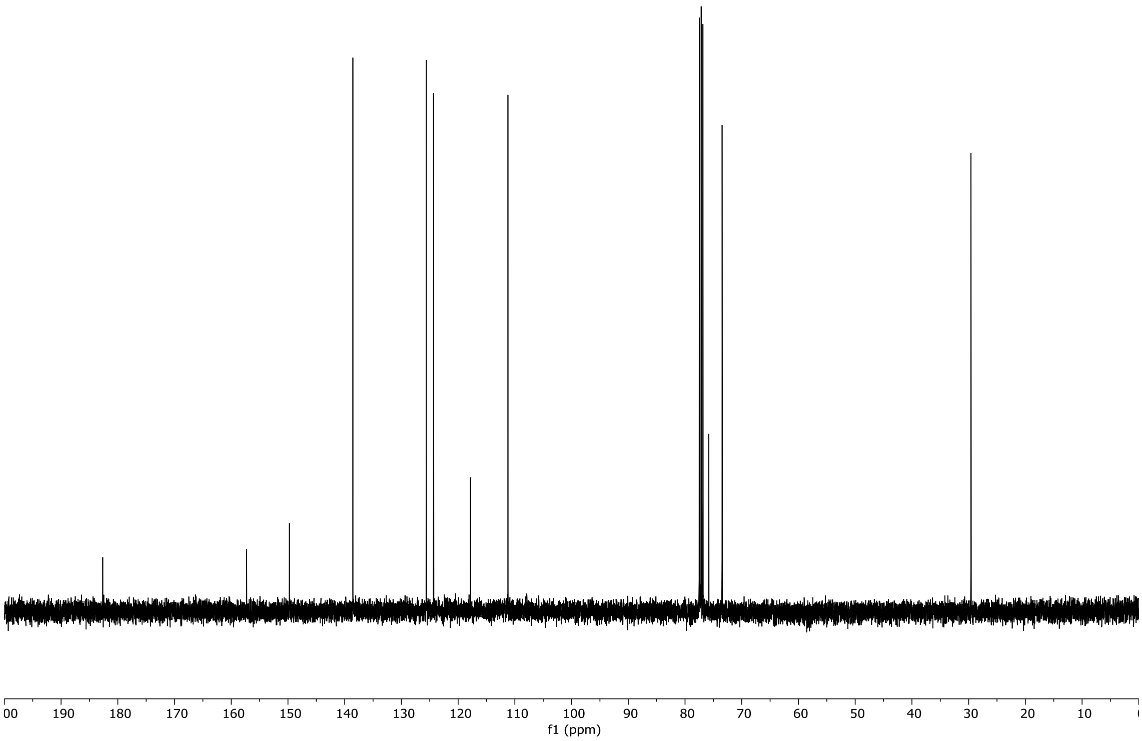

190
180
170
160
150
140
130
120
110
100
90
80
70
60
50
40
30
20
10
f1 (ppm)

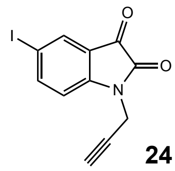

24

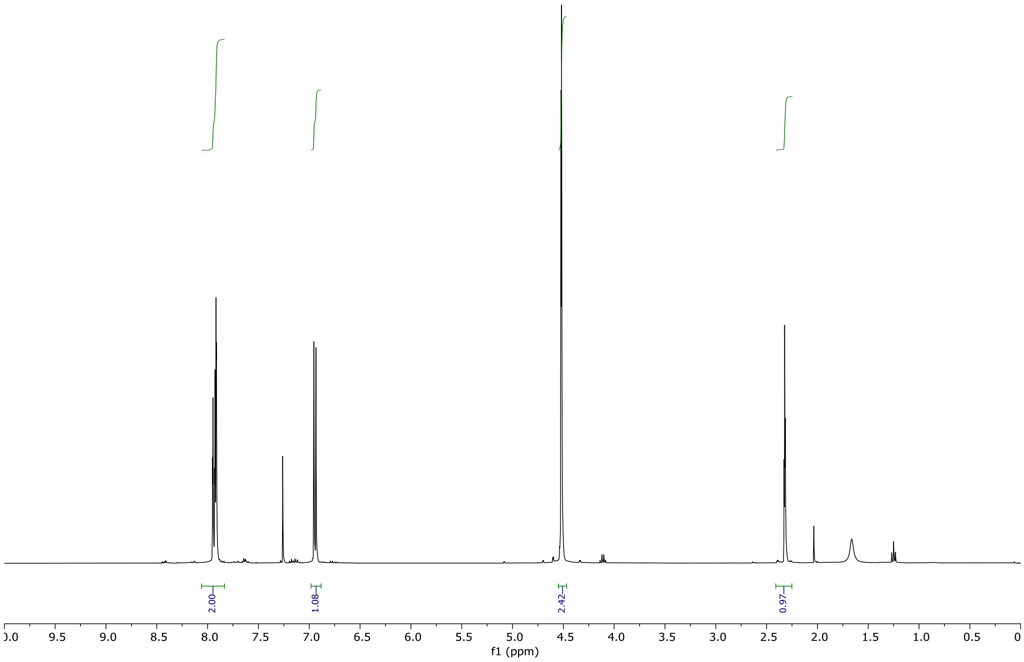

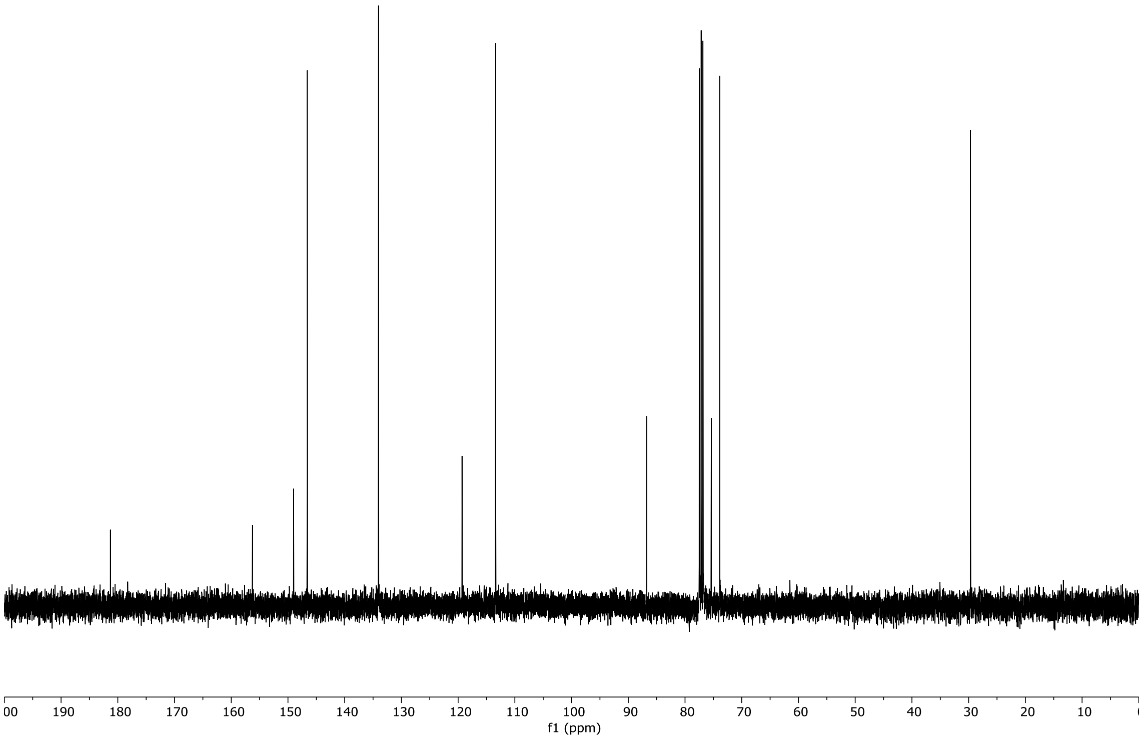

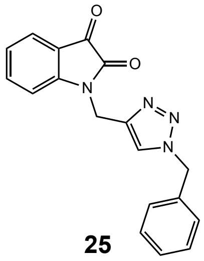

25

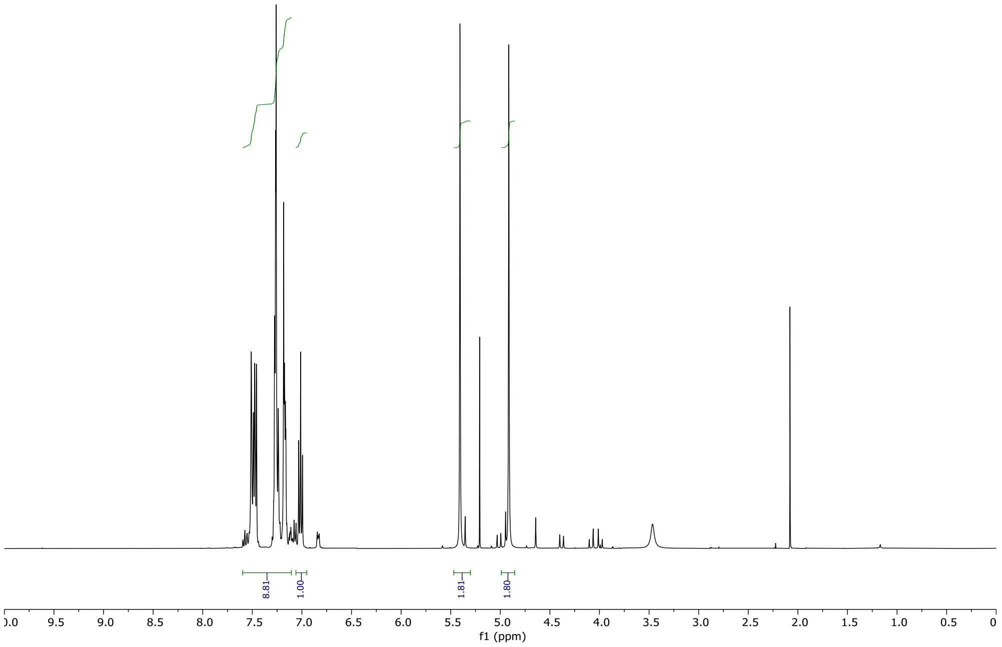

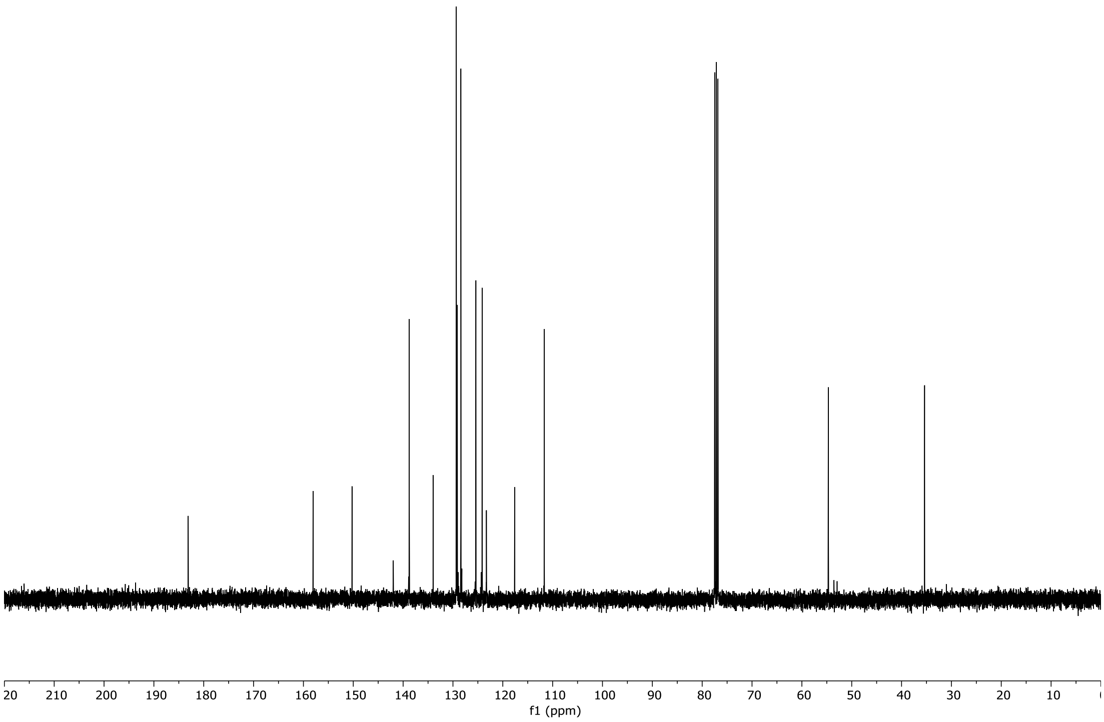

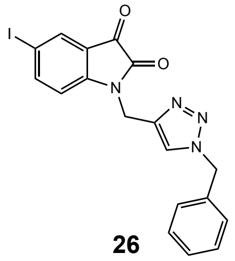

26

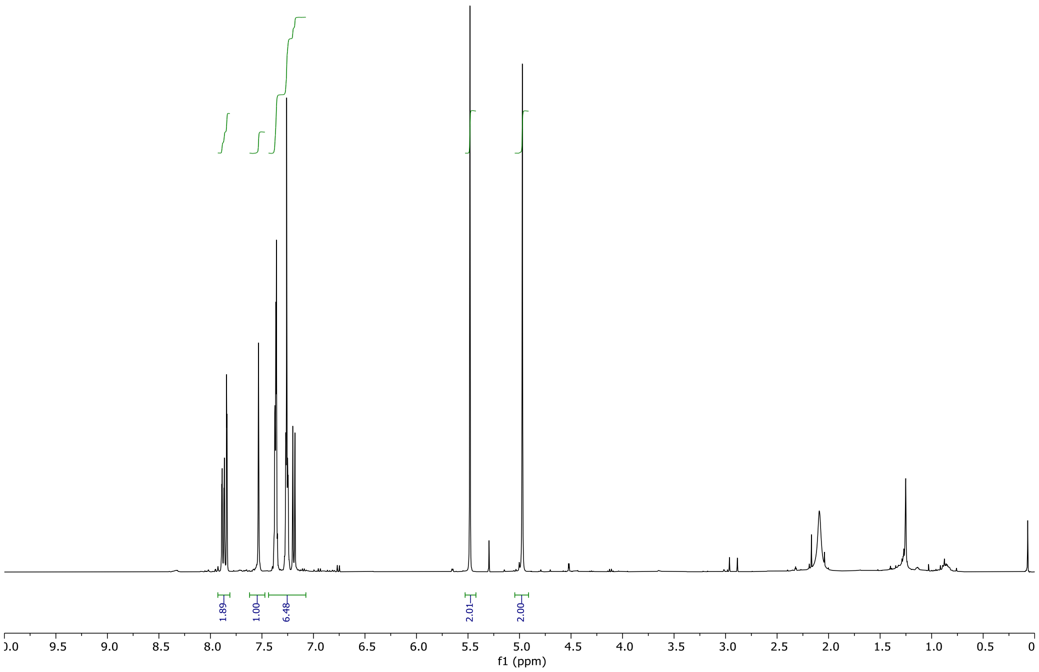

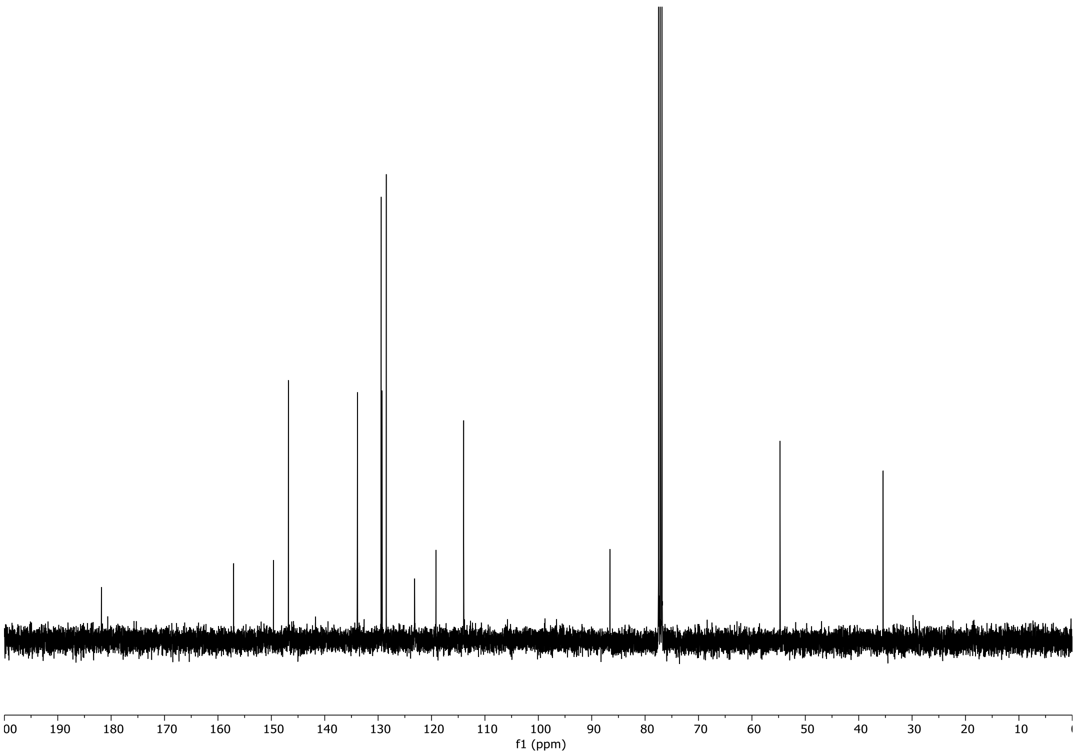

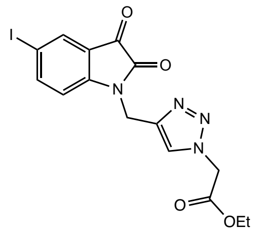

27

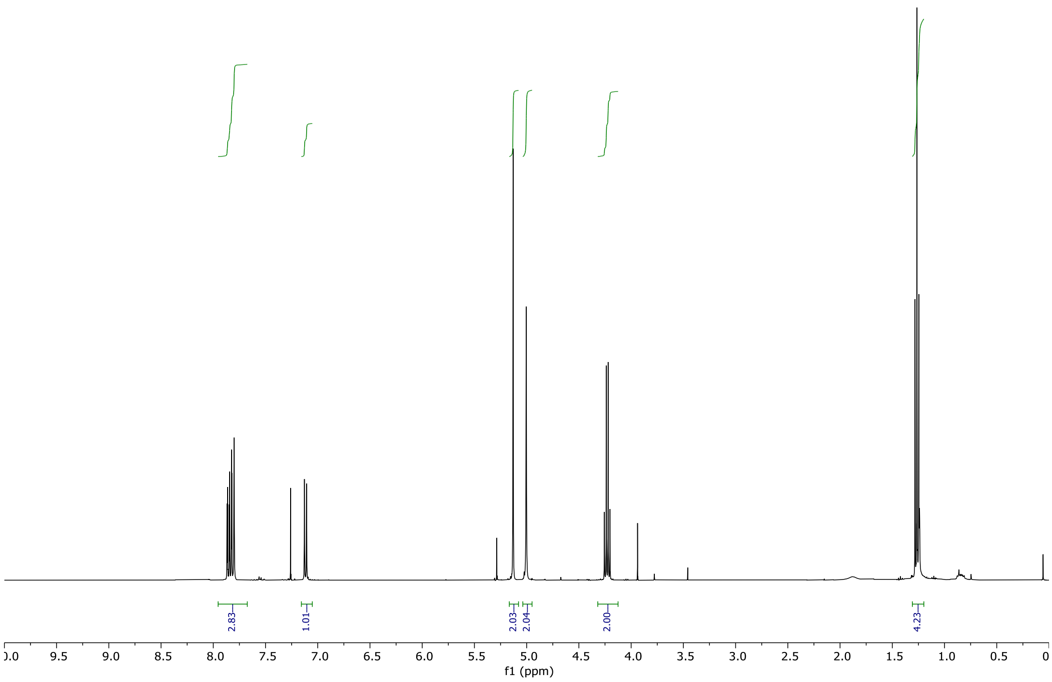

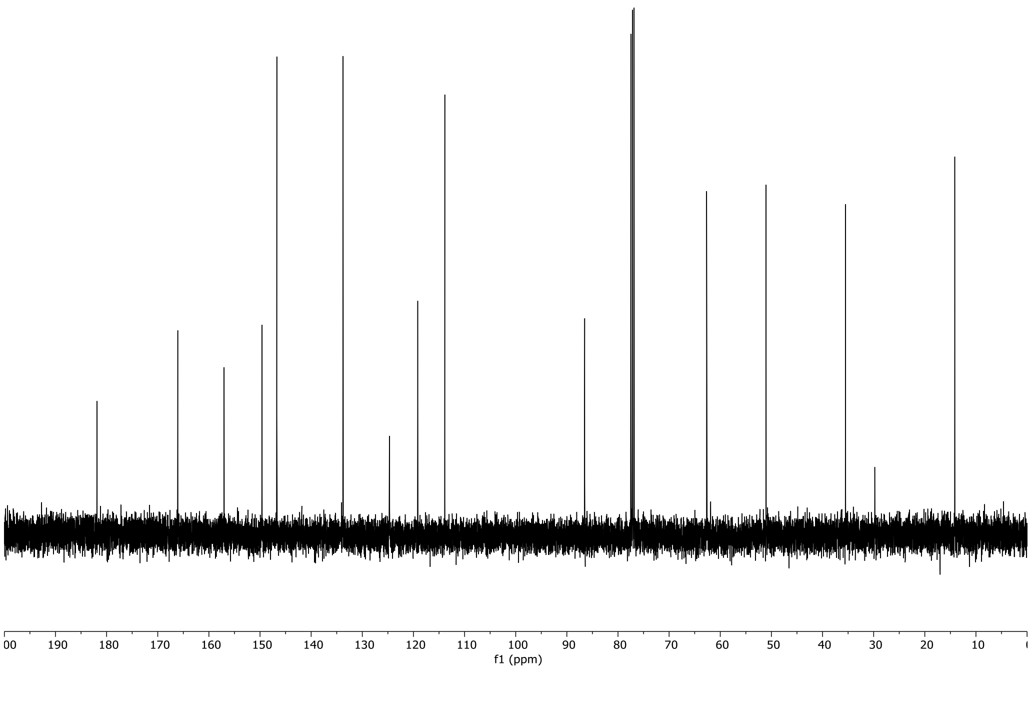

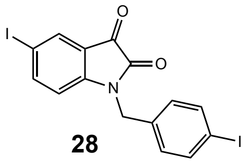

28

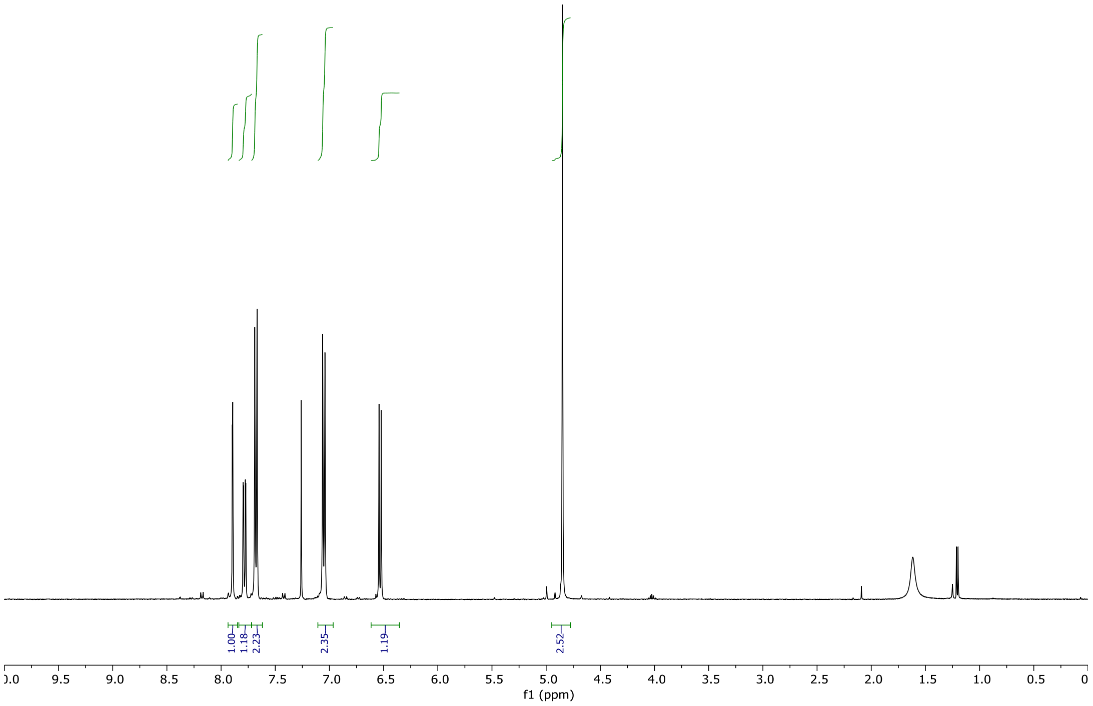

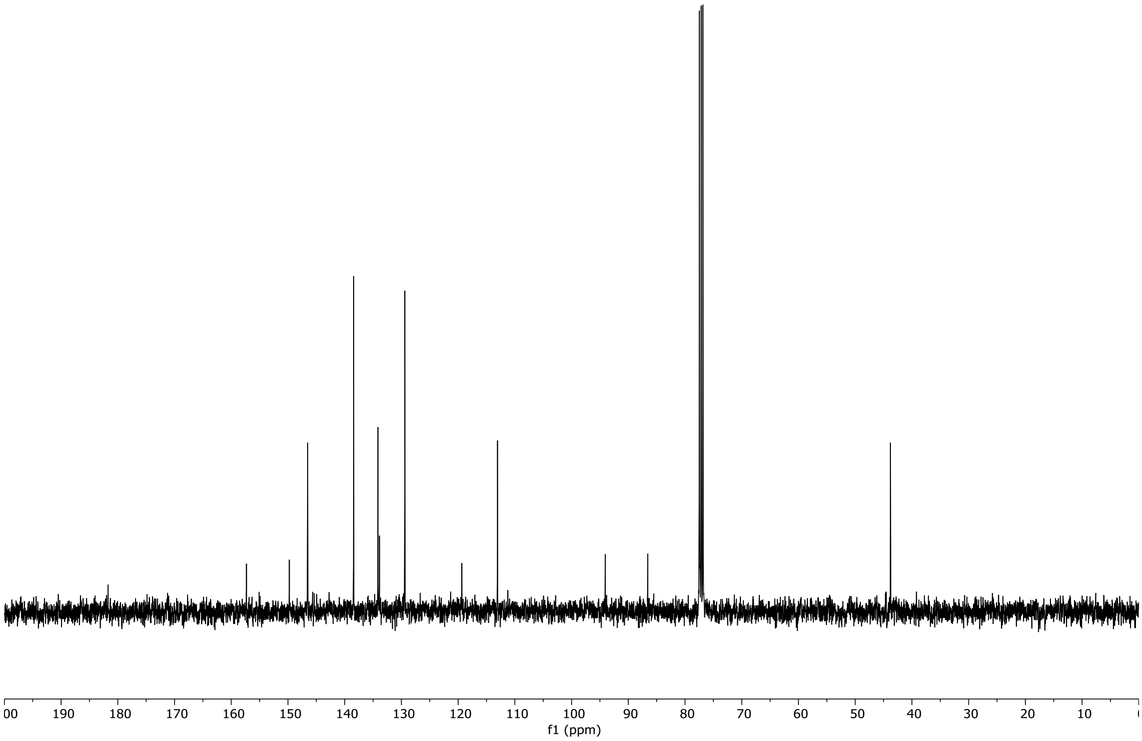

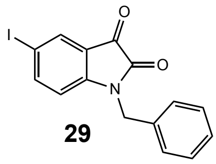

29

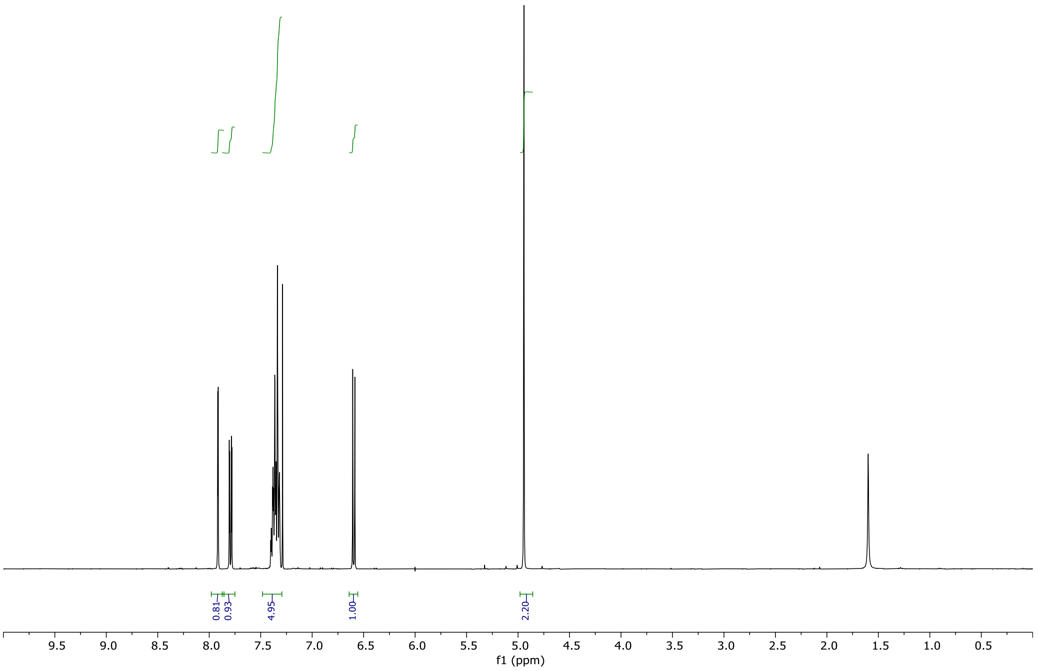
0.81
0.93
4.95
1.00
2.20
9.5 9.0 8.5 8.0 7.5 7.0 6.5 6.0 5.5 5.0 4.5 4.0 3.5 3.0 2.5 2.0 1.5 1.0 0.5
f1 (ppm)

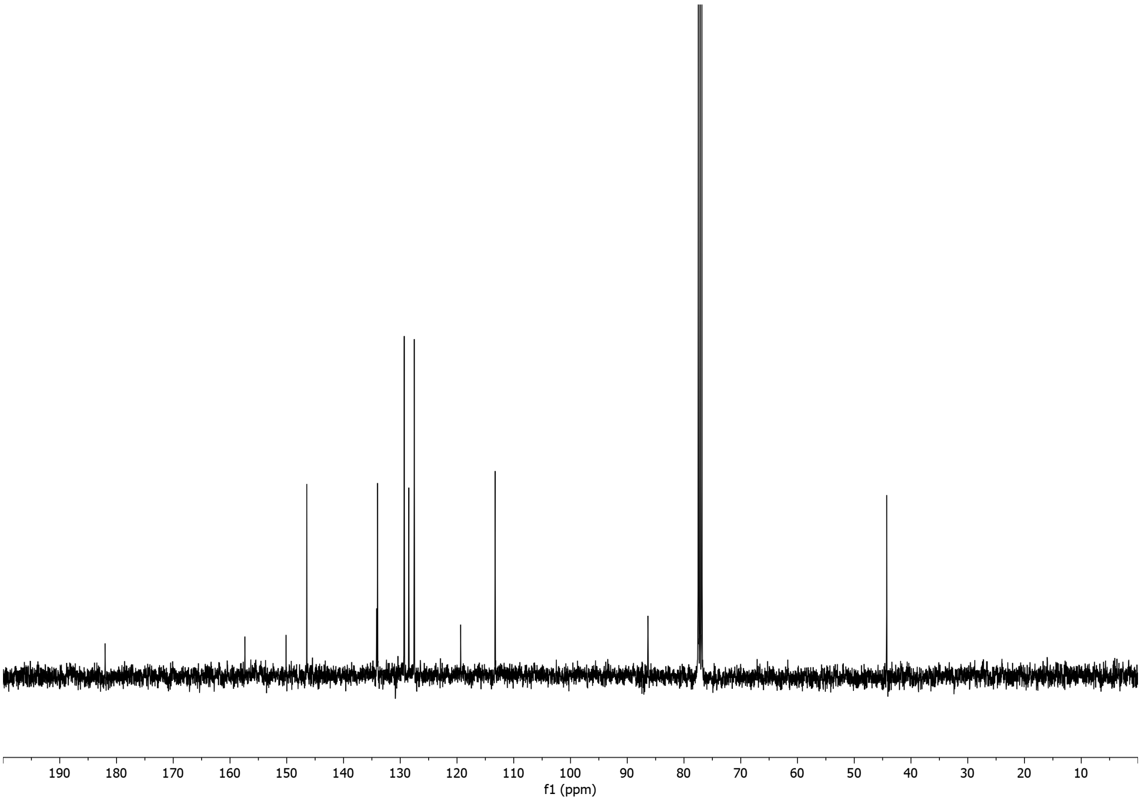
190 180 170 160 150 140 130 120 110 100 90 80 70 60 50 40 30 20 10
f1 (ppm)

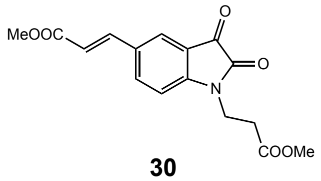

30

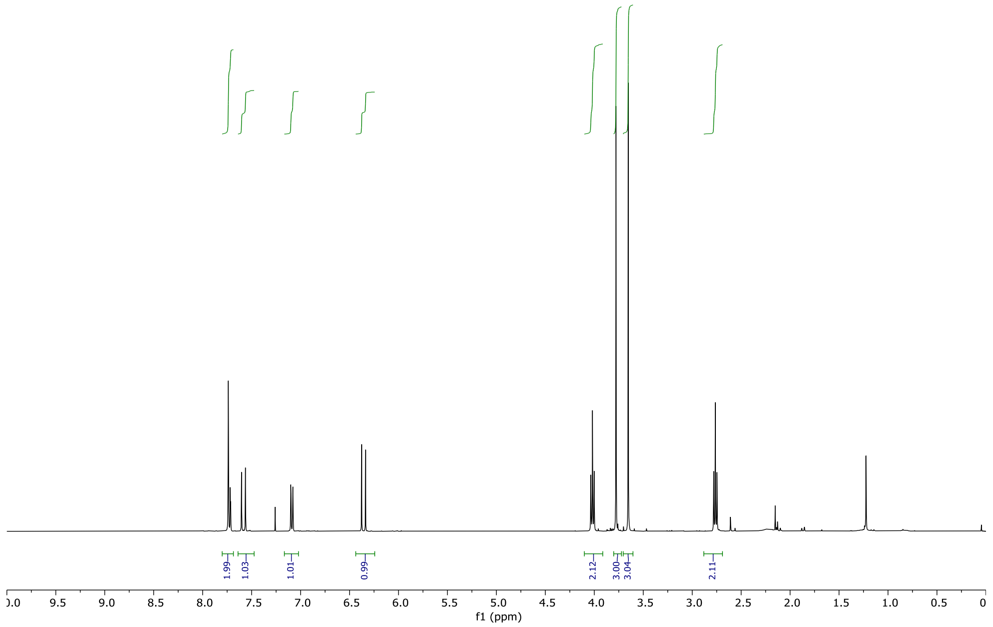

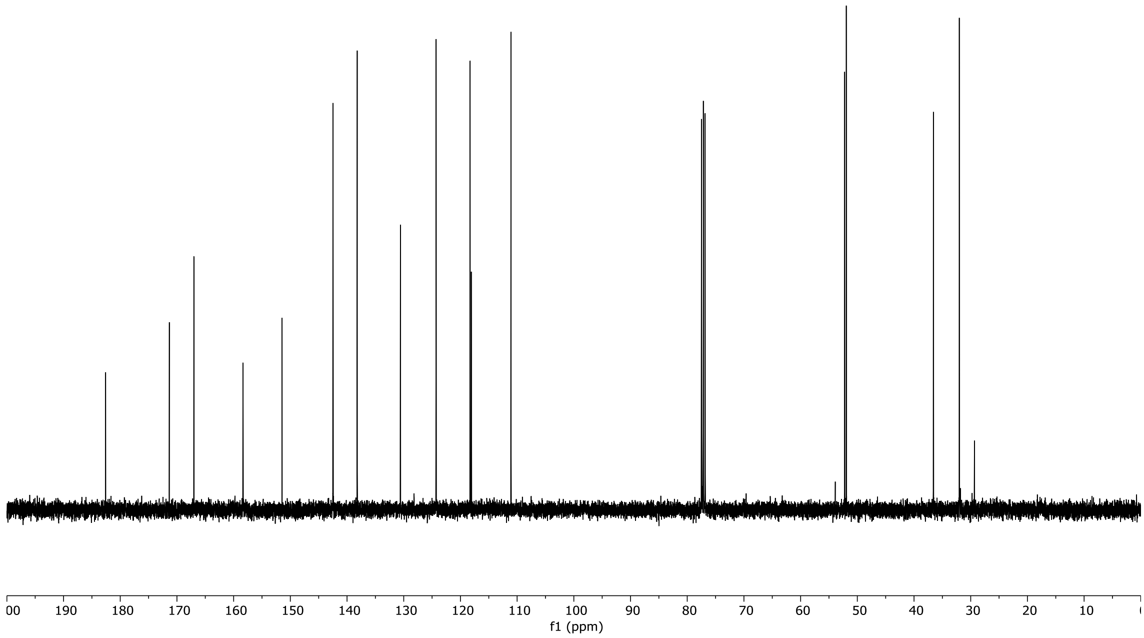

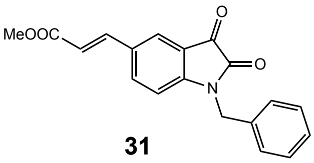
MeOOC
O
O
N

31

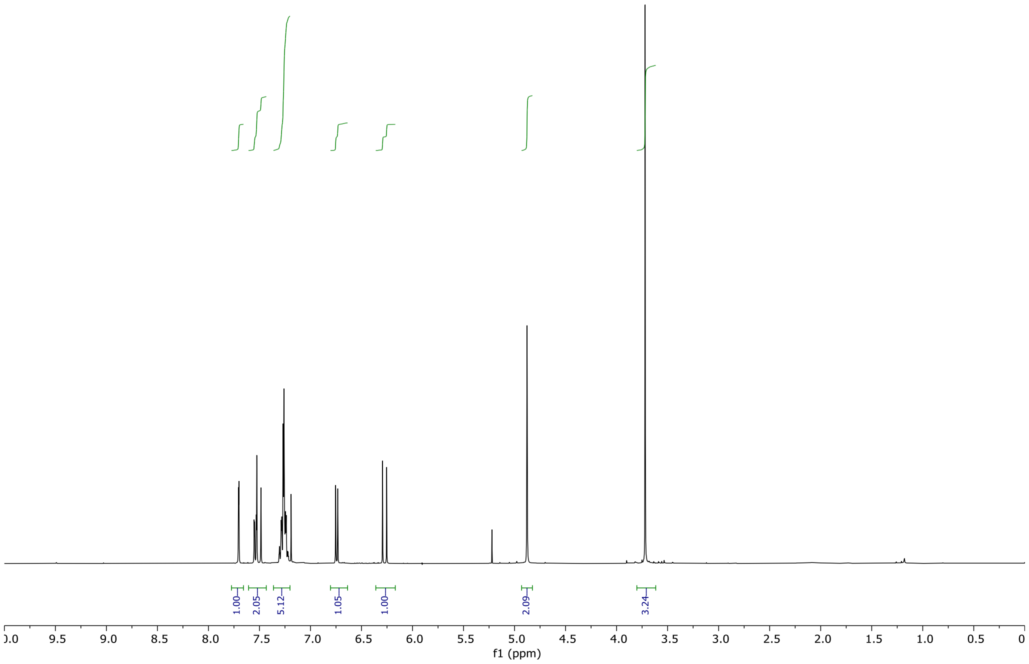
1.00
2.05
5.12
1.05
1.00
2.09
3.24
9.5 9.0 8.5 8.0 7.5 7.0 6.5 6.0 5.5 5.0 4.5 4.0 3.5 3.0 2.5 2.0 1.5 1.0 0.5 0
f1 (ppm)

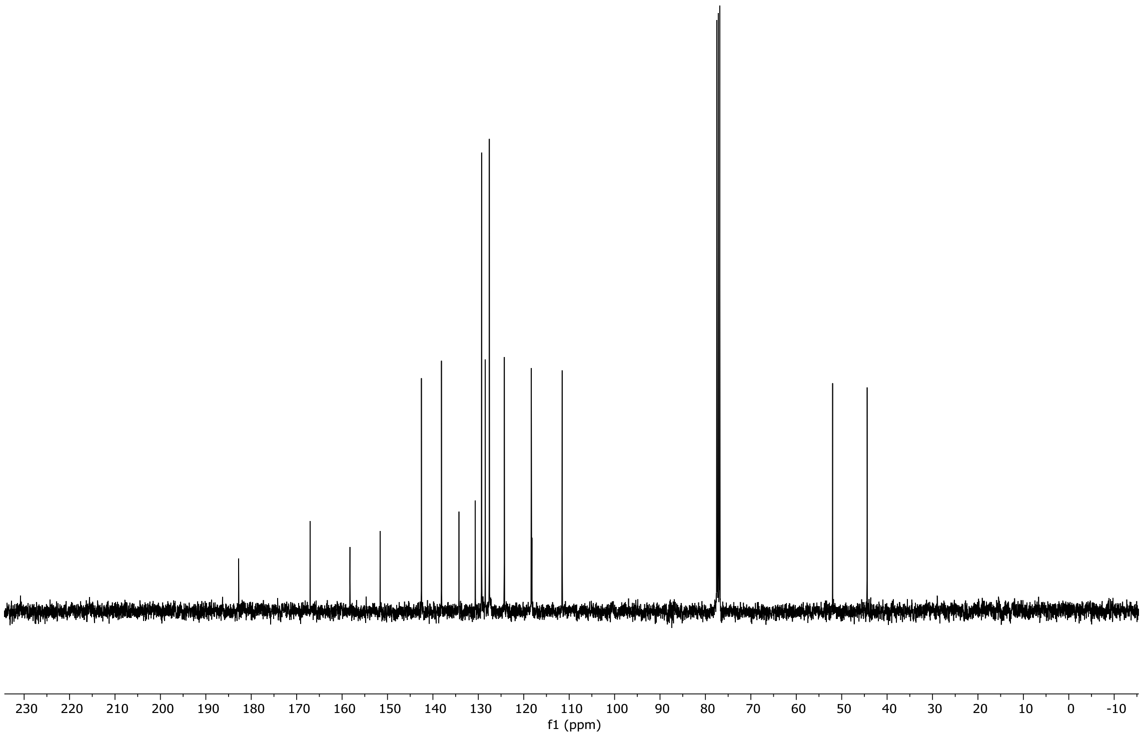
230 220 210 200 190 180 170 160 150 140 130 120 110 100 90 80 70 60 50 40 30 20 10 0 -10
f1 (ppm)

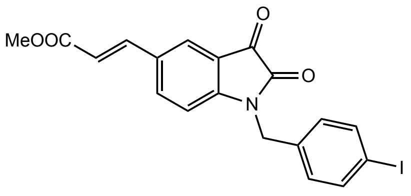

32

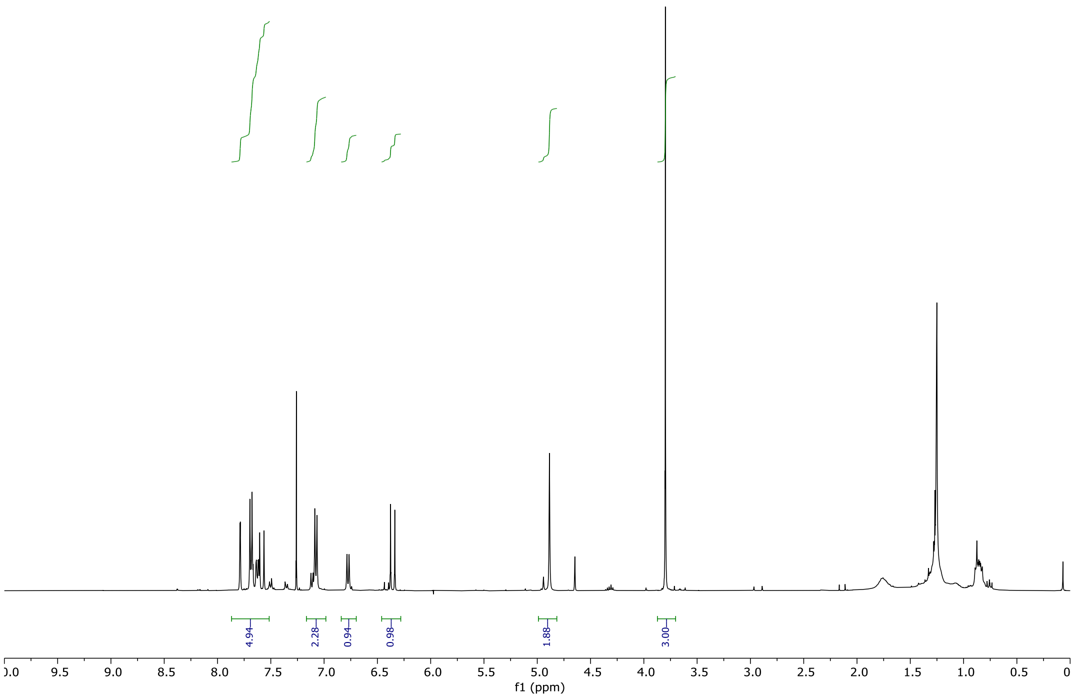

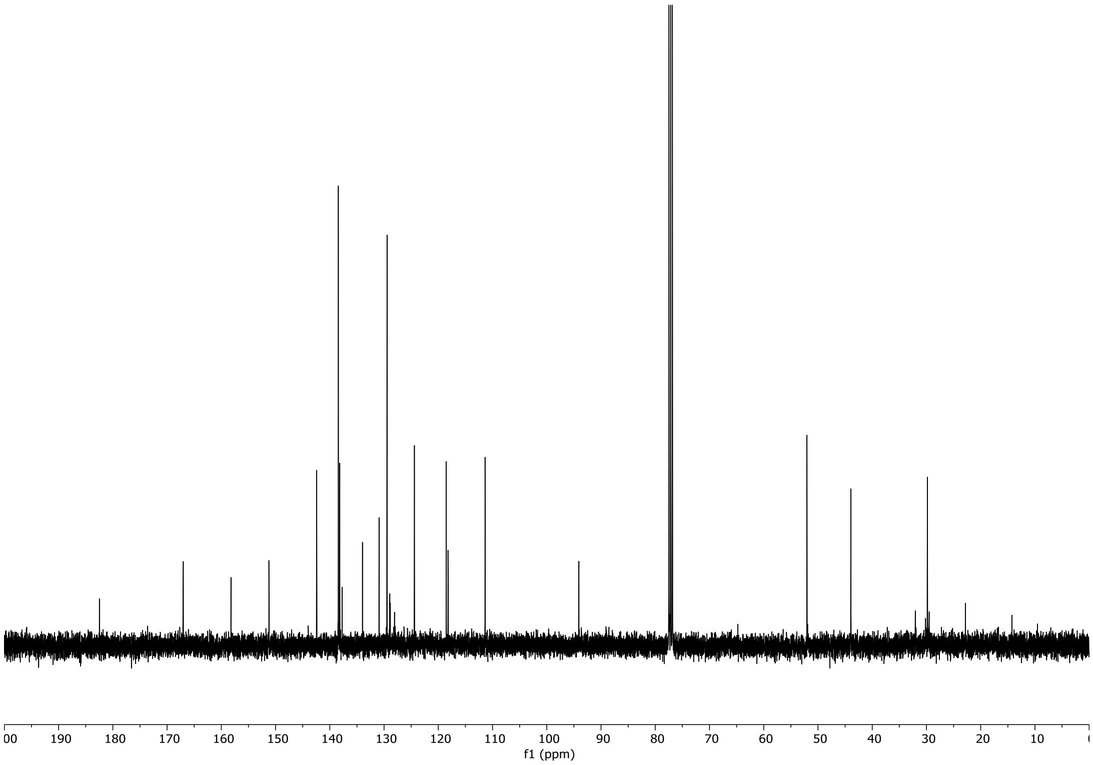

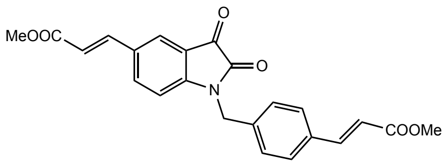

33

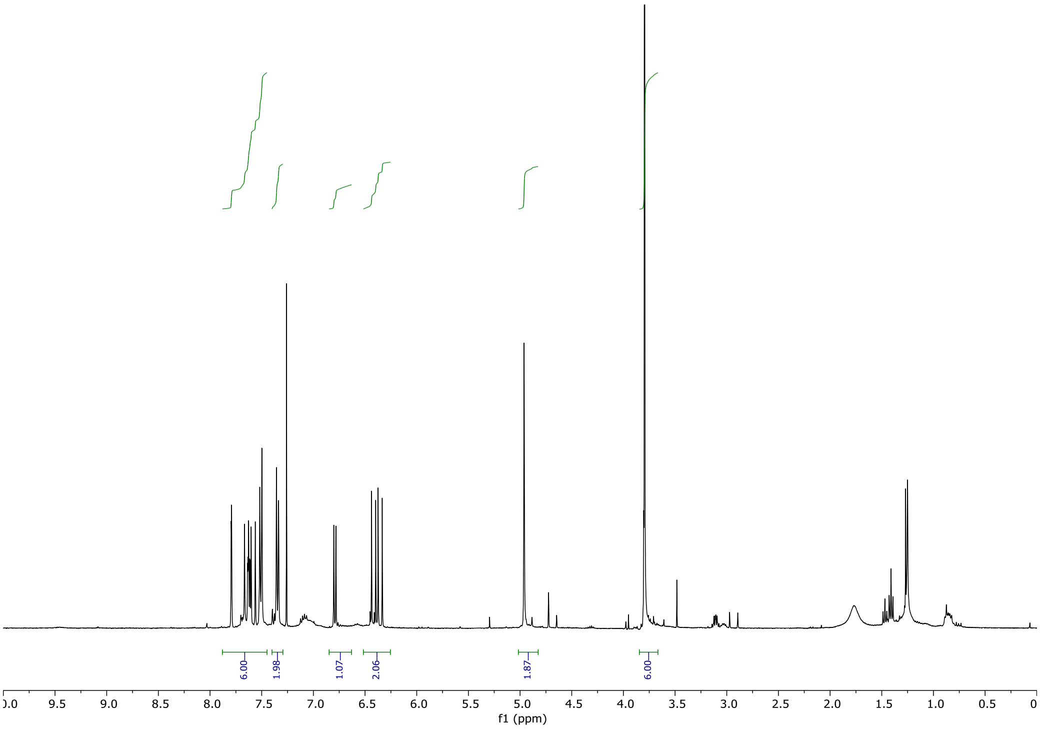

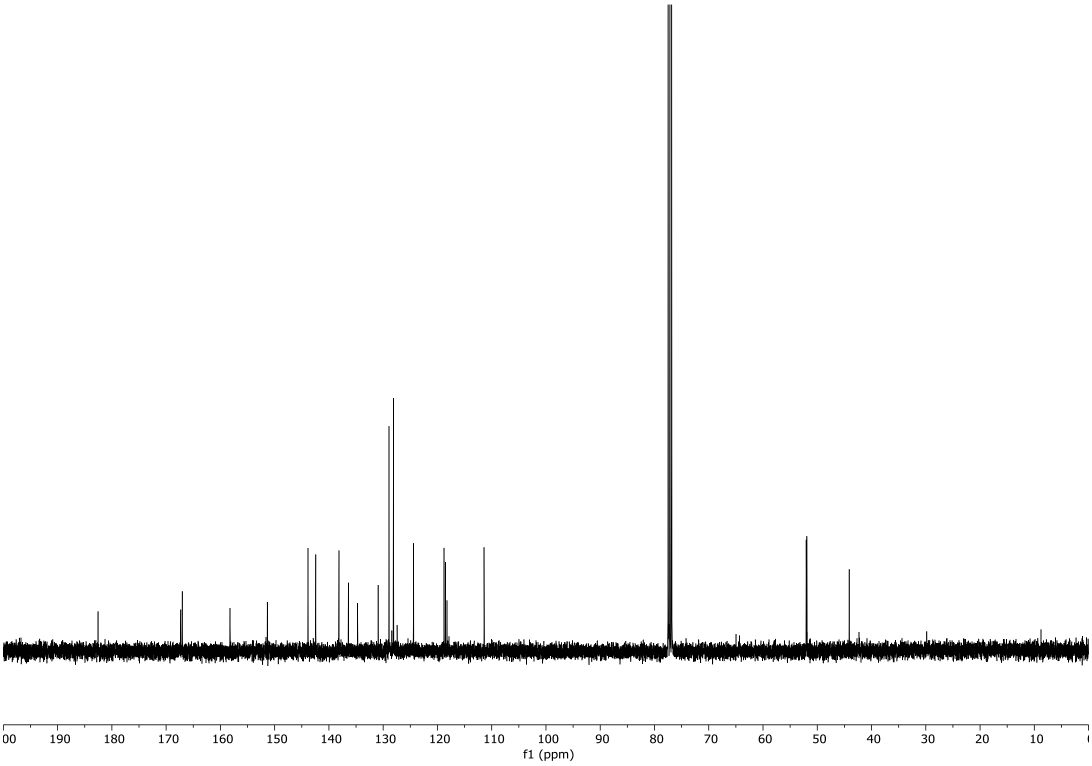

4. ESI-masas spectra of the related compounds

AREA DE INFRAESTRUCTURAS DE INVESTIGACION
UNIDADE DE ESPECTROMETRIA DE MASAS E PROTEÓMICA

Edificio CACTUS * Campus Vida
15782 Santiago de Compostela
Tel. 881816242. Ext. 16242
E-mail: sxemasas@usc.es
Web: https://www.usc.es/gl/investigacion/riaidt/espectrometria/

ESPECTRO ESI-FIA-TOF

Analysis Info

Sample Name	4 ESI MF3-CM-96 447_42_01_803.d	Acquisition Date	04/06/2022 11:18:52
Method	803.m **COMPOUND 14**	Instrument	amaZon ETD

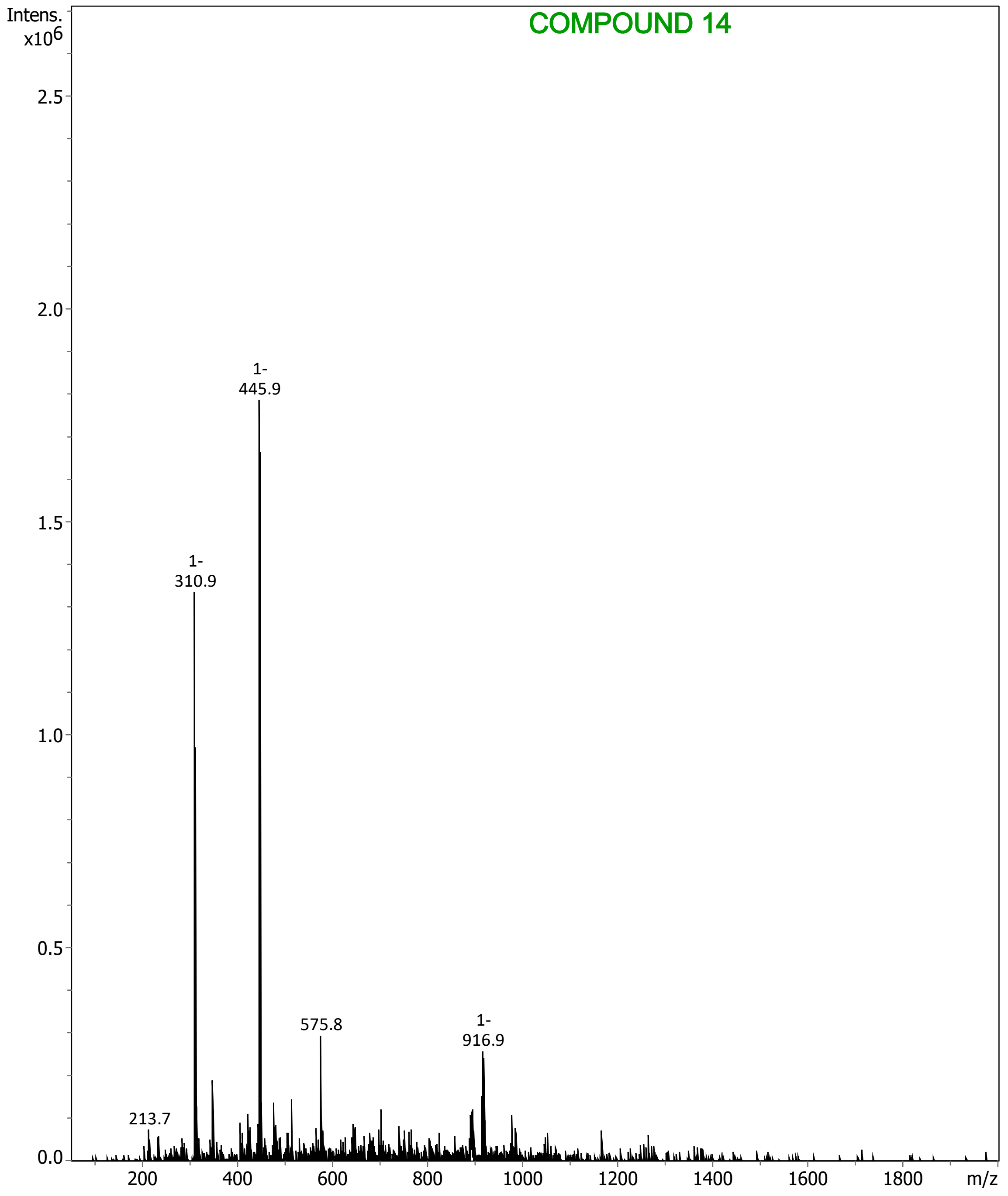

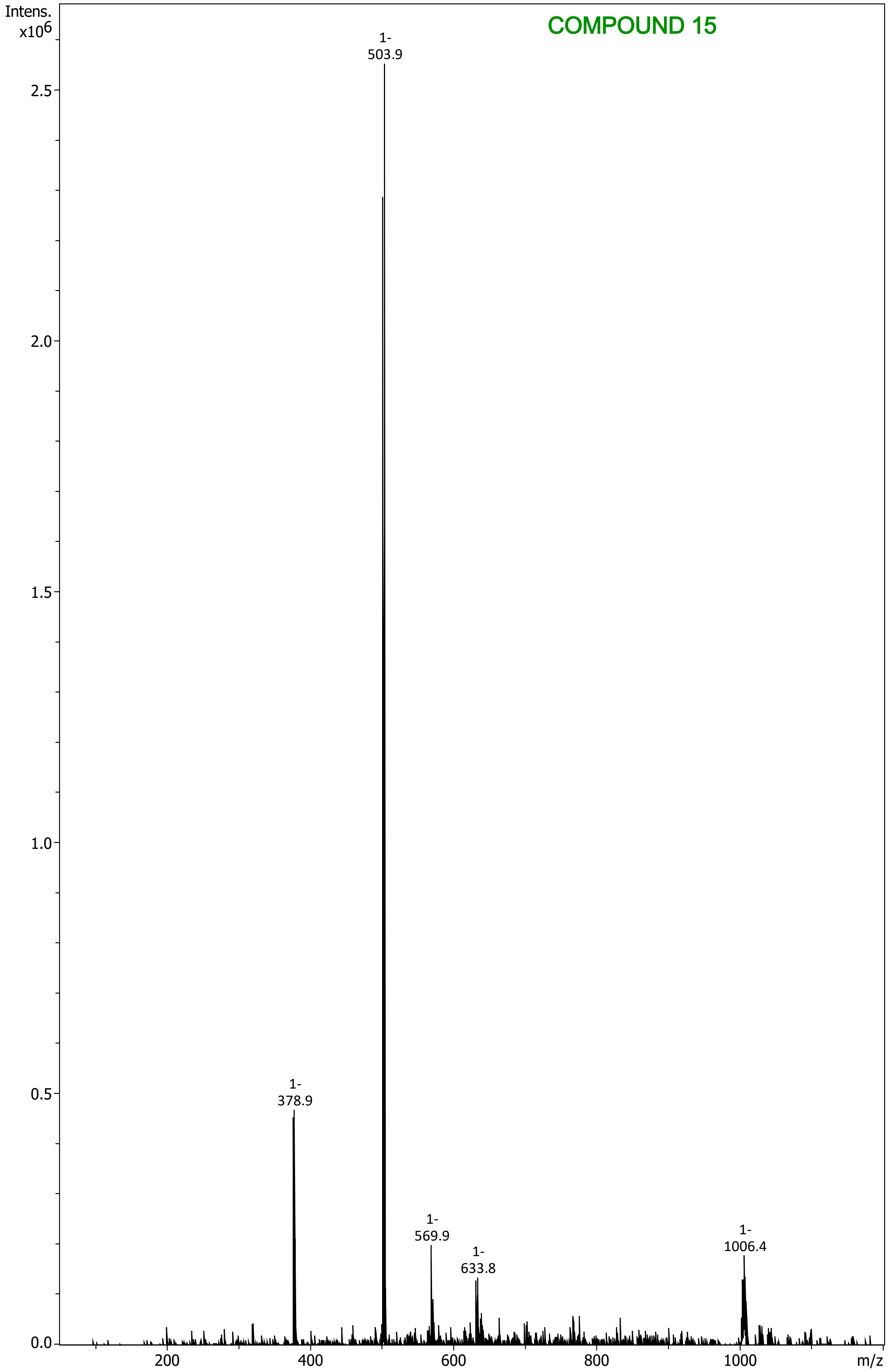

Intens.
x10^6
COMPOUND 15
1-
503.9
1-
378.9
1-
569.9
1-
633.8
1-
1006.4
2.5
2.0
1.5
1.0
0.5
0.0
200
400
600
800
1000
m/z

AREA DE INFRAESTRUCTURAS DE INVESTIGACION
UNIDADE DE ESPECTROMETRIA DE MASAS E PROTEÓMICA

Edificio CACTUS * Campus Vida
15782 Santiago de Compostela
Tel. 881816242. Ext. 16242
E-mail: sxemasas@usc.es
Web: https://www.usc.es/gl/investigacion/riaidt/espectrometria/

ESPECTRO ESI-FIA-Ion Trap

Analysis Info

Sample Name	16 ESI MF-CM-117 457_51_01_824.d	Acquisition Date	04/18/2022 16:36:47
Method	824.m COMPOUND 16	Instrument	amaZon ETD

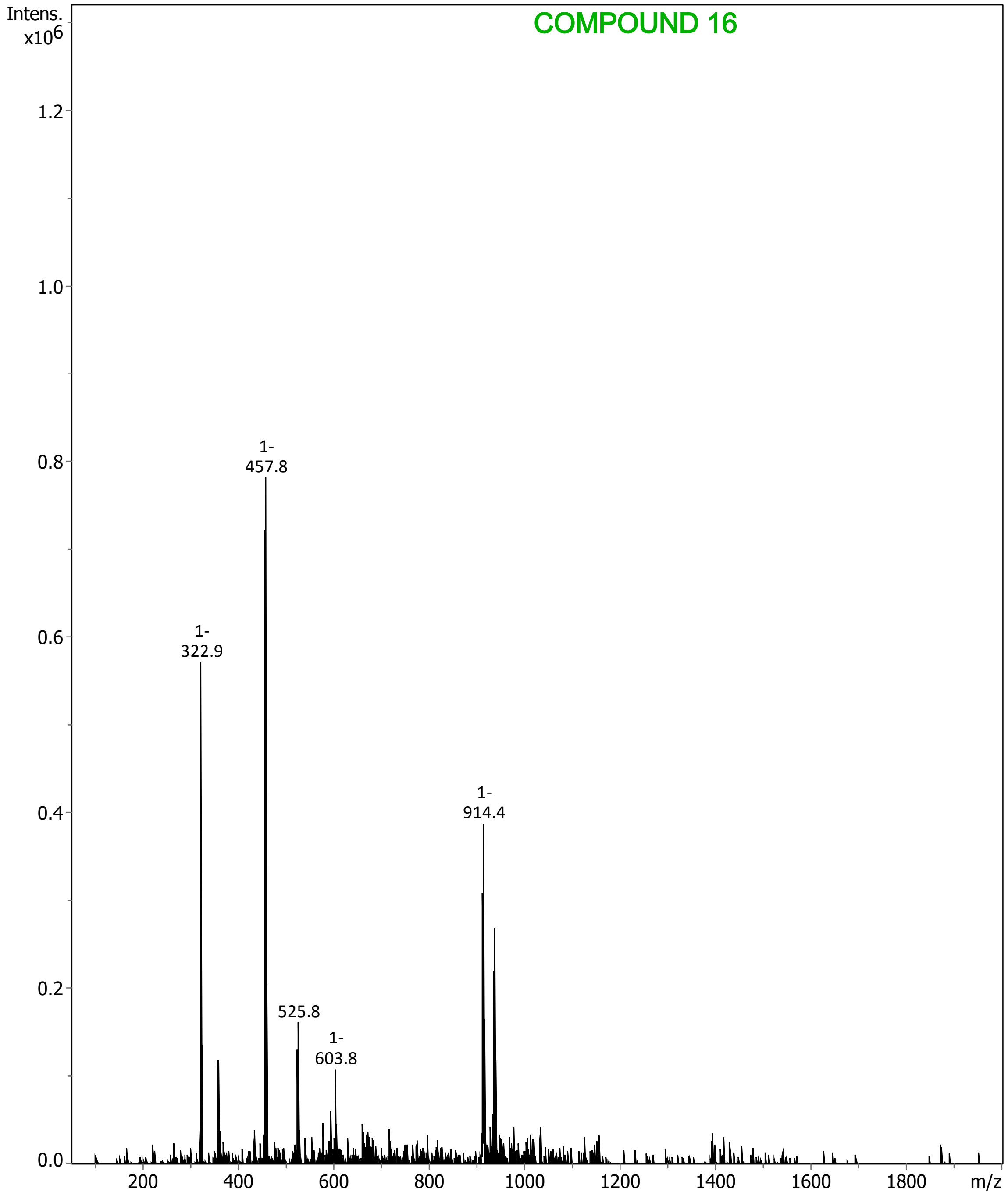

AREA DE INFRAESTRUCTURAS DE INVESTIGACION
UNIDADE DE ESPECTROMETRIA DE MASAS E PROTEÓMICA

Edificio CACTUS * Campus Vida
15782 Santiago de Compostela
Tel. 881816242. Ext. 16242
E-mail: sxemasas@usc.es
Web: https://www.usc.es/gl/investigacion/riaidt/espectrometria/

ESPECTRO ESI-FIA-Ion Trap

Analysis Info

Sample Name	5 ESI MF3-CM-146 525_45_01_836.d	Acquisition Date	04/22/2022 13:55:33
Method	836.m COMPOUND 17	Instrument	amaZon ETD

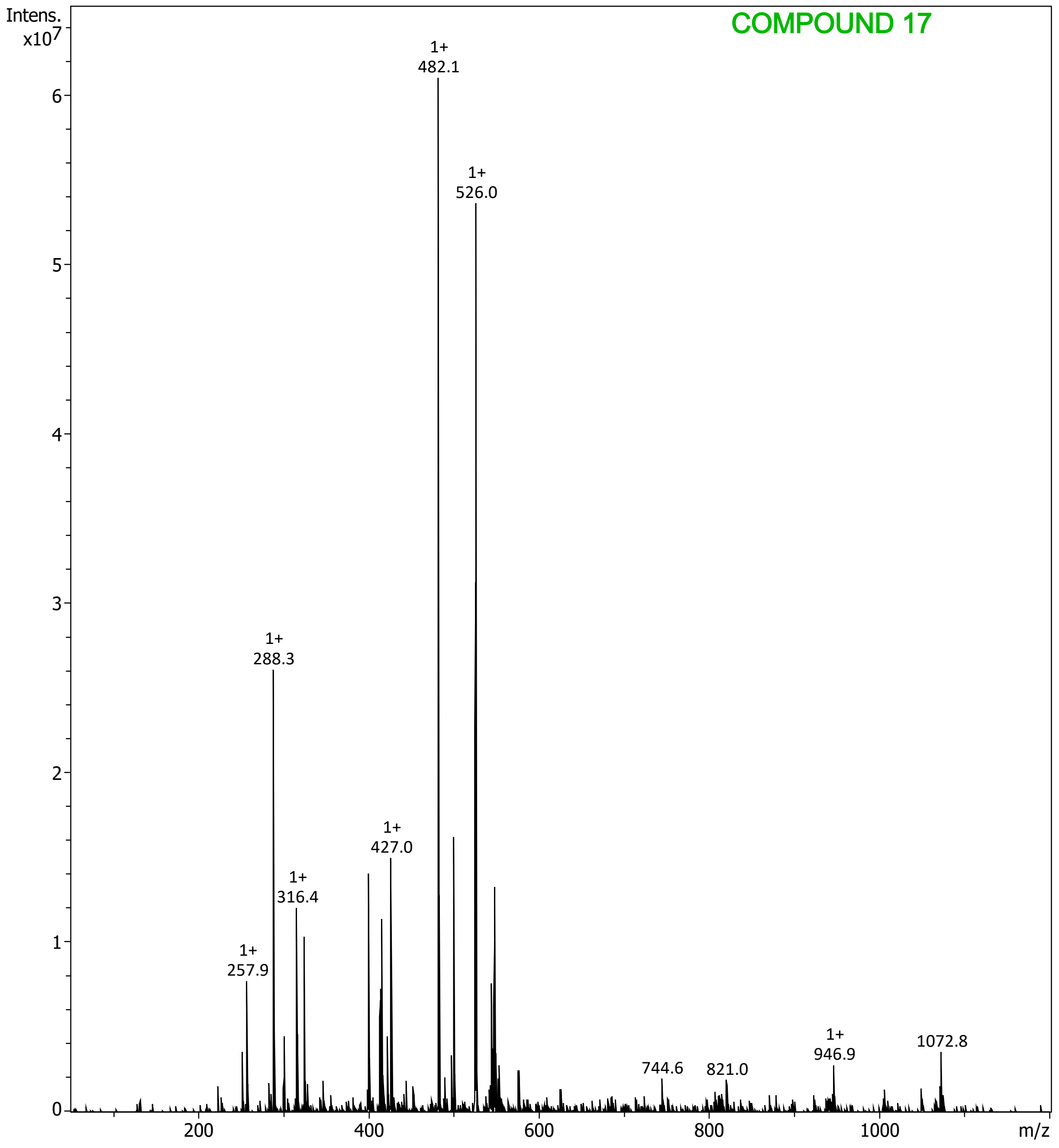

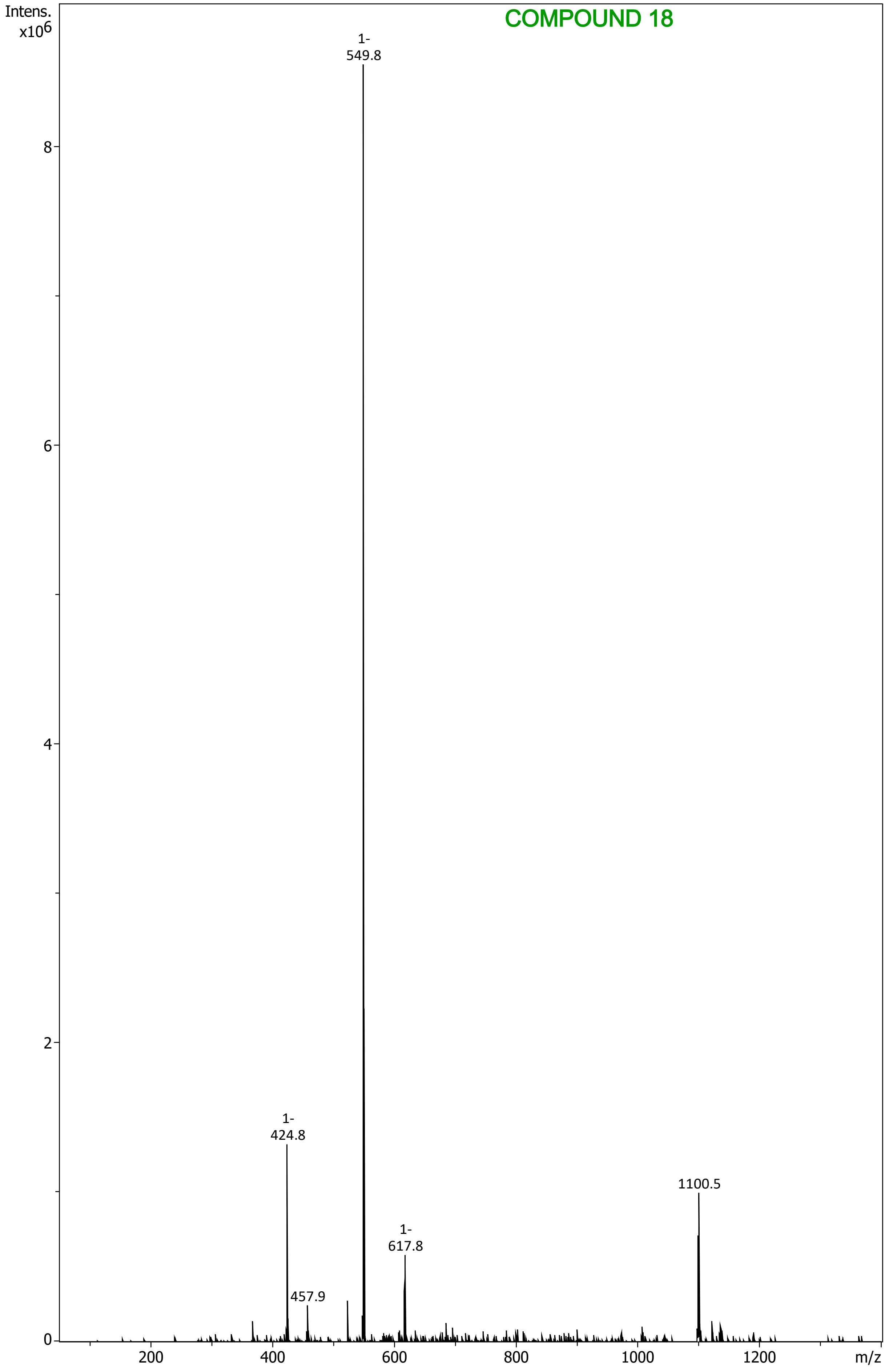
COMPOUND 18
Intens.
x10^6
1-
549.8
1-
424.8
457.9
1-
617.8
1100.5
0
2
4
6
8
200
400
600
800
1000
1200
m/z

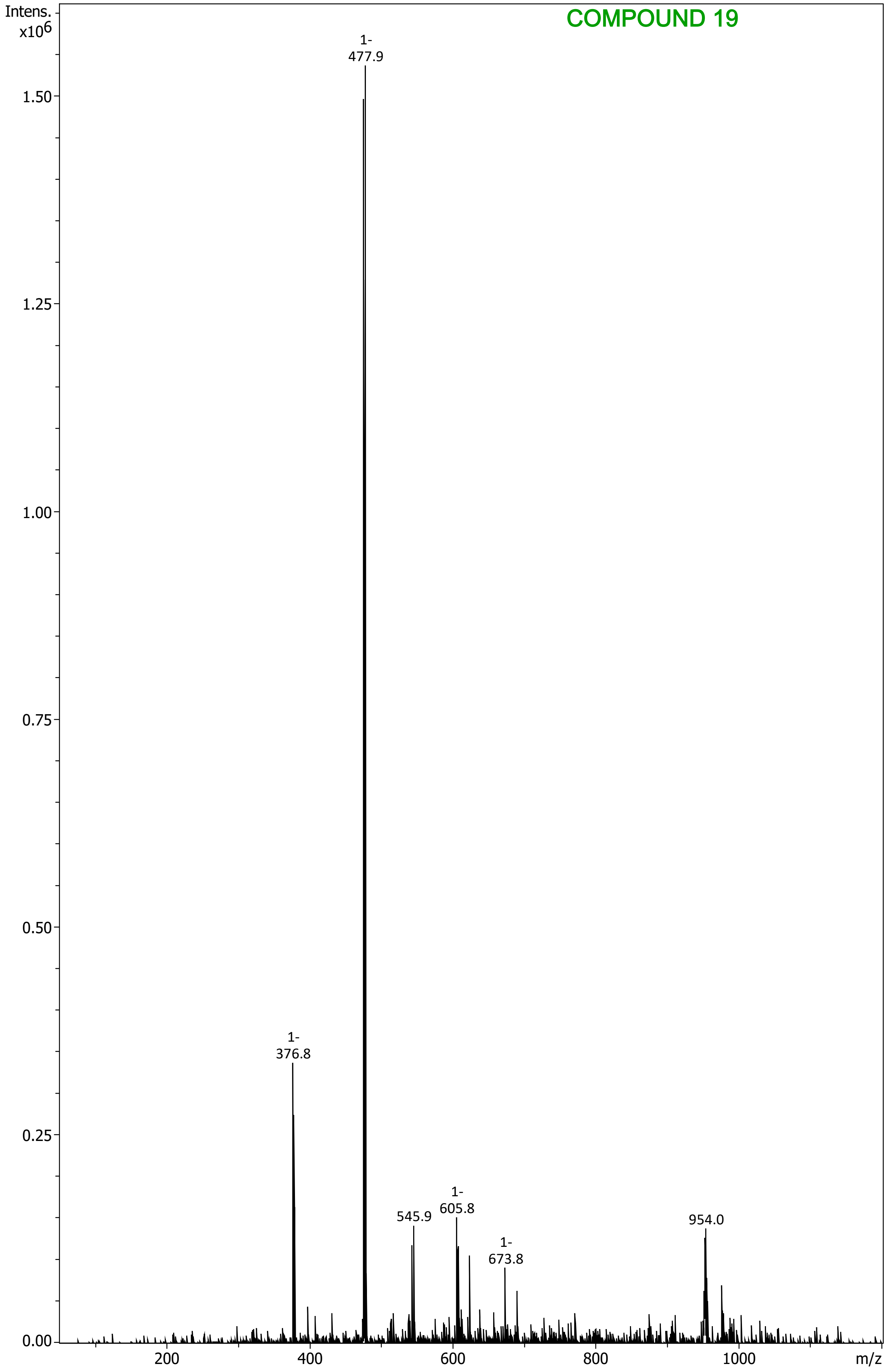
Intens.
x10^6
COMPOUND 19
1-
477.9
1.50
1.25
1.00
0.75
0.50
0.25
0.00
1-
376.8
545.9
1-
605.8
1-
673.8
954.0
200
400
600
800
1000
m/z

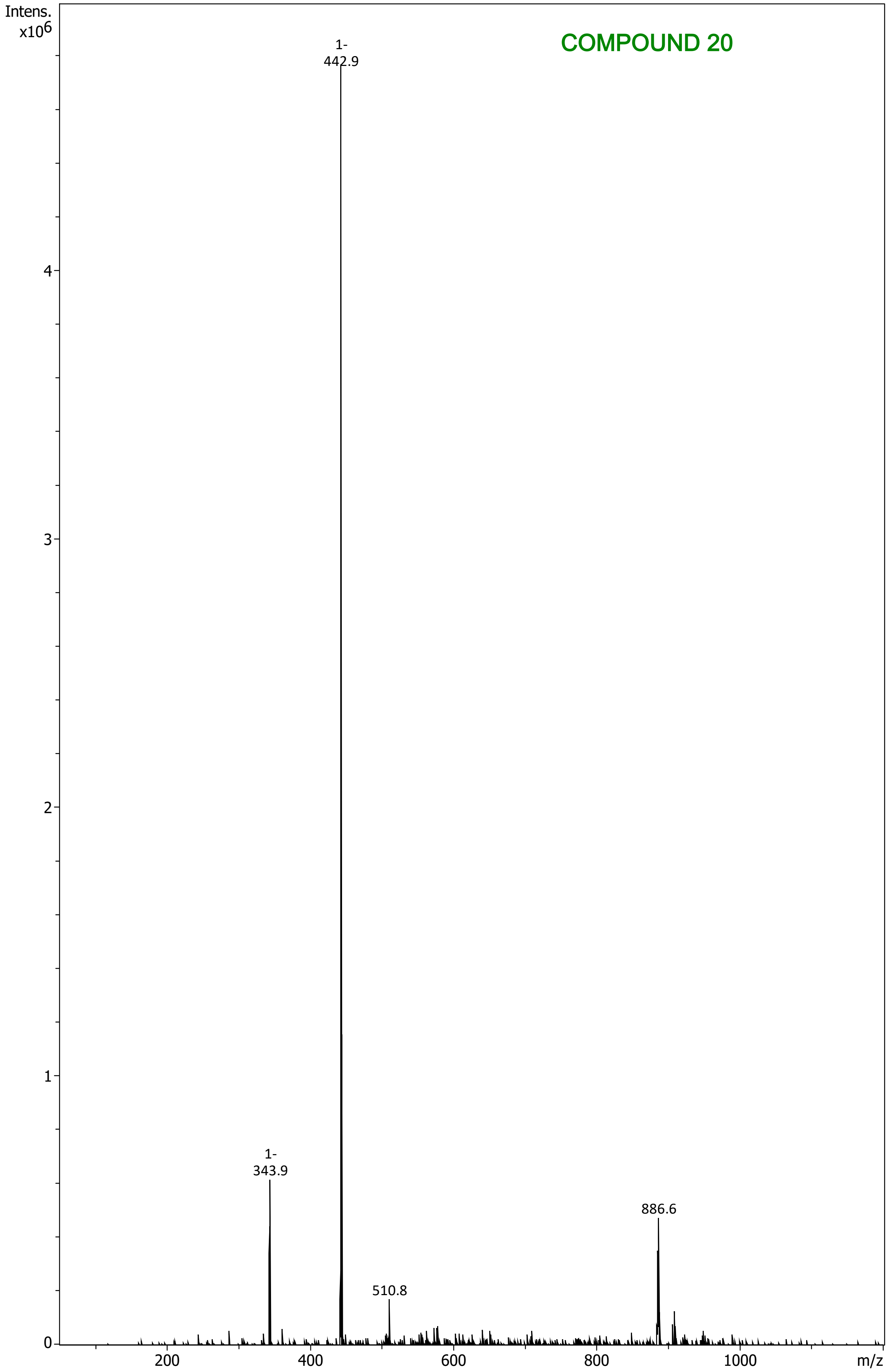
Intens.
x10^6
COMPOUND 20
1-
442.9
1-
343.9
886.6
510.8
4
3
2
1
0
200
400
600
800
1000
m/z

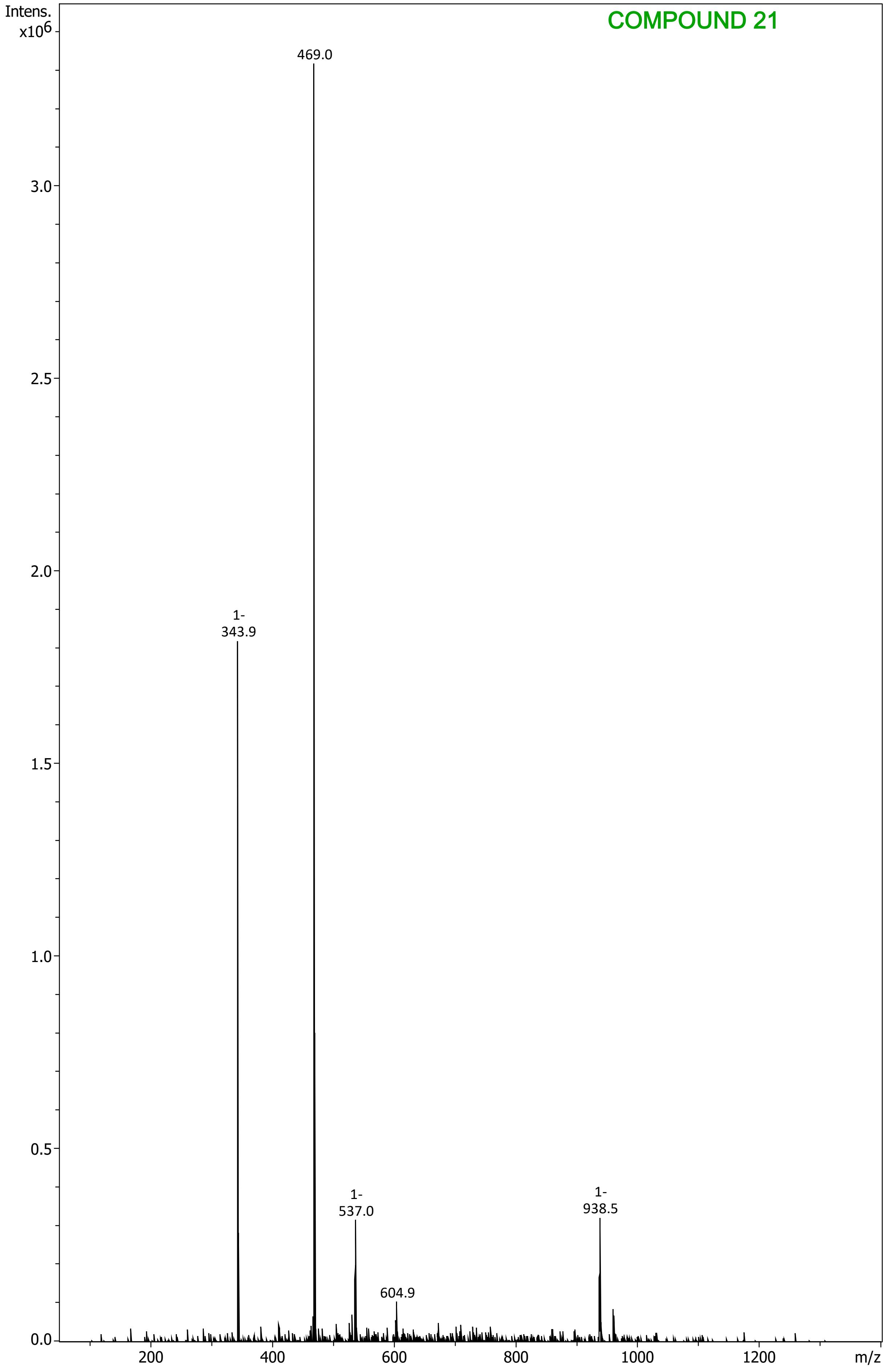

COMPOUND 21
Intens.
x10^6
469.0
1-
343.9
1-
537.0
604.9
1-
938.5
3.0
2.5
2.0
1.5
1.0
0.5
0.0
200
400
600
800
1000
1200
m/z

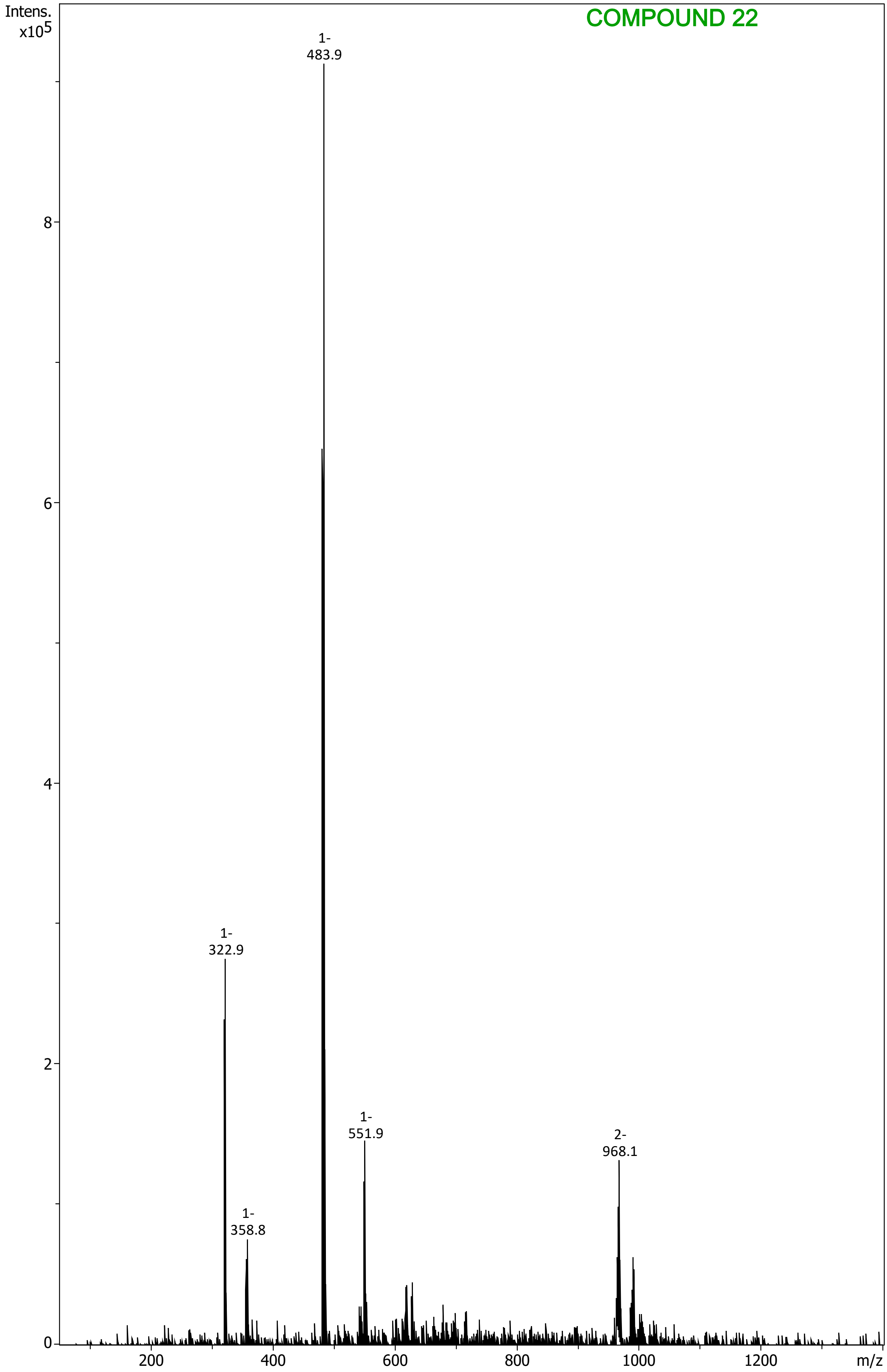

COMPOUND 22
Intens.
x10^5
1-
483.9
1-
322.9
1-
358.8
1-
551.9
2-
968.1
0
2
4
6
8
200
400
600
800
1000
1200
m/z

AREA DE INFRAESTRUCTURAS DE INVESTIGACION
UNIDADE DE ESPECTROMETRIA DE MASAS E PROTEÓMICA
Edificio CACTUS * Campus Vida
15782 Santiago de Compostela
Tel. 881816242. Ext. 16242
E-mail: sxemasas@usc.es
Web: https://www.usc.es/gl/investigacion/riaidt/espectrometria/

ESPECTRO ESI-FIA-Ion Trap

Analysis Info

Sample Name	17 ESI MF3-CM-179 317_59_01_1156.d	Acquisition Date	12/1/2022 4:00:32 PM
Method	1156.m	Instrument	amaZon ETD

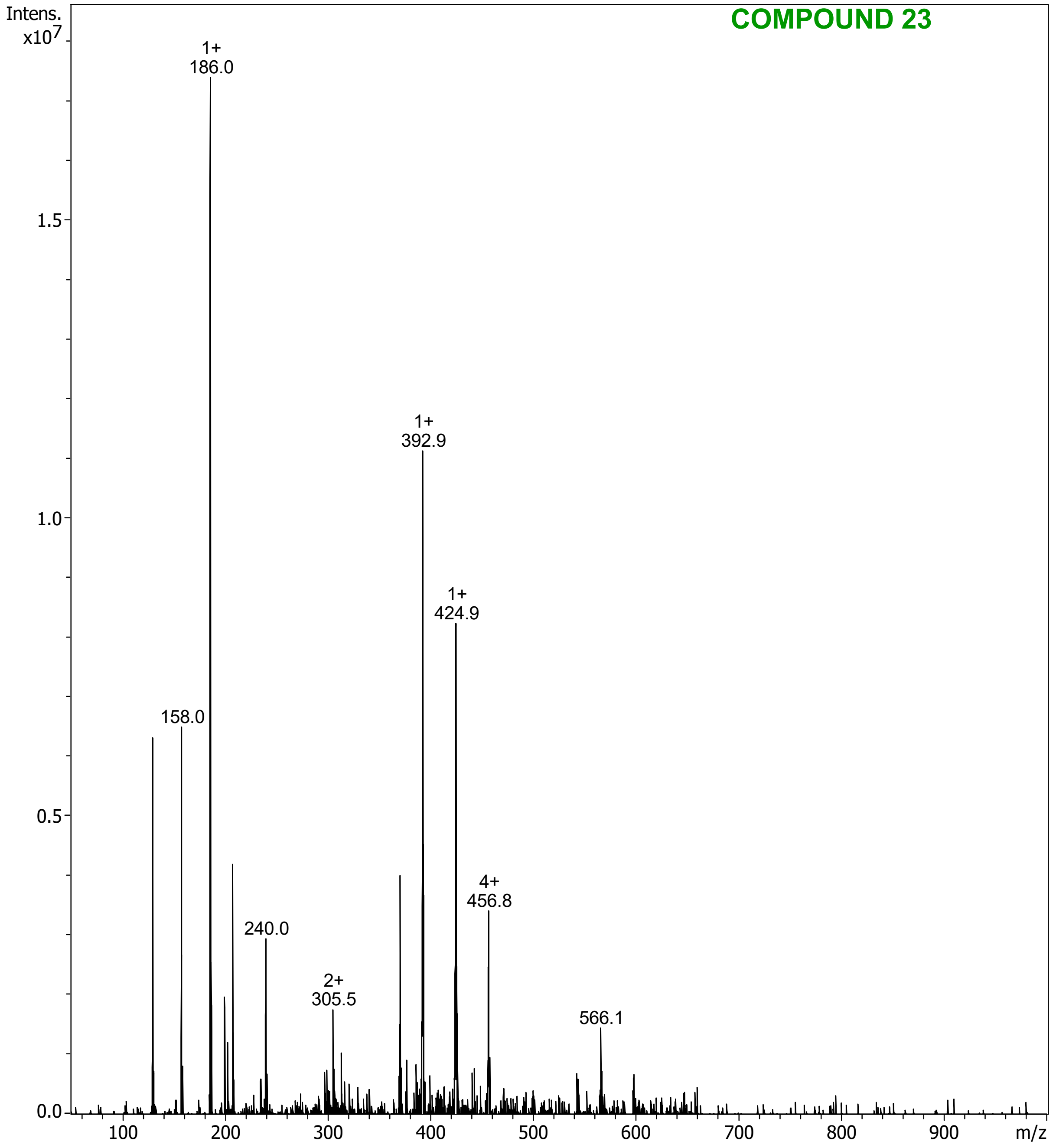

AREA DE INFRAESTRUCTURAS DE INVESTIGACION
UNIDADE DE ESPECTROMETRIA DE MASAS E PROTEÓMICA
Edificio CACTUS * Campus Vida
15782 Santiago de Compostela
Tel. 881816242. Ext. 16242
E-mail: sxemasas@usc.es
Web: https://www.usc.es/gl/investigacion/riaidt/espectrometria/

ESPECTRO ESI-FIA-Ion Trap

Analysis Info

Sample Name	9 ESI MF3-CM-181 310_52_01_1148.d	Acquisition Date	12/1/2022 2:31:54 PM
Method	1148.m	Instrument	amaZon ETD

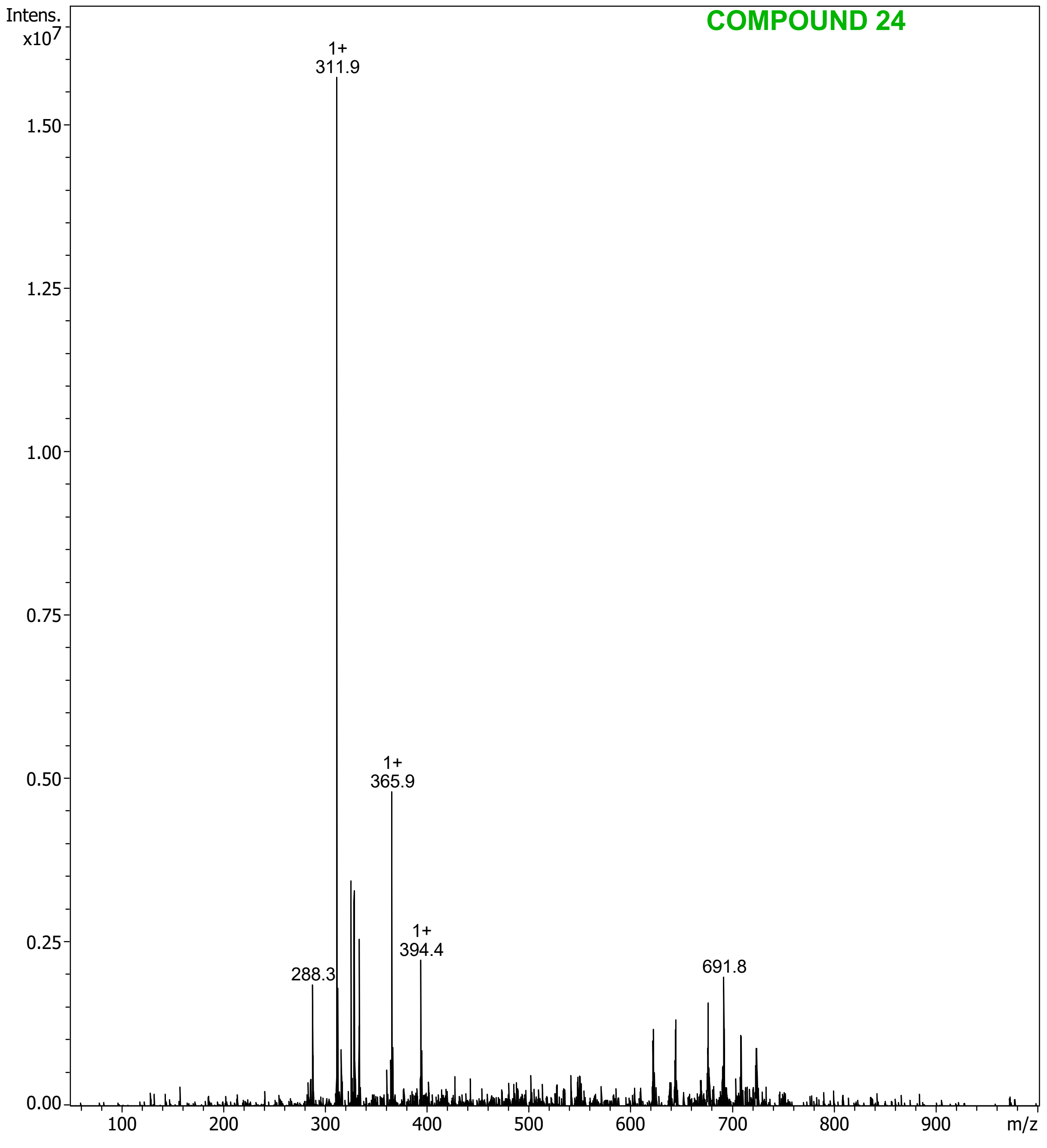

AREA DE INFRAESTRUCTURAS DE INVESTIGACION
UNIDADE DE ESPECTROMETRIA DE MASAS E PROTEÓMICA
Edificio CACTUS * Campus Vida
15782 Santiago de Compostela
Tel. 881816242. Ext. 16242
E-mail: sxemasas@usc.es
Web: https://www.usc.es/gl/investigacion/riaidt/espectrometria/

ESPECTRO ESI-FIA-Ion Trap

Analysis Info

Sample Name	15 ESI MF3-CM-193 318_57_01_1154.d	Acquisition Date	12/1/2022 3:38:22 PM
Method	1154.m	Instrument	amaZon ETD

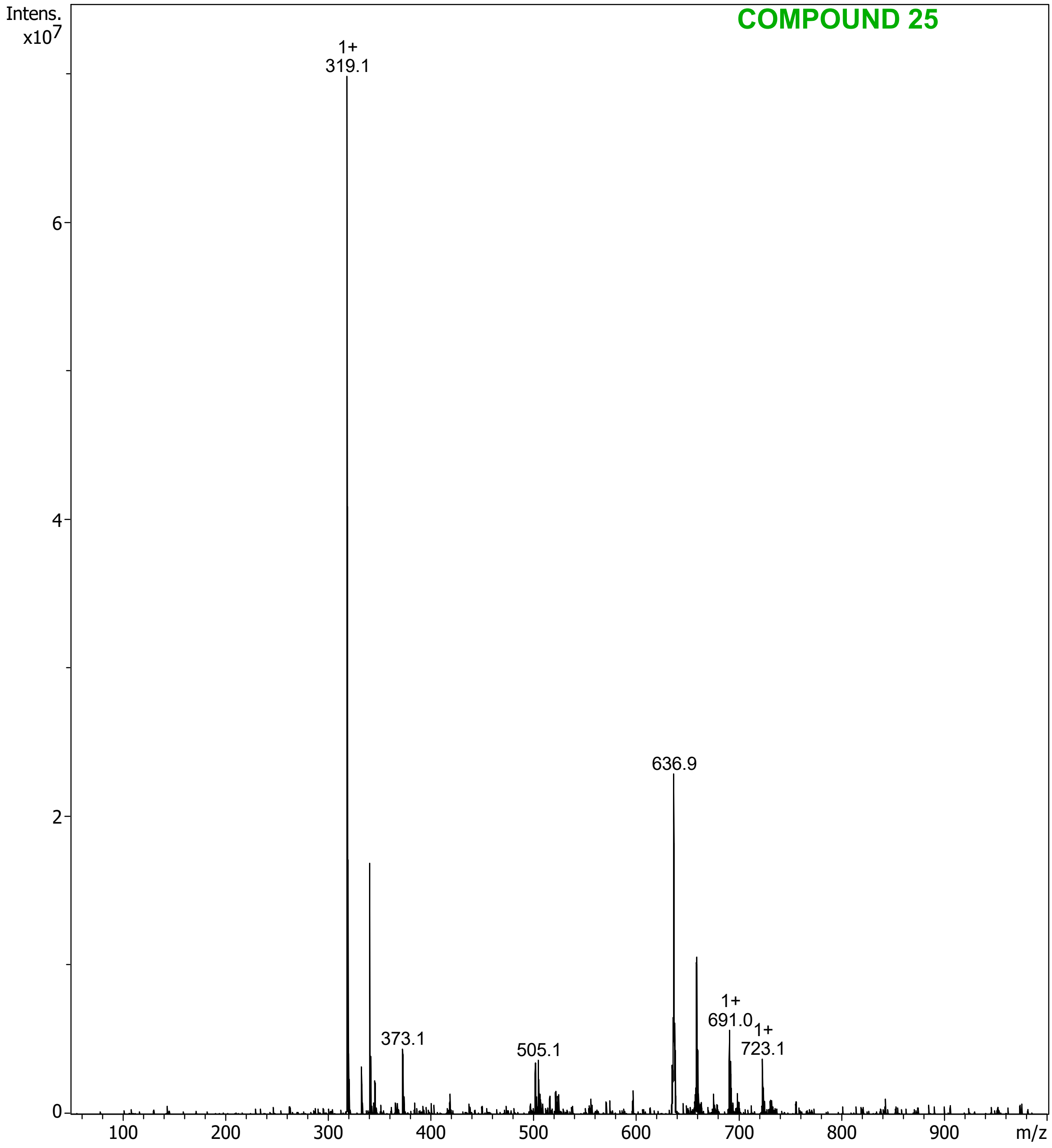

AREA DE INFRAESTRUCTURAS DE INVESTIGACION
UNIDADE DE ESPECTROMETRIA DE MASAS E PROTEÓMICA
Edificio CACTUS * Campus Vida
15782 Santiago de Compostela
Tel. 881816242. Ext. 16242
E-mail: sxemasas@usc.es
Web: https://www.usc.es/gl/investigacion/riaidt/espectrometria/

ESPECTRO ESI-FIA-Ion Trap

Analysis Info

Sample Name	10 ESI MF3-CM-182B 444_53_01_1149.d	Acquisition Date	12/1/2022 2:42:58 PM
Method	1149.m	Instrument	amaZon ETD

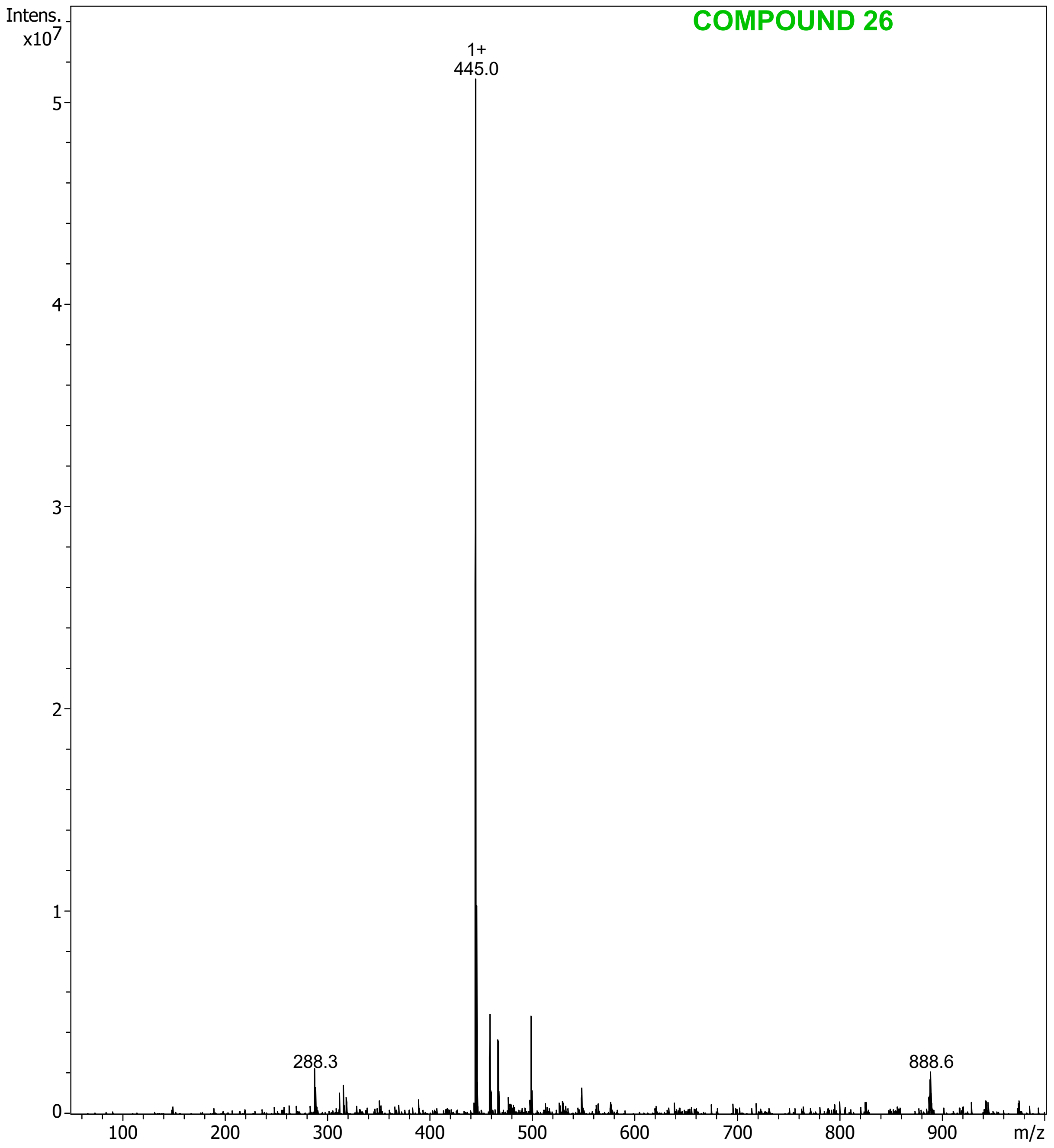

AREA DE INFRAESTRUCTURAS DE INVESTIGACION
UNIDADE DE ESPECTROMETRIA DE MASAS E PROTEÓMICA
Edificio CACTUS * Campus Vida
15782 Santiago de Compostela
Tel. 881816242. Ext. 16242
E-mail: sxemasas@usc.es
Web: https://www.usc.es/gl/investigacion/riaidt/espectrometria/

ESPECTRO ESI-FIA-Ion Trap

Analysis Info

Sample Name	11 ESI MF3-CM-190 439_54_01_1150.d	Acquisition Date	12/1/2022 2:54:01 PM
Method	1150.m	Instrument	amaZon ETD

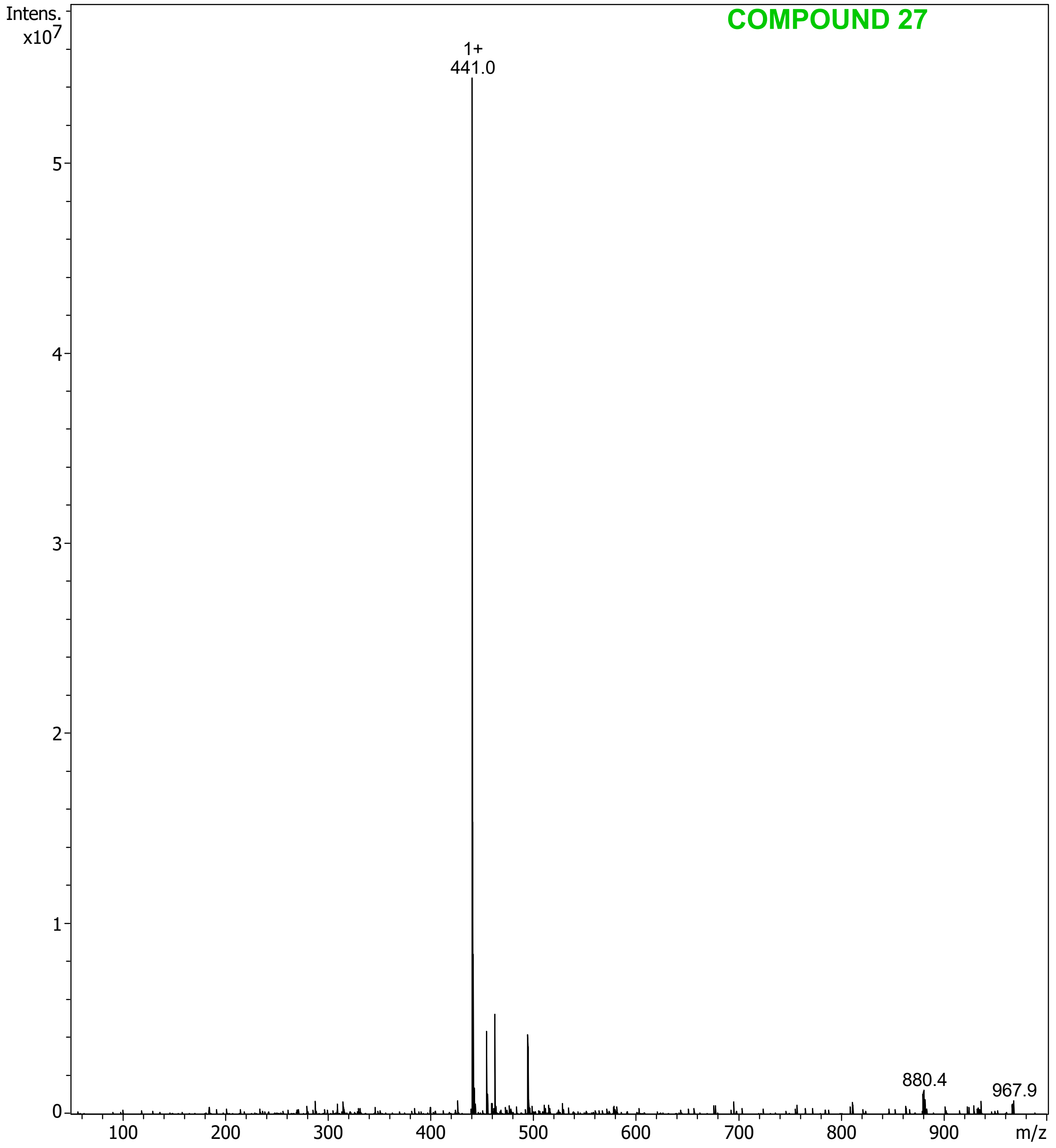

AREA DE INFRAESTRUCTURAS DE INVESTIGACION
UNIDADE DE ESPECTROMETRIA DE MASAS E PROTEÓMICA
Edificio CACTUS * Campus Vida
15782 Santiago de Compostela
Tel. 881816242. Ext. 16242
E-mail: sxemasas@usc.es
Web: https://www.usc.es/gl/investigacion/riaidt/espectrometria/

ESPECTRO ESI-FIA-Ion Trap

Analysis Info

Sample Name	12 ESI MF3-CM-186 488_55_01_1151.d	Acquisition Date	12/1/2022 3:05:06 PM
Method	1151.m	Instrument	amaZon ETD

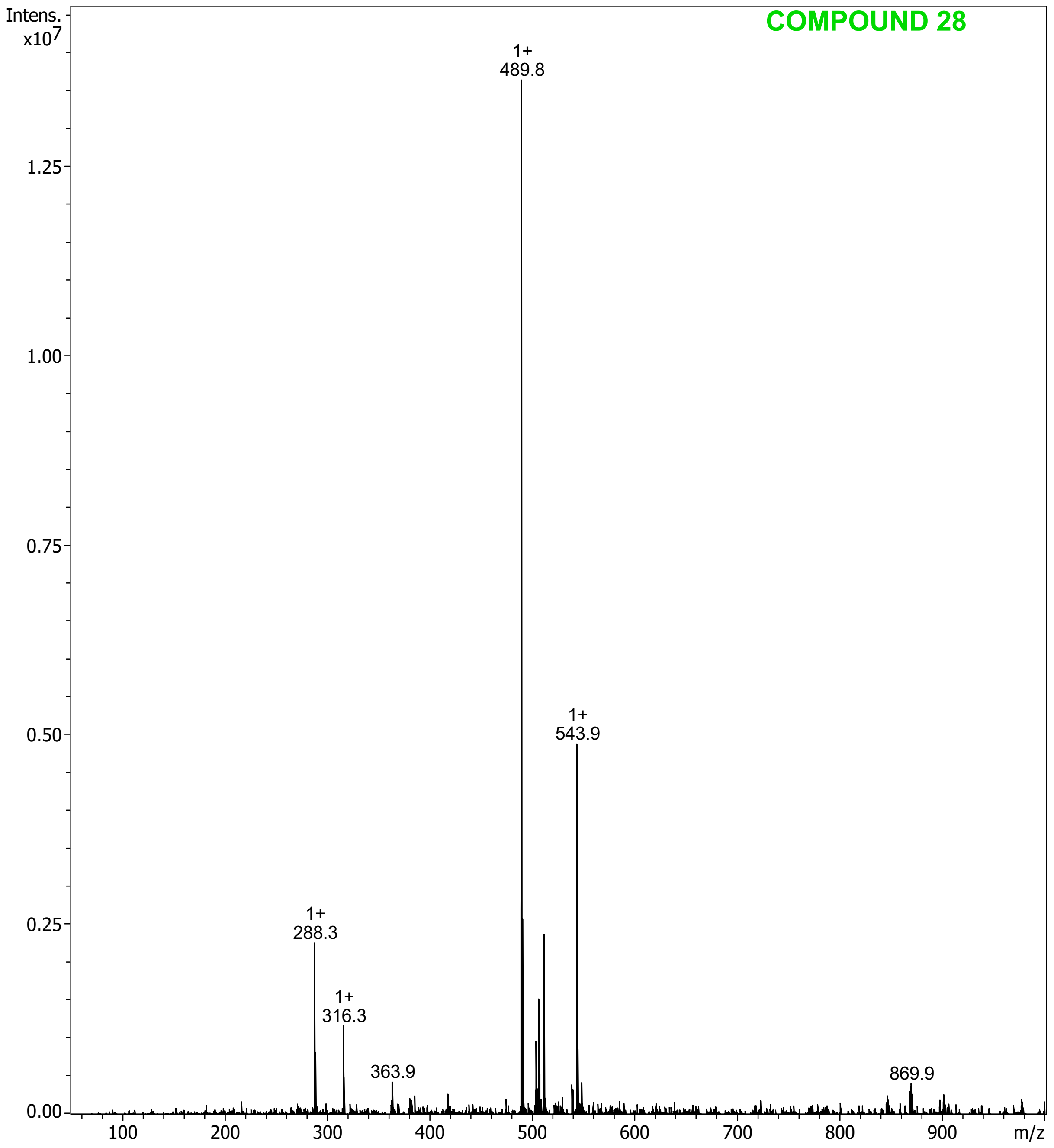

AREA DE INFRAESTRUCTURAS DE INVESTIGACION
UNIDADE DE ESPECTROMETRIA DE MASAS E PROTEÓMICA
Edificio CACTUS * Campus Vida
15782 Santiago de Compostela
Tel. 881816242. Ext. 16242
E-mail: sxemasas@usc.es
Web: https://www.usc.es/gl/investigacion/riaidt/espectrometria/

ESPECTRO ESI-FIA-Ion Trap

Analysis Info

Sample Name	4 ESI MF3-CM-105 362_42_01_833.d	Acquisition Date	04/22/2022 13:22:14
Method	833.m	Instrument	amaZon ETD

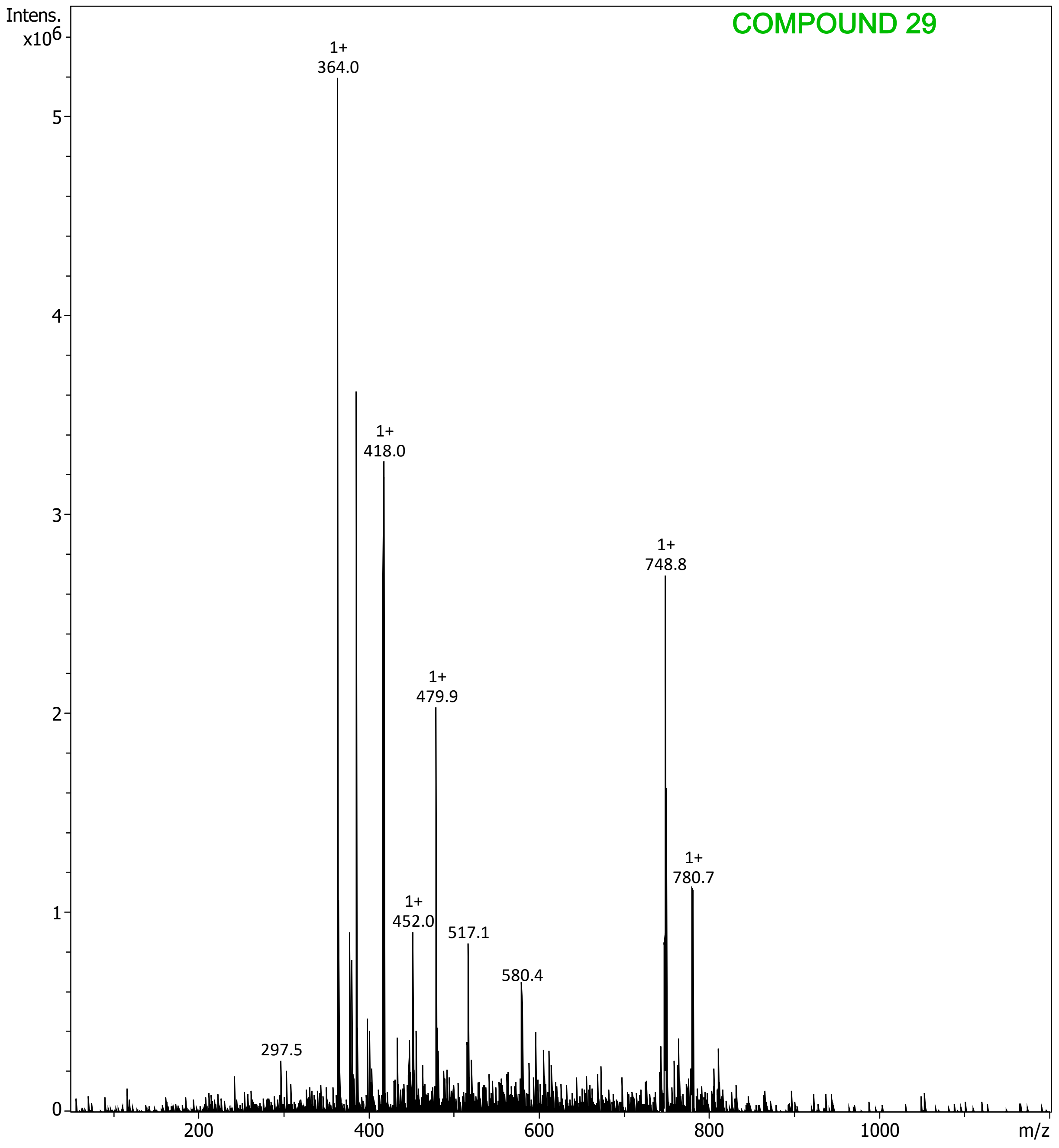

AREA DE INFRAESTRUCTURAS DE INVESTIGACION
UNIDADE DE ESPECTROMETRIA DE MASAS E PROTEÓMICA
Edificio CACTUS * Campus Vida
15782 Santiago de Compostela
Tel. 881816242. Ext. 16242
E-mail: sxemasas@usc.es
Web: https://www.usc.es/gl/investigacion/riaidt/espectrometria/

ESPECTRO ESI-FIA-Ion Trap

Analysis Info

Sample Name	3 ESI MF3-CM-171 317_43_01_1187.d	Acquisition Date	12/21/2022 13:30:09
Method	1187.m	Instrument	amaZon ETD

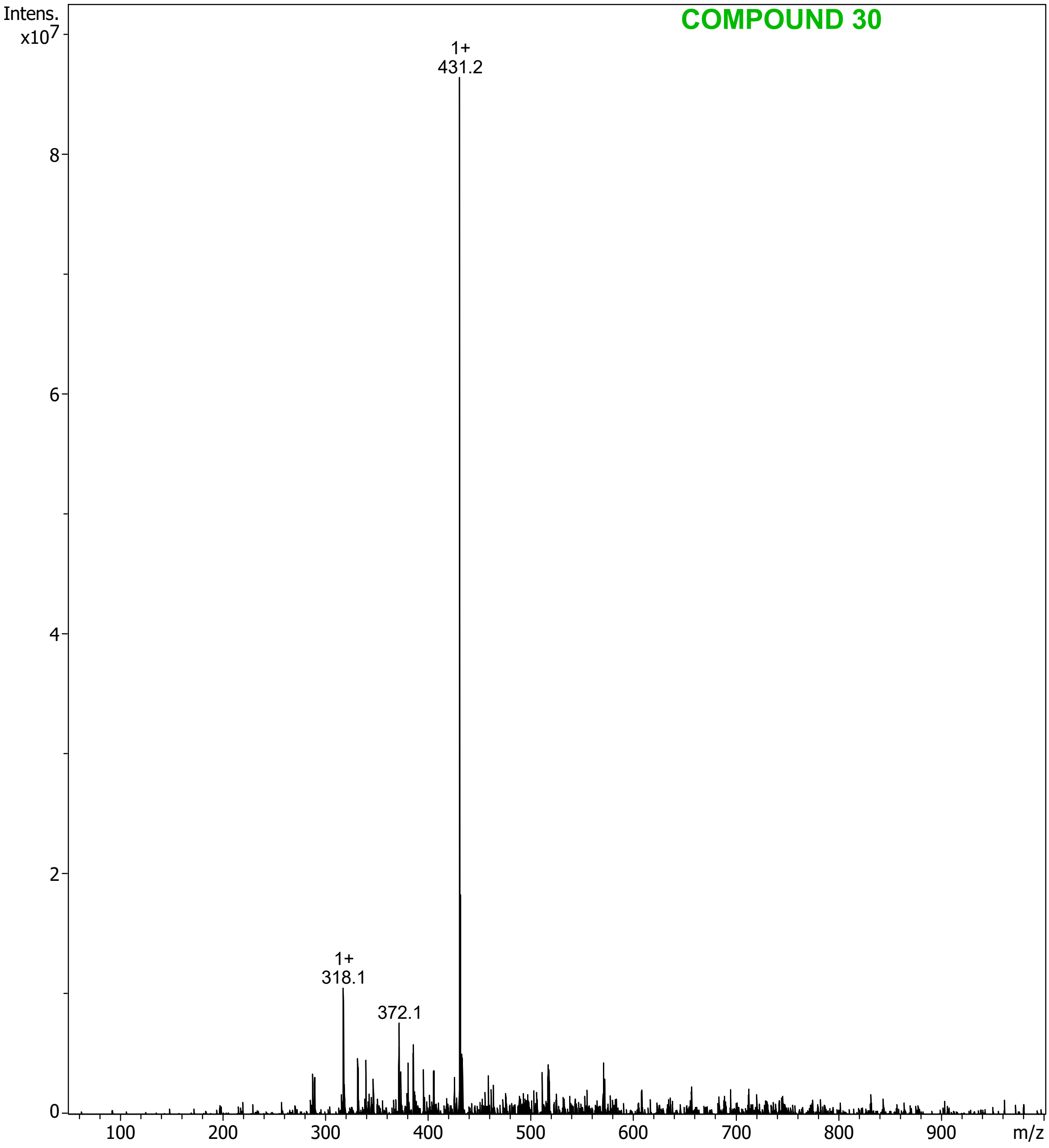

AREA DE INFRAESTRUCTURAS DE INVESTIGACION
UNIDADE DE ESPECTROMETRIA DE MASAS E PROTEÓMICA

Edificio CACTUS * Campus Vida
15782 Santiago de Compostela
Tel. 881816242. Ext. 16242
E-mail: sxemasas@usc.es
Web: https://www.usc.es/gl/investigacion/riaidt/espectrometria/

ESPECTRO ESI-FIA-TOF

Analysis Info

Sample Name	16 ESI MF3-CM-164 321_362_1_3271.d	Acquisition Date	05/13/2022 13:08:38
Method	FIA ESI POSITIVO 50-1500.m	Instrument	impact II

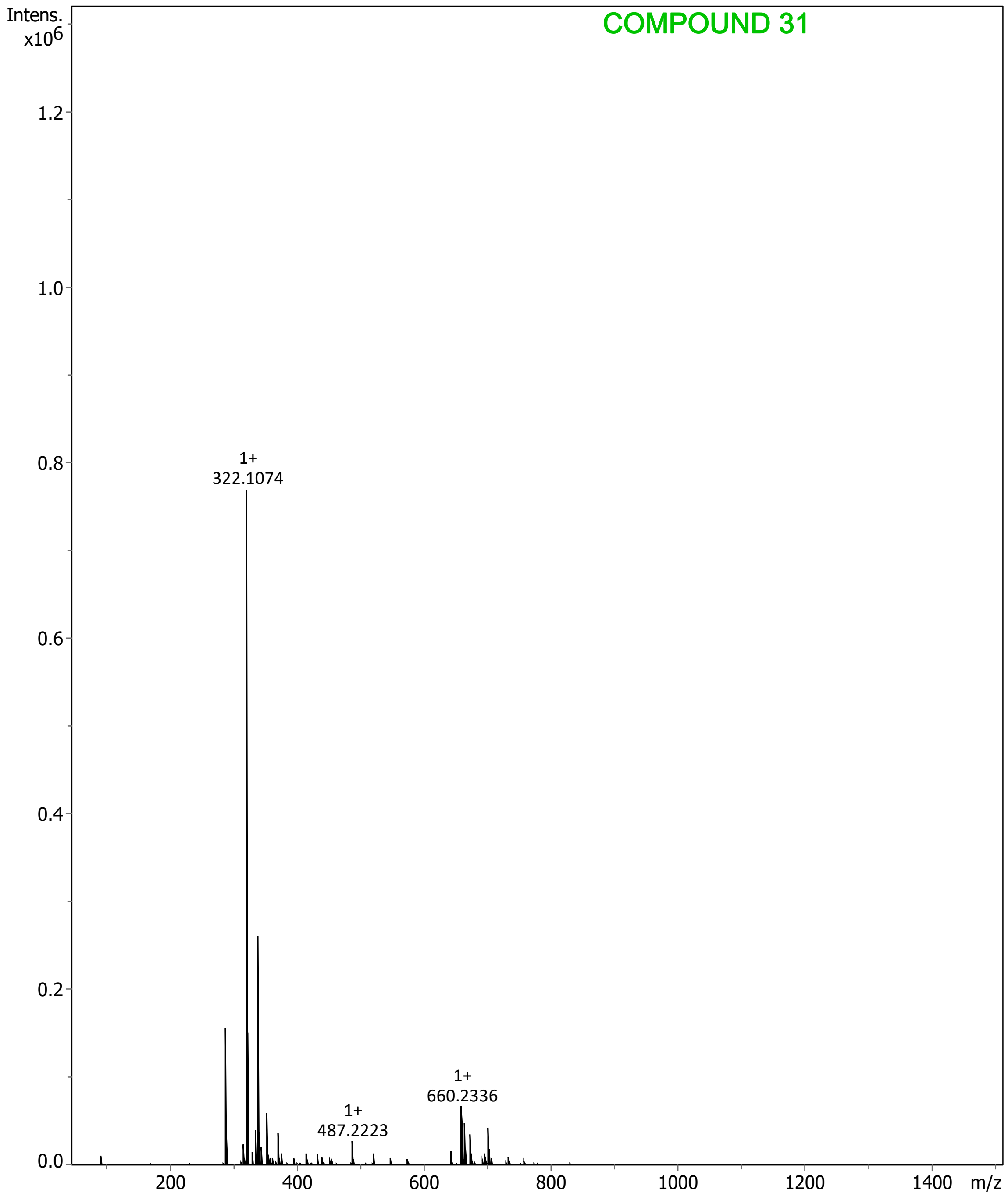

AREA DE INFRAESTRUCTURAS DE INVESTIGACION
UNIDADE DE ESPECTROMETRIA DE MASAS E PROTEÓMICA

Edificio CACTUS * Campus Vida
15782 Santiago de Compostela
Tel. 881816242. Ext. 16242
E-mail: sxemasas@usc.es
Web: https://www.usc.es/gl/investigacion/riaidt/espectrometria/

ESPECTRO ESI-FIA-Ion Trap

Analysis Info

Sample Name	14 ESI MF3-CM-188A 447_56_01_1153.d	Acquisition Date	12/1/2022 3:27:17 PM
Method	1153.m	Instrument	amaZon ETD

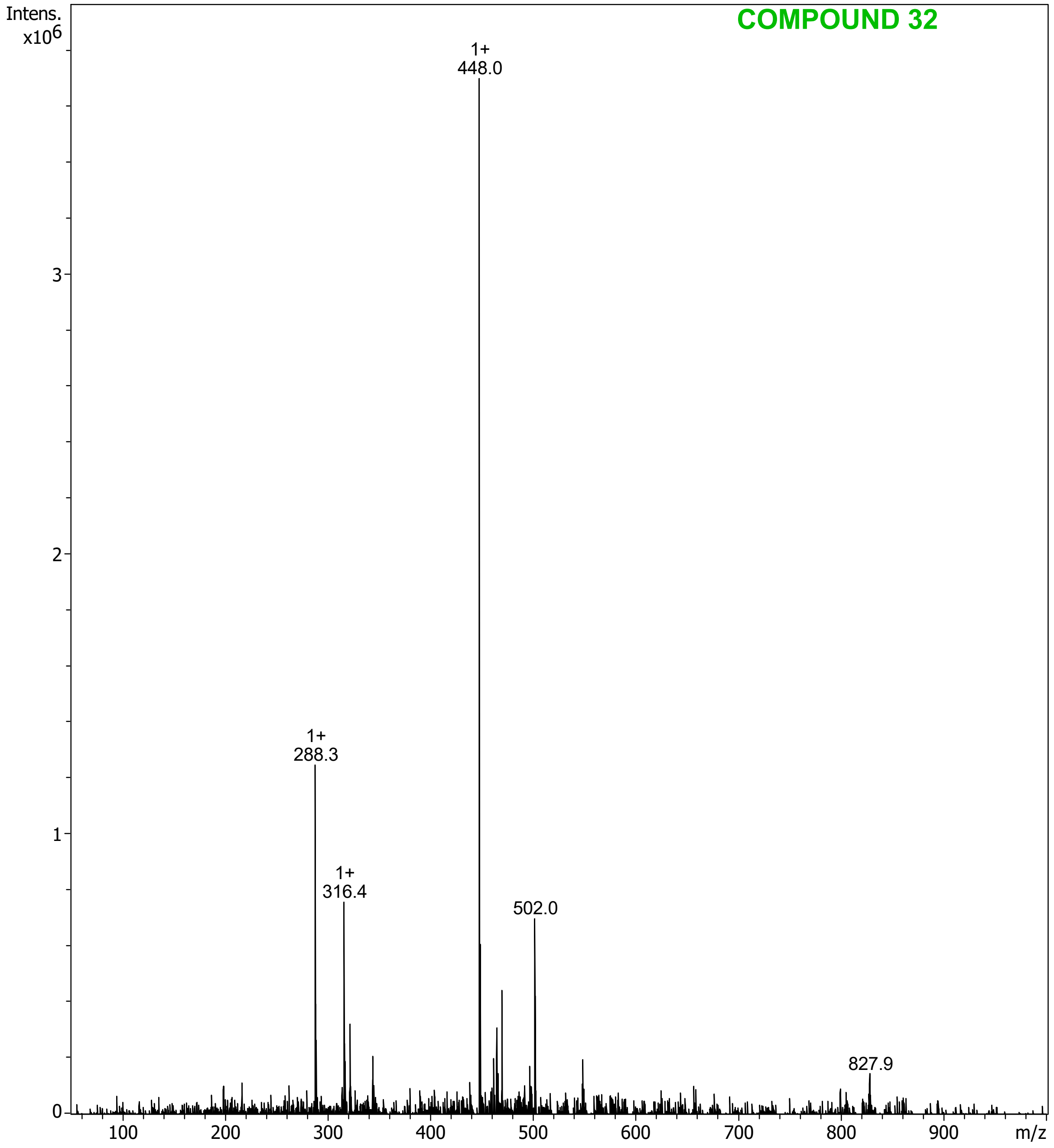

AREA DE INFRAESTRUCTURAS DE INVESTIGACION
UNIDADE DE ESPECTROMETRIA DE MASAS E PROTEÓMICA
Edificio CACTUS * Campus Vida
15782 Santiago de Compostela
Tel. 881816242. Ext. 16242
E-mail: sxemasas@usc.es
Web: https://www.usc.es/gl/investigacion/riaidt/espectrometria/

ESPECTRO ESI-FIA-Ion Trap

Analysis Info

Sample Name	16 ESI MF3-CM-188b 446_58_01_1155.d	Acquisition Date	12/1/2022 3:49:27 PM
Method	1155.m	Instrument	amaZon ETD

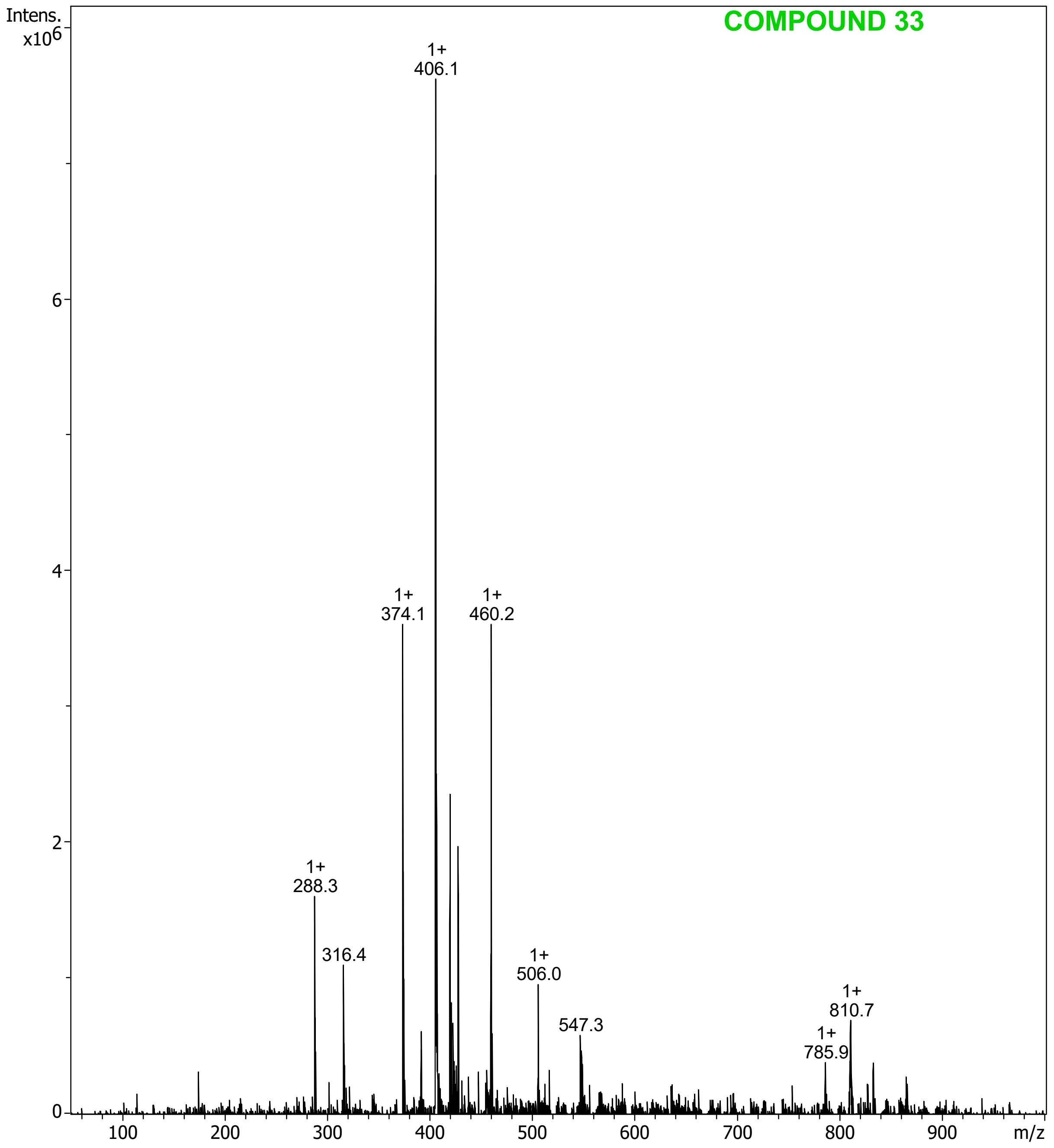

5. X-ray crystalographic data of compound 17

checkCIF/PLATON report

Structure factors have been supplied for datablock(s) 22FMB001

THIS REPORT IS FOR GUIDANCE ONLY. IF USED AS PART OF A REVIEW PROCEDURE FOR PUBLICATION, IT SHOULD NOT REPLACE THE EXPERTISE OF AN EXPERIENCED CRYSTALLOGRAPHIC REFEREE.

No syntax errors found. CIF dictionary Interpreting this report

Datablock: 22FMB001

```
Bond precision:   C-C = 0.0012 A           Wavelength=0.71073

Cell:           a=6.6703(4)        b=10.0801(6)      c=16.8273(10)
                alpha=83.649(2)    beta=86.849(2)    gamma=74.054(2)
Temperature:    100 K
```

	Calculated	Reported
Volume	1080.85(11)	1080.85(11)
Space group	P -1	P -1
Hall group	-P 1	-P 1
Moiety formula	C19 H23 F3 I N3 O3	C19 H23 F3 I N3 O3
Sum formula	C19 H23 F3 I N3 O3	C19 H23 F3 I N3 O3
Mr	525.30	525.30
Dx,g cm-3	1.614	1.614
Z	2	2
Mu (mm-1)	1.531	1.531
F000	524.0	524.0
F000'	523.32	
h,k,lmax	11,17,30	11,17,30
Nref	12881	12861
Tmin,Tmax	0.745,0.908	0.850,0.910
Tmin'	0.716	

```
Correction method= # Reported T Limits: Tmin=0.850 Tmax=0.910
AbsCorr = MULTI-SCAN

Data completeness= 0.998            Theta(max)= 39.390

R(reflections)= 0.0200( 11865)             wR2(reflections)= 0.0476( 12861)

S = 1.053                   Npar= 331
```

The following ALERTS were generated. Each ALERT has the format
test-name_ALERT_alert-type_alert-level.
Click on the hyperlinks for more details of the test.

Alert level C

```
PLAT220_ALERT_2_C NonSolvent   Resd 1  C   Ueq(max)/Ueq(min) Range        3.1 Ratio
```

Alert level G

```
PLAT154_ALERT_1_G The s.u.'s on the Cell Angles are Equal ..(Note)      0.002 Degree
PLAT164_ALERT_4_G Nr. of Refined C-H H-Atoms in Heavy-Atom Struct.         21 Note
PLAT793_ALERT_4_G Model has Chirality at C8          (Centro SPGR)          R Verify
PLAT910_ALERT_3_G Missing # of FCF Reflection(s) Below Theta(Min).          2 Note
PLAT912_ALERT_4_G Missing # of FCF Reflections Above STh/L=  0.600         17 Note
PLAT978_ALERT_2_G Number C-C Bonds with Positive Residual Density.         11 Info
```

0 **ALERT level A** = Most likely a serious problem - resolve or explain
0 **ALERT level B** = A potentially serious problem, consider carefully
1 **ALERT level C** = Check. Ensure it is not caused by an omission or oversight
6 **ALERT level G** = General information/check it is not something unexpected

1 ALERT type 1 CIF construction/syntax error, inconsistent or missing data
2 ALERT type 2 Indicator that the structure model may be wrong or deficient
1 ALERT type 3 Indicator that the structure quality may be low
3 ALERT type 4 Improvement, methodology, query or suggestion
0 ALERT type 5 Informative message, check

Validation response form

Please find below a validation response form (VRF) that can be filled in and pasted into your CIF.

```
# start Validation Reply Form
_vrf_PLAT220_22FMB001
;
PROBLEM: NonSolvent   Resd 1  C   Ueq(max)/Ueq(min) Range        3.1 Ratio
RESPONSE: ...
;
# end Validation Reply Form
```

It is advisable to attempt to resolve as many as possible of the alerts in all categories. Often the minor alerts point to easily fixed oversights, errors and omissions in your CIF or refinement strategy, so attention to these fine details can be worthwhile. In order to resolve some of the more serious problems it may be necessary to carry out additional measurements or structure refinements. However, the purpose of your study may justify the reported deviations and the more serious of these should normally be commented upon in the discussion or experimental section of a paper or in the "special_details" fields of the CIF. checkCIF was carefully designed to identify outliers and unusual parameters, but every test has its limitations and alerts that are not important in a particular case may appear. Conversely, the absence of alerts does not guarantee there are no aspects of the results needing attention. It is up to the individual to critically assess their own results and, if necessary, seek expert advice.

Publication of your CIF in IUCr journals

A basic structural check has been run on your CIF. These basic checks will be run on all CIFs submitted for publication in IUCr journals (*Acta Crystallographica*, *Journal of Applied Crystallography*, *Journal of Synchrotron Radiation*); however, if you intend to submit to *Acta Crystallographica Section C* or *E* or *IUCrData*, you should make sure that full publication checks are run on the final version of your CIF prior to submission.

Publication of your CIF in other journals

Please refer to the *Notes for Authors* of the relevant journal for any special instructions relating to CIF submission.

PLATON version of 18/05/2022; check.def file version of 17/05/2022

Datablock 22FMB001 - ellipsoid plot

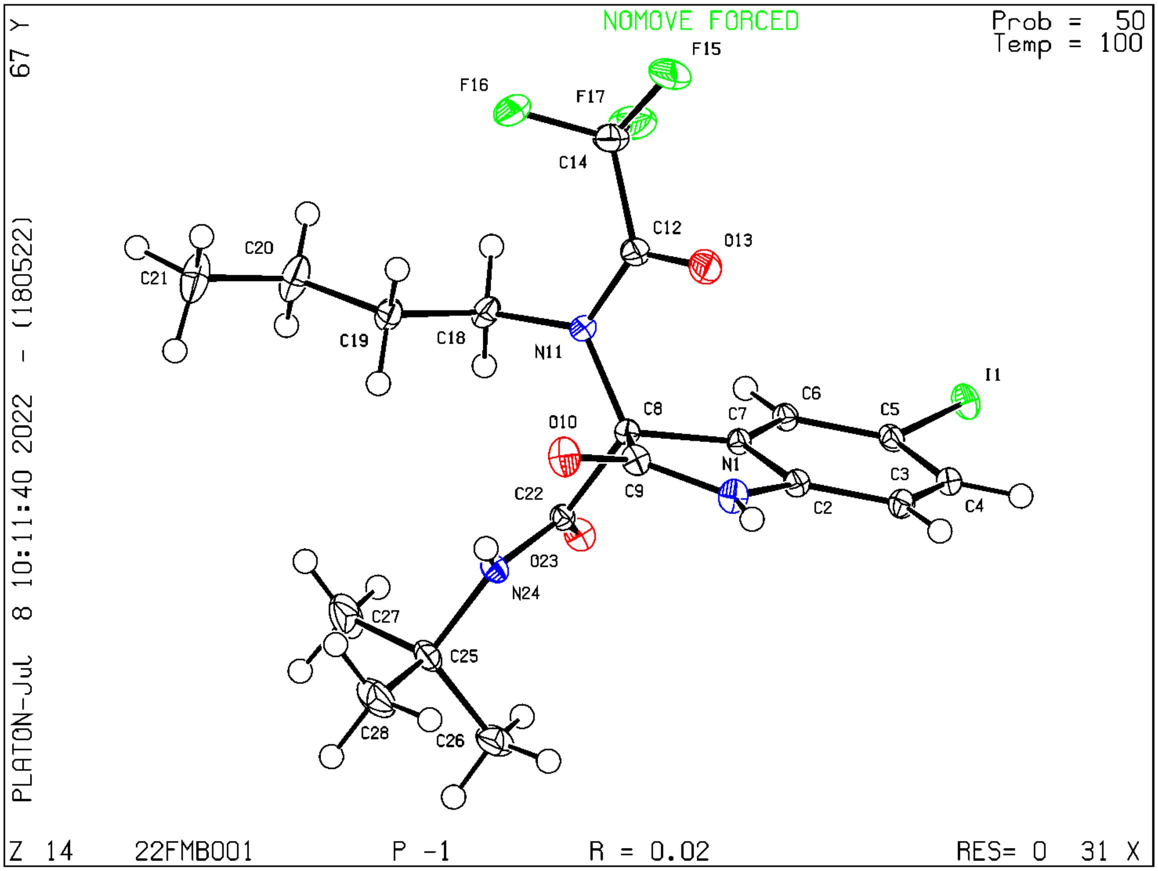

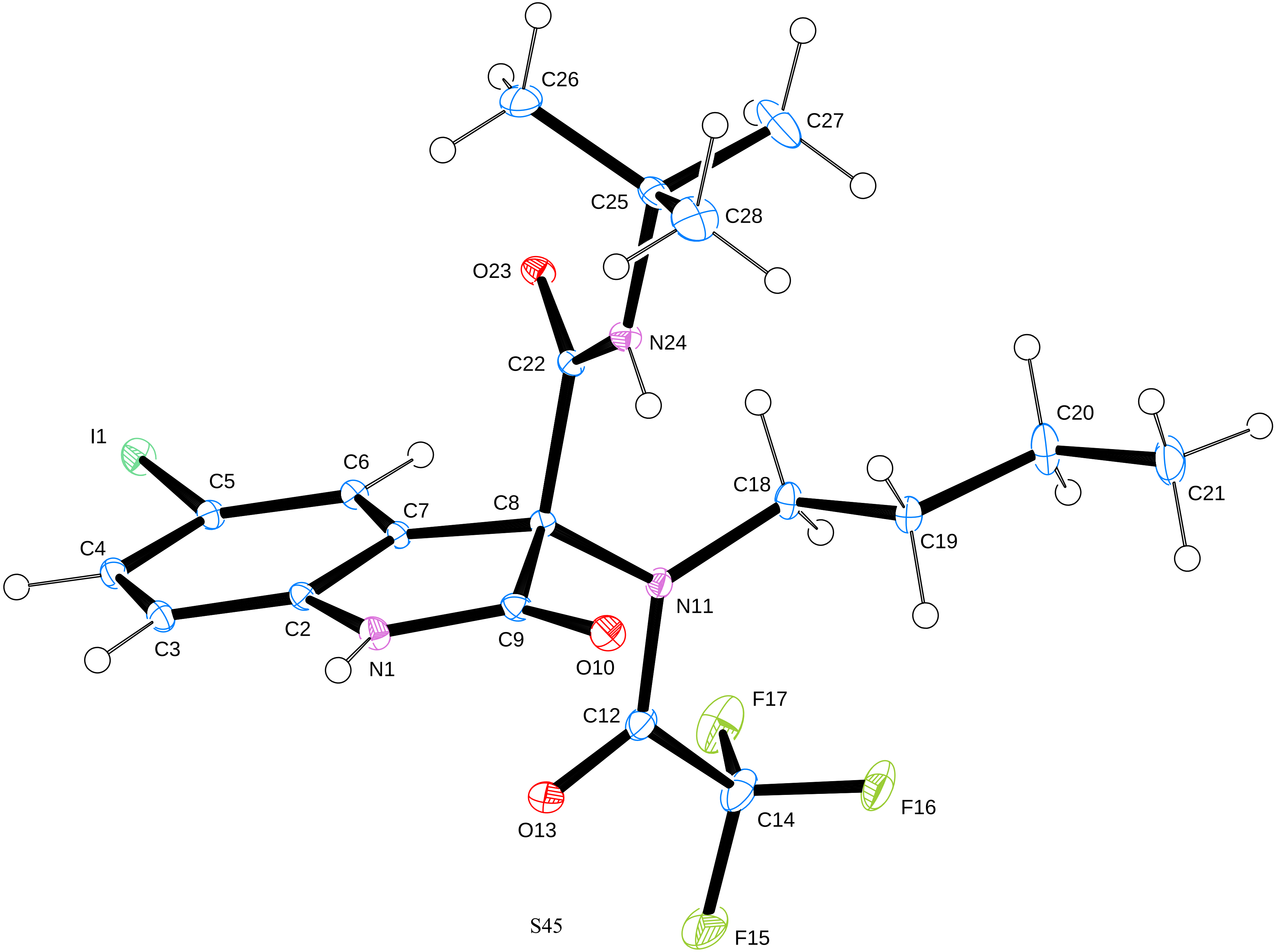
C26
C27
C25
C28
O23
N24
C22
C20
I1
C6
C5
C18
C21
C7
C8
C19
C4
N11
C2
C3
C9
N1
O10
F17
C12
F16
C14
O13
F15

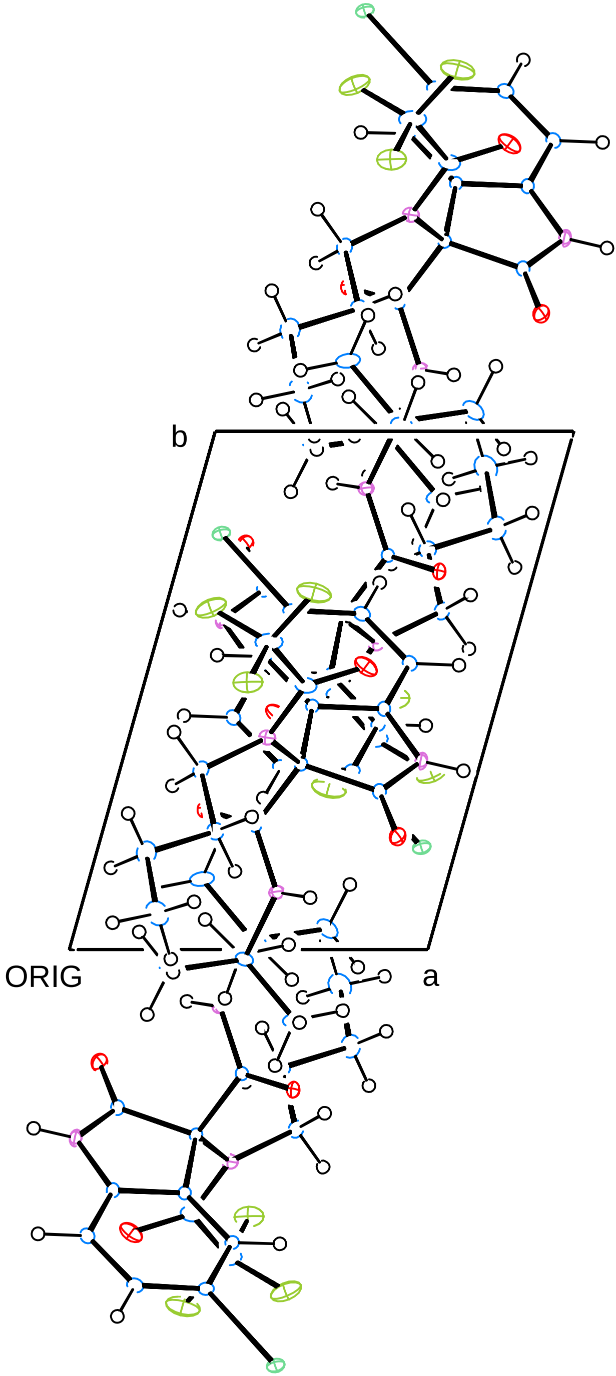

b
ORIG
a

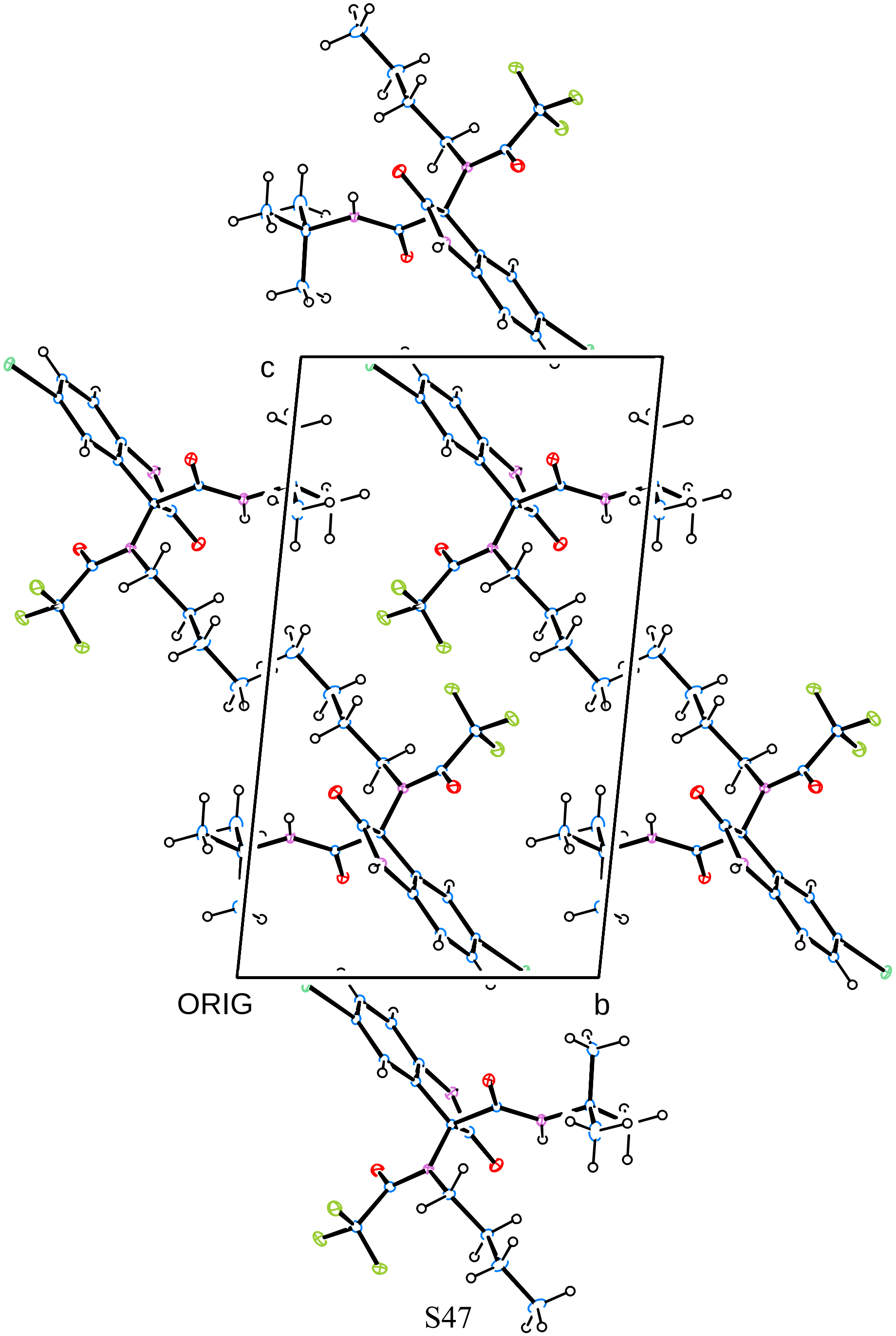
c
ORIG
b

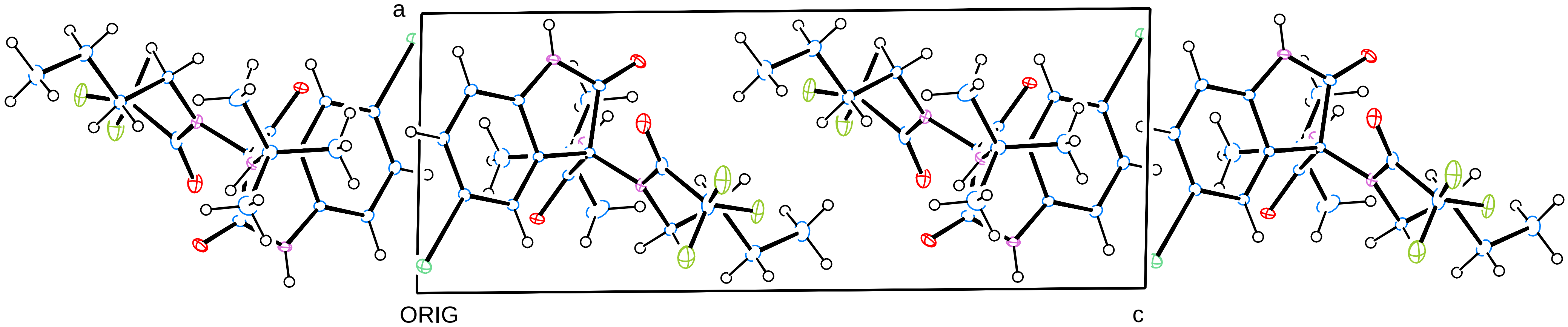
a
ORIG
c

Title

Antonio L. Llamas–Saiz

X-Ray Unit. Research Infrastructures Area, CACTUS Bldg., Campus VIDA, University of Santiago de Compostela, E–15782 Santiago de Compostela, Spain

Abstract

Related literature

Computing details

Data collection: Bruker *APEX4* software; cell refinement: *SAINT* V8.40B (Bruker AXS LLC, 2019); data reduction: *SAINT* V8.40B (Bruker AXS LLC, 2019); program(s) used to solve structure: *SHELXT* 2018/2 (Sheldrick, 2015); program(s) used to refine structure: *SHELXL2019*/1 (Sheldrick, 2019); molecular graphics: *ORTEP* 2014.1 (Farrugia, 2012); software used to prepare material for publication: IUCr Journals printCIF.

Acknowledgements

Funding information

References

Figure 1

(22FMB001)

Crystal data

$C_{19}H_{23}F_3IN_3O_3$
M_r = 525.30
Triclinic, $P\overline{1}$
a = 6.6703 (4) Å
b = 10.0801 (6) Å
c = 16.8273 (10) Å
α = 83.649 (2)°
β = 86.849 (2)°
γ = 74.054 (2)°
V = 1080.85 (11) Å^3
Z = 2
F(000) = 524
D_x = 1.614 Mg m^{-3}
Mo $K\alpha$ radiation, λ = 0.71073 Å
Cell parameters from 9733 reflections
θ = 2.3–39.3°
μ = 1.53 mm^{-1}
T = 100 K
Block, clear colourless
0.21 × 0.17 × 0.06 mm

Data collection

Bruker D8 VENTURE PHOTON-III C14 diffractometer
Radiation source: microfocus sealed tube, Incoatec IμS 3.0
Multilayer mirror monochromator
Detector resolution: 7.3910 pixels mm^{-1}
φ or ω oscillation scans
Absorption correction: multi-scan Krause, L., Herbst-Irmer, R., Sheldrick, G. M., Stalke, D. (2015). "Comparison of silver and molybdenum microfocus X-ray sources for single-crystal structure determination" J. Appl. Cryst. 48, 3-10. doi:10.1107/S1600576714022985
T_{min} = 0.85, T_{max} = 0.91
112325 measured reflections
12861 independent reflections
11865 reflections with $I > 2\sigma(I)$
R_{int} = 0.036
θ_{max} = 39.4°, θ_{min} = 2.3°

$h = -11 \rightarrow 11$
$k = -17 \rightarrow 17$
$l = -30 \rightarrow 30$

Refinement

Refinement on F^2
Least-squares matrix: full
$R[F^2 > 2\sigma(F^2)] = 0.020$
$wR(F^2) = 0.048$
$S = 1.05$
12861 reflections
331 parameters
0 restraints
Primary atom site location: dual
Secondary atom site location: difference Fourier map
Hydrogen site location: difference Fourier map
Only H-atom coordinates refined
$w = 1/[\sigma^2(F_o^2) + (0.0198P)^2 + 0.3032P]$
where $P = (F_o^2 + 2F_c^2)/3$
$(\Delta/\sigma)_{max} = 0.004$
$\Delta\rho_{max} = 0.66$ e Å^{-3}
$\Delta\rho_{min} = -0.70$ e Å^{-3}

Special details

Geometry. All e.s.d.'s (except the e.s.d. in the dihedral angle between two l.s. planes) are estimated using the full covariance matrix. The cell e.s.d.'s are taken into account individually in the estimation of e.s.d.'s in distances, angles and torsion angles; correlations between e.s.d.'s in cell parameters are only used when they are defined by crystal symmetry. An approximate (isotropic) treatment of cell e.s.d.'s is used for estimating e.s.d.'s involving l.s. planes.

Fractional atomic coordinates and isotropic or equivalent isotropic displacement parameters (Å^2)

	x	y	z	U_{iso}*/U_{eq}
I1	0.09709 (2)	0.80277 (2)	0.01168 (2)	0.01746 (10)
N1	0.83216 (10)	0.36529 (7)	0.18333 (4)	0.01473 (10)
H1	0.957 (2)	0.3465 (14)	0.1762 (8)	0.018000*
C2	0.68732 (11)	0.46564 (7)	0.13636 (4)	0.01198 (10)
C3	0.72412 (12)	0.55300 (8)	0.07117 (5)	0.01402 (11)
H3	0.863 (2)	0.5490 (14)	0.0526 (8)	0.017000*
C4	0.55197 (12)	0.64821 (7)	0.03486 (4)	0.01401 (11)
H4	0.578 (2)	0.7077 (14)	−0.0099 (8)	0.017000*
C5	0.35109 (11)	0.65516 (7)	0.06533 (4)	0.01265 (10)
C6	0.31411 (11)	0.56671 (7)	0.13132 (4)	0.01197 (10)
H6	0.180 (2)	0.5693 (13)	0.1525 (8)	0.014000*
C7	0.48538 (10)	0.47110 (7)	0.16559 (4)	0.01058 (9)
C8	0.49842 (10)	0.35981 (7)	0.23472 (4)	0.01074 (10)
C9	0.73856 (11)	0.30716 (8)	0.24616 (5)	0.01344 (11)
O10	0.82396 (10)	0.22072 (7)	0.29922 (4)	0.01859 (10)
N11	0.38239 (10)	0.41181 (7)	0.30704 (4)	0.01345 (10)
C12	0.44897 (15)	0.51293 (8)	0.33515 (5)	0.01823 (13)
O13	0.60186 (12)	0.54880 (7)	0.30875 (4)	0.02351 (13)
C14	0.31135 (19)	0.59693 (10)	0.39980 (6)	0.02614 (18)
F15	0.39814 (15)	0.69233 (7)	0.41920 (4)	0.03885 (18)
F16	0.28382 (12)	0.51971 (7)	0.46738 (4)	0.03041 (14)
F17	0.12288 (13)	0.66281 (7)	0.37105 (4)	0.03554 (16)
C18	0.22267 (12)	0.34978 (8)	0.34804 (5)	0.01546 (12)
H18A	0.156 (2)	0.3187 (14)	0.3066 (8)	0.019000*
H18B	0.118 (2)	0.4229 (14)	0.3712 (8)	0.019000*
C19	0.31038 (13)	0.23197 (8)	0.41248 (5)	0.01657 (12)
H19A	0.396 (2)	0.1545 (15)	0.3872 (8)	0.020000*
H19B	0.400 (2)	0.2600 (15)	0.4481 (8)	0.020000*
C20	0.13447 (16)	0.19076 (12)	0.46165 (6)	0.02675 (18)
H20A	0.052 (3)	0.2669 (17)	0.4832 (10)	0.032000*
H20B	0.047 (2)	0.1615 (17)	0.4250 (10)	0.032000*

C21	0.2113 (2)	0.07467 (13)	0.52753 (7)	0.0312 (2)
H21A	0.285 (3)	−0.014 (2)	0.5037 (11)	0.047000*
H21B	0.094 (3)	0.0567 (19)	0.5619 (11)	0.047000*
H21C	0.305 (3)	0.097 (2)	0.5626 (11)	0.047000*
C22	0.41867 (10)	0.24117 (7)	0.20629 (4)	0.01098 (10)
O23	0.26339 (8)	0.27194 (6)	0.16440 (4)	0.01470 (9)
N24	0.53178 (10)	0.11250 (6)	0.22777 (4)	0.01359 (10)
H24	0.629 (2)	0.1024 (14)	0.2583 (8)	0.016000*
C25	0.49394 (13)	−0.01688 (8)	0.20490 (5)	0.01723 (12)
C26	0.48183 (17)	−0.00927 (10)	0.11394 (6)	0.02395 (16)
H26A	0.607 (3)	0.0101 (18)	0.0892 (10)	0.036000*
H26B	0.355 (3)	0.0588 (18)	0.0962 (10)	0.036000*
H26C	0.472 (3)	−0.0933 (18)	0.1002 (10)	0.036000*
C27	0.29397 (19)	−0.03820 (11)	0.24637 (9)	0.0326 (2)
H27A	0.179 (3)	0.036 (2)	0.2309 (11)	0.049000*
H27B	0.304 (3)	−0.041 (2)	0.3043 (12)	0.049000*
H27C	0.266 (3)	−0.123 (2)	0.2337 (11)	0.049000*
C28	0.68293 (18)	−0.13407 (9)	0.23339 (7)	0.02786 (19)
H28A	0.663 (3)	−0.2220 (19)	0.2190 (10)	0.042000*
H28B	0.697 (3)	−0.1384 (19)	0.2908 (11)	0.042000*
H28C	0.808 (3)	−0.1160 (19)	0.2081 (11)	0.042000*

Atomic displacement parameters (Å²)

	U^{11}	U^{22}	U^{33}	U^{12}	U^{13}	U^{23}
I1	0.01787 (2)	0.01155 (2)	0.02040 (2)	−0.00108 (10)	−0.00367 (2)	0.00356 (10)
N1	0.0085 (2)	0.0175 (3)	0.0174 (3)	−0.00300 (18)	−0.00130 (19)	0.0009 (2)
C2	0.0100 (2)	0.0128 (2)	0.0133 (3)	−0.00359 (19)	−0.00009 (19)	−0.0011 (2)
C3	0.0136 (3)	0.0139 (3)	0.0152 (3)	−0.0053 (2)	0.0024 (2)	−0.0013 (2)
C4	0.0171 (3)	0.0121 (2)	0.0133 (3)	−0.0052 (2)	0.0015 (2)	−0.0010 (2)
C5	0.0142 (3)	0.0094 (2)	0.0137 (3)	−0.00236 (19)	−0.0016 (2)	−0.00014 (19)
C6	0.0111 (2)	0.0109 (2)	0.0134 (3)	−0.00243 (19)	−0.0003 (2)	−0.0004 (2)
C7	0.0098 (2)	0.0106 (2)	0.0113 (2)	−0.00281 (18)	−0.00002 (18)	−0.00058 (19)
C8	0.0099 (2)	0.0108 (2)	0.0112 (2)	−0.00230 (18)	−0.00049 (18)	−0.00059 (19)
C9	0.0110 (2)	0.0139 (3)	0.0158 (3)	−0.0038 (2)	−0.0030 (2)	−0.0004 (2)
O10	0.0155 (2)	0.0193 (2)	0.0200 (3)	−0.00409 (19)	−0.00721 (19)	0.0045 (2)
N11	0.0165 (2)	0.0126 (2)	0.0110 (2)	−0.00398 (19)	0.00177 (19)	−0.00155 (18)
C12	0.0285 (4)	0.0144 (3)	0.0123 (3)	−0.0063 (3)	−0.0008 (3)	−0.0018 (2)
O13	0.0349 (4)	0.0228 (3)	0.0187 (3)	−0.0169 (3)	−0.0005 (2)	−0.0037 (2)
C14	0.0437 (5)	0.0170 (3)	0.0157 (3)	−0.0041 (3)	0.0017 (3)	−0.0045 (3)
F15	0.0735 (6)	0.0236 (3)	0.0241 (3)	−0.0180 (3)	0.0022 (3)	−0.0115 (2)
F16	0.0496 (4)	0.0247 (3)	0.0138 (2)	−0.0056 (3)	0.0057 (2)	−0.0031 (2)
F17	0.0456 (4)	0.0247 (3)	0.0256 (3)	0.0092 (3)	0.0019 (3)	−0.0059 (2)
C18	0.0136 (3)	0.0182 (3)	0.0128 (3)	−0.0026 (2)	0.0022 (2)	0.0006 (2)
C19	0.0178 (3)	0.0176 (3)	0.0133 (3)	−0.0046 (2)	0.0009 (2)	0.0015 (2)
C20	0.0231 (4)	0.0326 (5)	0.0212 (4)	−0.0074 (3)	0.0044 (3)	0.0086 (3)
C21	0.0360 (5)	0.0366 (5)	0.0216 (4)	−0.0161 (4)	−0.0009 (4)	0.0113 (4)
C22	0.0099 (2)	0.0108 (2)	0.0123 (2)	−0.00317 (18)	0.00061 (19)	−0.00101 (19)
O23	0.00987 (19)	0.0156 (2)	0.0188 (2)	−0.00359 (16)	−0.00267 (17)	−0.00076 (18)
N24	0.0142 (2)	0.0103 (2)	0.0158 (3)	−0.00238 (18)	−0.00209 (19)	−0.00066 (18)
C25	0.0200 (3)	0.0106 (3)	0.0219 (3)	−0.0059 (2)	0.0013 (3)	−0.0017 (2)
C26	0.0333 (5)	0.0180 (3)	0.0235 (4)	−0.0095 (3)	−0.0026 (3)	−0.0070 (3)

C27	0.0308 (5)	0.0219 (4)	0.0482 (7)	−0.0153 (4)	0.0141 (5)	−0.0037 (4)
C28	0.0328 (5)	0.0119 (3)	0.0351 (5)	0.0003 (3)	−0.0043 (4)	−0.0006 (3)

Geometric parameters (Å, º)

I1—C5	2.0941 (7)	C18—H18B	0.967 (14)
N1—C9	1.3574 (10)	C19—C20	1.5228 (12)
N1—C2	1.3969 (10)	C19—H19A	0.960 (14)
N1—H1	0.807 (14)	C19—H19B	0.983 (14)
C2—C3	1.3837 (10)	C20—C21	1.5189 (14)
C2—C7	1.3970 (9)	C20—H20A	0.910 (16)
C3—C4	1.3981 (11)	C20—H20B	0.990 (16)
C3—H3	0.954 (13)	C21—H21A	1.007 (19)
C4—C5	1.3943 (10)	C21—H21B	0.992 (19)
C4—H4	0.948 (13)	C21—H21C	0.968 (19)
C5—C6	1.4002 (10)	C22—O23	1.2334 (9)
C6—C7	1.3824 (10)	C22—N24	1.3306 (9)
C6—H6	0.940 (13)	N24—C25	1.4884 (10)
C7—C8	1.5127 (10)	N24—H24	0.824 (13)
C8—N11	1.4728 (9)	C25—C27	1.5279 (13)
C8—C9	1.5584 (10)	C25—C28	1.5288 (13)
C8—C22	1.5639 (9)	C25—C26	1.5295 (13)
C9—O10	1.2214 (9)	C26—H26A	0.971 (17)
N11—C12	1.3559 (10)	C26—H26B	0.971 (17)
N11—C18	1.4805 (10)	C26—H26C	0.924 (17)
C12—O13	1.2188 (11)	C27—H27A	0.94 (2)
C12—C14	1.5523 (12)	C27—H27B	0.98 (2)
C14—F15	1.3277 (13)	C27—H27C	0.975 (19)
C14—F17	1.3388 (14)	C28—H28A	0.989 (18)
C14—F16	1.3389 (11)	C28—H28B	0.971 (18)
C18—C19	1.5269 (11)	C28—H28C	0.969 (18)
C18—H18A	0.970 (14)		
C9—N1—C2	111.75 (6)	C20—C19—C18	110.60 (7)
C9—N1—H1	123.2 (10)	C20—C19—H19A	110.6 (8)
C2—N1—H1	125.0 (10)	C18—C19—H19A	108.8 (8)
C3—C2—N1	128.49 (7)	C20—C19—H19B	109.3 (8)
C3—C2—C7	121.66 (7)	C18—C19—H19B	110.5 (8)
N1—C2—C7	109.83 (6)	H19A—C19—H19B	107.1 (11)
C2—C3—C4	117.91 (7)	C21—C20—C19	113.28 (9)
C2—C3—H3	120.6 (8)	C21—C20—H20A	109.8 (10)
C4—C3—H3	121.5 (8)	C19—C20—H20A	108.7 (10)
C5—C4—C3	120.16 (7)	C21—C20—H20B	109.4 (9)
C5—C4—H4	122.2 (8)	C19—C20—H20B	108.3 (9)
C3—C4—H4	117.7 (8)	H20A—C20—H20B	107.2 (14)
C4—C5—C6	121.85 (7)	C20—C21—H21A	110.2 (11)
C4—C5—I1	119.27 (5)	C20—C21—H21B	111.4 (11)
C6—C5—I1	118.88 (5)	H21A—C21—H21B	107.8 (15)
C7—C6—C5	117.37 (6)	C20—C21—H21C	111.8 (11)
C7—C6—H6	119.5 (8)	H21A—C21—H21C	109.1 (15)
C5—C6—H6	123.1 (8)	H21B—C21—H21C	106.3 (15)
C6—C7—C2	121.02 (6)	O23—C22—N24	125.00 (7)

C6—C7—C8	130.44 (6)	O23—C22—C8	119.06 (6)
C2—C7—C8	108.54 (6)	N24—C22—C8	115.90 (6)
N11—C8—C7	113.38 (6)	C22—N24—C25	125.99 (7)
N11—C8—C9	113.12 (6)	C22—N24—H24	117.7 (9)
C7—C8—C9	101.34 (5)	C25—N24—H24	116.2 (9)
N11—C8—C22	110.95 (6)	N24—C25—C27	109.74 (7)
C7—C8—C22	108.31 (5)	N24—C25—C28	105.71 (7)
C9—C8—C22	109.25 (5)	C27—C25—C28	110.65 (8)
O10—C9—N1	127.07 (7)	N24—C25—C26	109.87 (7)
O10—C9—C8	125.08 (7)	C27—C25—C26	111.30 (9)
N1—C9—C8	107.62 (6)	C28—C25—C26	109.42 (8)
C12—N11—C8	113.23 (6)	C25—C26—H26A	109.4 (10)
C12—N11—C18	124.07 (7)	C25—C26—H26B	109.6 (10)
C8—N11—C18	122.48 (6)	H26A—C26—H26B	113.2 (14)
O13—C12—N11	124.06 (8)	C25—C26—H26C	109.0 (10)
O13—C12—C14	117.79 (8)	H26A—C26—H26C	109.5 (14)
N11—C12—C14	117.93 (8)	H26B—C26—H26C	106.0 (14)
F15—C14—F17	107.65 (8)	C25—C27—H27A	110.7 (12)
F15—C14—F16	107.35 (8)	C25—C27—H27B	109.8 (11)
F17—C14—F16	107.85 (9)	H27A—C27—H27B	107.1 (16)
F15—C14—C12	109.69 (9)	C25—C27—H27C	111.9 (11)
F17—C14—C12	110.10 (8)	H27A—C27—H27C	108.0 (16)
F16—C14—C12	113.97 (7)	H27B—C27—H27C	109.2 (16)
N11—C18—C19	113.67 (6)	C25—C28—H28A	107.9 (10)
N11—C18—H18A	106.4 (8)	C25—C28—H28B	110.8 (11)
C19—C18—H18A	111.5 (8)	H28A—C28—H28B	110.1 (15)
N11—C18—H18B	107.8 (8)	C25—C28—H28C	109.1 (11)
C19—C18—H18B	110.1 (8)	H28A—C28—H28C	111.5 (14)
H18A—C18—H18B	107.0 (11)	H28B—C28—H28C	107.5 (15)

C9—N1—C2—C3	175.13 (7)	C9—C8—N11—C12	55.44 (8)
C9—N1—C2—C7	−3.42 (9)	C22—C8—N11—C12	178.62 (6)
N1—C2—C3—C4	−178.33 (7)	C7—C8—N11—C18	125.90 (7)
C7—C2—C3—C4	0.06 (11)	C9—C8—N11—C18	−119.43 (7)
C2—C3—C4—C5	1.28 (11)	C22—C8—N11—C18	3.75 (9)
C3—C4—C5—C6	−1.31 (11)	C8—N11—C12—O13	−6.82 (12)
C3—C4—C5—I1	178.23 (5)	C18—N11—C12—O13	167.96 (8)
C4—C5—C6—C7	−0.06 (10)	C8—N11—C12—C14	167.56 (7)
I1—C5—C6—C7	−179.60 (5)	C18—N11—C12—C14	−17.66 (11)
C5—C6—C7—C2	1.42 (10)	O13—C12—C14—F15	−4.22 (12)
C5—C6—C7—C8	−177.99 (7)	N11—C12—C14—F15	−178.96 (8)
C3—C2—C7—C6	−1.46 (11)	O13—C12—C14—F17	114.07 (10)
N1—C2—C7—C6	177.21 (6)	N11—C12—C14—F17	−60.67 (11)
C3—C2—C7—C8	178.07 (6)	O13—C12—C14—F16	−124.61 (10)
N1—C2—C7—C8	−3.27 (8)	N11—C12—C14—F16	60.65 (12)
C6—C7—C8—N11	−51.43 (10)	C12—N11—C18—C19	−84.56 (9)
C2—C7—C8—N11	129.10 (6)	C8—N11—C18—C19	89.74 (8)
C6—C7—C8—C9	−172.97 (7)	N11—C18—C19—C20	170.01 (7)
C2—C7—C8—C9	7.57 (7)	C18—C19—C20—C21	−179.43 (9)
C6—C7—C8—C22	72.17 (9)	N11—C8—C22—O23	83.48 (8)
C2—C7—C8—C22	−107.29 (6)	C7—C8—C22—O23	−41.57 (8)
C2—N1—C9—O10	−176.98 (8)	C9—C8—C22—O23	−151.13 (7)

C2—N1—C9—C8	8.36 (8)	N11—C8—C22—N24	−98.81 (7)
N11—C8—C9—O10	53.96 (10)	C7—C8—C22—N24	136.14 (6)
C7—C8—C9—O10	175.67 (7)	C9—C8—C22—N24	26.58 (8)
C22—C8—C9—O10	−70.16 (9)	O23—C22—N24—C25	0.64 (12)
N11—C8—C9—N1	−131.25 (6)	C8—C22—N24—C25	−176.91 (7)
C7—C8—C9—N1	−9.54 (7)	C22—N24—C25—C27	−69.02 (11)
C22—C8—C9—N1	104.63 (7)	C22—N24—C25—C28	171.63 (8)
C7—C8—N11—C12	−59.23 (8)	C22—N24—C25—C26	53.66 (10)

Hydrogen-bond geometry (Å, º)

D—H···*A*	*D*—H	H···*A*	*D*···*A*	*D*—H···*A*
N1—H1···O23^{i}	0.807 (14)	1.981 (14)	2.7819 (8)	171.6 (14)
C18—H18*A*···O23	0.970 (14)	2.518 (14)	3.2505 (10)	132.3 (10)
C18—H18*B*···F17	0.967 (14)	2.428 (14)	3.1045 (11)	126.7 (10)
N24—H24···O10	0.824 (13)	2.175 (13)	2.8548 (9)	139.8 (12)
C26—H26*B*···O23	0.971 (17)	2.460 (17)	3.0011 (11)	114.9 (12)

Symmetry code: (i) *x*+1, *y*, *z*.
